# Biological determinants of bladder cancer gene expression subtypes

**DOI:** 10.1038/srep10957

**Published:** 2015-06-08

**Authors:** Mattias Aine, Pontus Eriksson, Fredrik Liedberg, Gottfrid Sjödahl, Mattias Höglund

**Affiliations:** 1 Division of Oncology and Pathology, Department of Clinical Sciences Lund, Lund University, Lund, Sweden; 2 Division of Urological Research, Department of Translational Medicine, Lund University, Malmö, Sweden

## Abstract

Molecular stratification of tumors by gene expression profiling has been applied to a large number of human malignancies and holds great promise for personalized treatment. Comprehensive classification schemes for urothelial carcinoma have been proposed by three separate groups but have not previously been evaluated simultaneously in independent data. Here we map the interrelations between the proposed molecular subtypes onto the intrinsic structure of a rich independent dataset and show that subtype stratification within each scheme can be explained in terms of a set of common underlying biological processes. We highlight novel biological and genomic drivers of urothelial carcinoma molecular subtypes and show that tumors carrying genomic aberrations characteristic of distinct molecular pathways converge on a common top level phenotype corresponding to the two major molecular subtypes of non-muscle invasive disease.

Urothelial carcinoma of the bladder (UC) arises in the transitional epithelium that lines the inner surface of the urinary bladder. Clinically, a major distinction with respect to patient prognosis is made on the basis of whether tumor cells have invaded into the muscle layer beneath the mucosa of the bladder (non-muscle invasive and muscle invasive disease respectively)^1^. Early efforts in molecular profiling of UC established clear differences in the somatic mutation frequencies of *FGFR3* and *TP53* between non-muscle invasive (NMI) and muscle invasive (MI) UC^2^,^3^. On the level of chromosomal aberrations; losses of the p- and q-arms of chromosome 9 as well as homozygous deletions of *CDKN2A* were mainly seen in NMI tumors, while *RB1* deletions and 6p amplifications were more frequent in MI UC^4^,^5^,^6^,^7^,^8^. These findings have led many to consider NMI and MI UC as separate disease entities reached through different paths and defined by distinct sets of genomic aberrations^9^,^10^,^11^. Gene expression profiling has been applied to UC and can reliably separate NMI and MI tumors^12^,^13^,^14^. A number of studies have also produced prognostic gene expression signatures, however with low inter-study replicability and overlap in terms of genes identified^15^. Integrative studies combining copy-number aberrations (CNAs) and gene expression have characterized the associations between the copy-number landscape and gene expression phenotype^16^,^17^. These have identified genomic instability as a major difference between NMI and MI UC and highlighted the existence of an *FGFR3*-*CCND1*-*CDKN2A*-9q circuit operating in NMI UC and a *E2F3*-*RB1* circuit and recurring focal genomic alterations in MI disease.

The concept of intrinsic subtypes was pioneered in breast cancer and has been applied to a large number of human malignancies^18^,^19^,^20^,^21^. Groups at Lund University (Lund), MD Anderson (MDA) and University of North Carolina (UNC) have proposed the existence of intrinsic subtypes of UC^22^,^23^,^24^. The Lund group pioneered molecular classification of UC by proposing a 2-group scheme in 2010, and have subsequently expanded upon these findings in a larger cohort (N = 308) by defining five main molecular subtypes (MS); Urobasal A (MS1a and MS1b), Genomically Unstable (MS2a1 and MS2a2), Infiltrated (MS2b1), Urobasal B (MS2b2.1) and Squamous Cell Carcinoma-like (SCC-like, MS2b2.1)^16^,^24^. Urobasal A tumors were predominantly low-grade papillary NMI tumors and displayed frequent *FGFR3* mutations and infrequent *TP53* mutations. Urobasal B tumors were predominantly of stage ≥ T1, expressed markers of basal urothelium, and were *TP53* mutated. Urobasal B tumors also expressed an activated *FGFR3* pathway signature and carried homozygous *CDKN2A* deletions, hallmarks of NMI UC, indicating Urobasal B tumors as a more aggressive form of Urobasal UC. Genomically Unstable tumors were evenly split between high-grade NMI and MI UC, indicating this subtype as fast-progressing, and were defined by frequent *TP53* mutations, genomic instability, and high proliferation rates. The SCC-like subtype defined a poor-prognosis subset with frequent *TP53* mutations and expression of markers for squamous differentiation in addition to markers of basal cells of the urothelium. The Infiltrated subgroup was defined by expression of immune cell and stromal markers indicative of a component of non-tumor cells in the biopsy sample. The infiltrated subgroup of tumors was therefore not considered an intrinsic subtype of UC and immunohistochemical evaluation of paraffin material showed that malignant cells from the tumors belonging to this group expressed markers characteristic for the other subtypes^24^,^25^.

The UNC group published a two-tier classification system derived from a meta-dataset of 262 MI tumors that included 93 MI tumors from the Lund cohort. The group designed a 47-gene classifier that could separate tumors into the respective classes and validated it in an independent cohort (N = 49). The UNC group termed the two groups Basal and Luminal and showed that the groups differed with respect to expression of urothelial differentiation markers (high in UNC Luminal) and a signature for cells with tumor initiating capacity (high in UNC Basal). Further, the group performed centroid classification using a breast cancer classifier and concluded that UNC Basal UC bears resemblance to basal-like breast cancer, while UNC Luminal UC is similar to the Luminal A subtype of breast cancer^23^.

Similarly to the UNC group, the MDA group relied on a dataset of MI UC tumors (N = 73) to derive three tumor classes. In addition they derived a centroid-based classifier, validated in the MI subset of the Lund cohort (N = 93) as well as an internal validation cohort (N = 57). The MDA classes were termed Basal, Luminal, and TP53-like. The MDA Basal subset of tumors was characterized by squamous differentiation and expression of markers associated with basal urothelial cells. MDA Luminal tumors often displayed papillary growth patterns and were characterized by expression of markers of intermediate and terminally differentiated cells in normal urothelium. MDA TP53-like tumors were proposed to resemble the Luminal group, except in that they express a wild-type TP53-associated gene expression signature. The MDA group also highlighted similarities between the luminal and basal-like subtypes of breast cancer and the MDA Basal and Luminal tumors, and proposed that *TP63* and *PPARG* have opposing roles in defining basal and luminal identities^22^.

To reconcile the proposed subtyping schemes and provide a synthesis of the underlying biology, we overlay the subtype-defining signatures onto the intrinsic structure of an independent dataset of 234 MI UC samples generated by The Cancer Genome Atlas Project Consortium (TCGA)^26^. By consensus clustering and sequential analysis of hierarchical splits, we compare the existing subtype classifiers in a rich independent dataset. Using this integrative approach we are able to explain sample stratification into gene expression subtypes through the combinatorial expression of biological signatures and show that the proposed classification schemes describe UC biology on different levels. Finally, we find evidence for phenotypic convergence between tumors carrying genomic aberrations characteristic of the two major (NMI versus MI) molecular pathways in UC pathogenesis and show that these tumors correspond to the Lund Urobasal A and Genomically Unstable subtypes respectively.

## Results

### A two-group split stratifies samples on the basis of the opposing influences of urothelial- and squamous differentiation

Data on mRNA-based gene expression, somatic copy-number aberrations as well as mutations was obtained for 234 MI UC samples from the TCGA project website and processed as described in the methods section. We extracted gene signatures from the UNC, MDA and Lund studies as well as data on well characterized mutations and CNAs in UC (Methods). As all three groups have published centroid classifiers, we applied these to the independent TCGA data and classified the tumors according to the three respective systems^22^,^23^,^24^.

**Figure 1:**
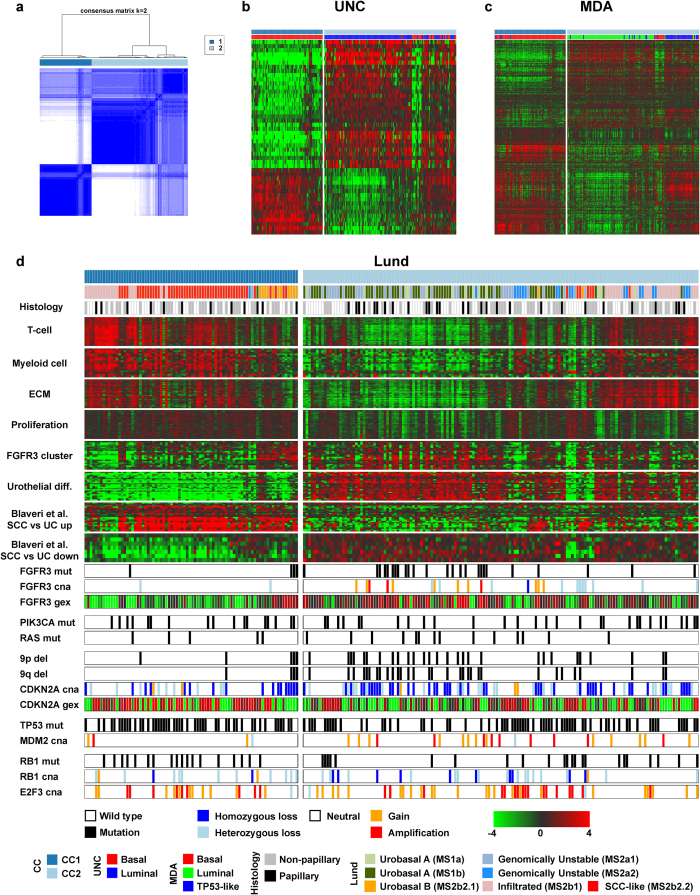
A two-group solution stratifies tumors based on the expression of genes involved in urothelial and squamous differentiation. (**a**) Clustered heatmap of TCGA tumor co-clustering frequencies generated using the ConsensusClusterPlus-package. Blue indicates frequent co-clustering and the color bar indicates the CC1 and 2 subgroups respectively. (**b-c**) UNC and MDA classifier genes in the TCGA data visualized in heatmap format with consensus cluster and subgroup calls indicated. (**d**) Lund classifier calls with histological characteristics and biological gene signatures extracted from Sjödahl *et al.* 2012 and Blaveri *et al.* 2005 as well individual gene expression, CN-status and mutation calls for genes implicated in UC tumorigenesis. Abbreviations: ECM, extracellular matrix; SCC, squamous cell carcinoma; UC, urothelial carcinoma; mut, mutation; cna, copy-number aberration; gex, gene expression; del, deletion.

Based on the UNC paradigm of a 2-group classification system for MI UC, we subjected the TCGA gene expression data to bootstrap hierarchical consensus clustering^27^,^28^. The two-group solution yielded consensus clusters of 82 (CC1) and 152 (CC2) tumors respectively (Fig. 1a). We extracted the UNC classifier genes (N = 47) from the full data matrix and overlaid these on the intrinsic structure of the TCGA data (Fig. 1b). The signature captured two inversely correlated patterns consistent with its original design, i.e. to stratify UC into a Basal or Luminal subgroup^23^. CC1 showed uniform downregulation of two thirds of the signature and upregulation of the remaining third and corresponded to the UNC Basal subgroup (Fig. 1b). The majority of CC2 tumors displayed an inverse pattern to that observed for CC1 and were classified as UNC Luminal. A subset of CC2 tumors expressed either all or none of the classifier genes, leading to a heterogenous appearance in terms of sample classification.

The MDA classifier genes displayed three main patterns across the full set of tumors (Fig. 1c). CC1 tumors were classified as MDA Basal and showed upregulation of approximately half of the signature genes. CC2 tumors were classified as MDA Luminal and displayed gene expression patterns inversely correlated to those of CC1 tumors. An exception to this pattern was observed for a subset of CC2 tumors that shared features of CC1 tumors and were classified as MDA Basal and TP53-like. CC2 samples classified as MDA Basal or TP53-like corresponded to the subset of tumors that did not conform to the UNC Basal or Luminal paradigm (Fig. 1b,c).

The Lund molecular subtypes were originally derived from a dataset that included the full pathological spectrum of UC, and defined robust gene expression signatures linked to distinct biological processes^24^. We extracted seven gene signatures strongly associated with biological processes showing differential expression across the Lund molecular subtypes (Methods)^12^,^24^. Based on UC genomic circuits described by Lindgren *et al.* 2012^17^ we extracted data on mutations affecting *FGFR3*, *PIK3CA*, the *RAS* genes, *TP53* and *RB1* as well as CNAs affecting *FGFR3*, *CDKN2A*, *MDM2*, *RB1*, and *E2F3* (Methods). We also included histological data for tumors included in the original TCGA study^26^.

At the two-group level we did not observe a significant association with papillary histology for either subgroup. CC1 tumors were enriched for the Lund Urobasal B and SCC-like subtypes (11 of 13 and 50 of 57 respectively, both p < .001), and displayed robust expression of genes related to T- and myeloid cell as well as extracellular matrix (ECM) function, consistent with immune- and stromal cell infiltration in the biopsy sample (Fig. 1d). CC2 tumors were classified as Urobasal A or Genomically Unstable and displayed low expression levels of the same signatures, indicating a relatively higher tumor cell content or less immunogenic or stroma-infiltrated phenotype. A significant number of CC2 tumors did however show expression of infiltration-related genes, a pattern also captured by the UNC and MDA classsifiers (Fig. 1b–d). The majority of this subset was accordingly classified as Lund Infiltrated. CC1 tumors showed higher expression levels of proliferation-related genes compared to CC2, however high proliferation was also observed for a subset of CC2 tumors (Fig. 1d). *FGFR3* signature expression was a hallmark of Urobasal A and B tumors in the Lund study^24^. Consistent with the presence of Urobasal tumors in the TCGA dataset, we observed *FGFR3* signature expression in a substantial proportion of CC2- and a minority of CC1 tumors. The *FGFR3* signature expressing tumors were classified as Lund Urobasal A and B subtypes (Fig. 1d). CC1 tumors displayed universally low expression levels of a urothelial differentiation signature, enriched for lipid metabolism-related genes^24^. The majority of CC1 tumors were classified as Lund SCC-like. A gene signature designed to separate squamous cell carcinoma (SCC) of the bladder from UC^12^ showed high expression in a majority of CC1 tumors, further supporting an SCC-like identity for this subset (Fig. 1d). A minority of CC2 tumors show expression of infiltration-related signatures at levels comparable to CC1 tumors (Fig. 1d). However, these tumors do not express genes upregulated in SCC while they express a signature of differentiated urothelium, leading to a CC2 identity and Lund Infiltrated classification. We confirmed our conclusions regarding the biological themes driving sample stratification by performing gene ontology analysis on differentially expressed genes (Methods) between the two groups using AmiGO^29^(Supplementary table 1).

*FGFR3* mutations, CNAs, and *FGFR3* gene expression was mainly confined to CC2 tumors, consistent with expression of the full *FGFR3* signature (Fig. 1d). The two subgroups did not differ with respect to *PIK3CA* or *RAS* mutations. CC2 tumors were however enriched for chromosome 9 p- and q-arm losses as well as *CDKN2A* deletions (28, 31 and 70 respectively of 152 CC2 tumors, all p < 0.05). As *FGFR3* mutations as well as losses of 9p and 9q are frequently observed in Lund Urobasal NMI tumors, a subset of CC2 tumors may thus represent progressed NMI tumors. *TP53* pathway inactivation is a common feature of aggressive UC and is frequently accomplished by *TP53* mutation or *MDM2* amplification^2^,^30^. *TP53* mutation frequencies did not differ between CC1 and CC2, and although *MDM2* gains and amplifications were significantly more common in CC2 tumors (22 of 25 amplifications, p < 0.05), there was no significant difference in overall pathway inactivation between the two groups. No significant differences between the groups were observed for *RB1* disruption or *E2F3* amplifications, either alone or combined.

In summary, we find evidence for urothelial and squamous differentiation combined with immune/ECM infiltration being the major factor driving stratification into two groups. Consequently, the two-group UNC classifier stratifies samples on the basis of urothelial differentiation markers and immune/ECM related gene expression. Expression of infiltration related genes in the UNC signature act as a positive marker for the Basal subset, while the remaining genes in the signature counter select for urothelial differentiation. The MDA signature consists of three main components; urothelial differentiation, immune and ECM infiltration, as well as squamous differentiation. In the two-group split, infiltration and squamous differentiation are positive markers for the CC1 subgroup while urothelial differentiation related genes are downregulated. Thus CC1 tumors correspond to the UNC and MDA Basal subgroups while CC2 tumors are classified as UNC Luminal and MDA Luminal and TP53-like. At the two-group level, the CC1 tumors largely correspond to the Lund SCC-like subtype, while CC2 tumors are classified as Urobasal A and Genomically Unstable.

### Substratification highlights phenotypic convergence and novel UC subtypes

**Figure 2:**
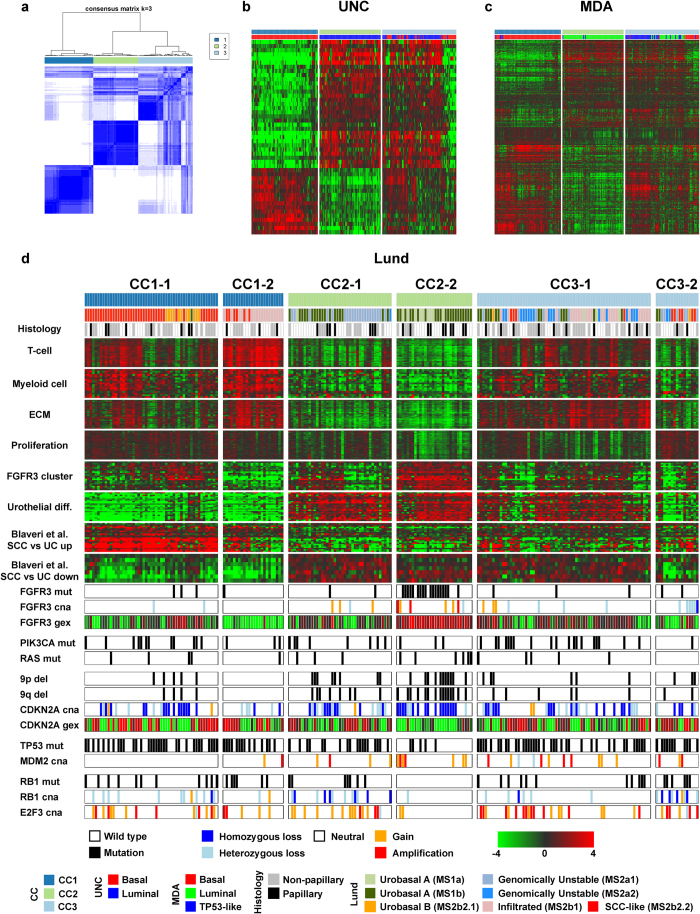
Substratification reveals biologically coherent themes to gene expression subtypes. (**a**) Clustered heatmap of sample co-clustering frequencies generated using the ConsensusClusterPlus-package. Blue indicates frequent co-clustering and the color bar indicates the CC1 (dark blue), 2 (light green) and 3 (light blue) subgroups respectively. (**b-c**) UNC and MDA classifier genes in heatmap format overlaid on the three TCGA subgroups with subgroup calls indicated. (**d**) Further stratification of the CC1, CC2 and CC3 groups into six subgroups with Lund classifier calls, histological characteristics as well as biological gene signatures extracted from Sjödahl *et al.* 2012 and Blaveri *et al.* 2005 as well CN-status and mutation calls for genes implicated in UC tumorigenesis. Abbreviations: ECM, extracellular matrix; SCC, squamous cell carcinoma; UC, urothelial carcinoma; mut, mutation; cna, copy-number aberration; gex, gene expression; del, deletion.

Significant heterogeneity was observed within the CC2 tumors in the two-group split. In addition, the MDA classification system stratifies MI UC into three groups^22^. We therefore repeated the consensus clustering, and chose a three-group solution to explore additional structure in the dataset. The three-group split was highly similar to the dichotomous scheme, except in that a subset of CC2-tumors formed a new separate cluster (CC3) that also included five former CC1 samples. The three-group split thus consisted of 77 CC1, 71 CC2, and 86 CC3 tumors respectively (Fig. 2a). The UNC classifier genes were differentially expressed between CC1 and CC2 tumors, CC3 tumors however did not conform to the Basal/Luminal paradigm (Fig. 2b). The three components of the MDA classifier showed different combinatorial patterns between the three groups (Fig. 2c). The CC1 group of tumors corresponded to the MDA Basal subgroup. CC2 tumors were classified as MDA Luminal and were enriched for papillary histology (17 of 32 with data, p < 0.01). The CC3 subgroup included the majority (42/47) of MDA TP53-like tumors, but also a substantial number of MDA Basal and Luminal tumors.

Inspection of the MDA signature genes within the consensus clusters revealed substantial heterogeneity within each of the three subgroups (Fig. 2c). To explore this additional structure, we performed 2-group splits within each of the three top-level clusters yielding a total of six subgroups (Supplementary Figure 1a–c). The UNC signature genes were not differentially expressed within the CC1 or CC2 subclusters. However within CC3, the newly formed CC3-1 subcluster showed high levels of expression for all while CC3-2 tumors did not express any UNC signature genes indicating that the classifier calls for this subset of tumors are unreliable (Supplementary Figure 1d). A substantial number of MDA classifier genes displayed differential expression between the CC1-, CC2- and CC3 subsplits indicating that a three-group solution is insufficient to capture the full biological spectrum of MI UC (Supplementary Figure 1e). The MDA classification scheme was designed to stratify UC tumors into three groups and could as such not identify the CC1 and CC2 subsplits. The larger CC3-1 group included tumors of all MDA subgroups and the CC3-2 tumors were predominantly classified as MDA Basal.

The CC1 subsplit stratified tumors mainly on the basis of expression of genes involved in squamous differentiation (CC1-1 upregulated) as well as genes involved in immunological and ECM processes (Fig. 2d). The CC1 subgroups exhibited uniform and high expression of cell cycle genes and did not express urothelial differentiation signature genes. The majority of CC1-1 tumors showed high expression of squamous differentiation markers and low expression of the urothelial differentiation signature and were classified as Lund SCC-like (Fig. 2d). A subset of CC1-1 tumors showed low relative immune- and ECM signature expression with concomitant expression of the *FGFR3* signature. *FGFR3* signature expressing CC1-1 tumors displayed frequent homozygous deletions of *CDKN2A* and lacked *RB1*/*E2F3* alterations. These observations are consistent with the Lund Urobasal B classification for this subset of tumors that display features of urothelial and squamous differentiation (Fig. 2d)^17^,^24^. The CC1-2 group of tumors expressed immune and ECM gene signatures, displayed high *VIM* and low *CDH1* expression, and may thus represent a novel “Infiltrated mesenchymal” subtype of UC (Supplementary Figure 2a). CC1-2 tumors were completely devoid of large-scale aberrations on chromosome 9, and only infrequently carried *CDKN2A* deletions. The gene signature expression patterns and Lund classifier designations suggest that CC1-2 tumors are a subset of Lund Infiltrated tumors that lack expression of both urothelial- and squamous differentiation genes. Whether this phenotype represents an intrinsic subtype of UC, i.e. are the signature genes expressed by tumor cells, or by infiltrating non-tumor cells, would require assessment using immunohistochemical methods.

CC2-1 tumors were enriched for the Lund Genomically Unstable subtype (22 of 41 tumors, p < 0.001) and had lower and more variable *FGFR3*-signature expression compared to CC2-2 (Fig. 2d). Consistent with high *FGFR3* signature expression, CC2-2 tumors were strongly enriched for *FGFR3* mutations and CNAs (21 of 30 tumors, p < 0.001) as well as the Urobasal A subtype (all tumors, p < 0.001) and papillary histology (10 of 14 with data, p < 0.01). CC2-2 tumors also displayed more frequent of *CDKN2A* as well as chromosome 9p and 9q losses compared the other subgroups (19, 11 and 15 respectively of 30 tumors, all p < 0.001). CC2-1 was enriched for *RB1* aberrations (16/41 versus 1/30, p < 0.001) as well as *TP53* mutations (18/41 versus 5/30, p < 0.05) compared to CC2-2. The overall TP53-pathway inactivation levels did not differ between the CC2 subgroups as *MDM2* amplifications were more common in the CC2-2 group. *FGFR3* mutations are generally thought of as aberrations found in NMI tumors and represent the low-grade papillary pathway in UC. Conversely, *RB1* aberrations and *TP53* mutations are frequently observed in high-grade CIS and MI lesions and are postulated to arise through a different process than the one leading to NMI UC^11^. In the CC2 subgroup however, the different proposed pathways seem to converge on a common top-level phenotype.

CC3-1 tumors showed consistent high expression of ECM signature genes and variable T- and myeloid cell signatures (Fig. 2d). CC3-1 tumors also showed moderate but consistent expression of the urothelial- and *FGFR3* signatures but were not enriched for specific genomic aberrations. A substantial proportion of CC3-1 tumors were classified as Lund infiltrated. Unlike the CC1-2 “Infiltrated mesenchymal” subtype, CC3-1 tumors however express *CDH1* at significantly higher levels than CC1-2 tumors (p < 1.1 × 10^−6^, MW-test, Supplementary Figure 2a) and may therefore represent an “Infiltrated epithelial” subtype. We substantiated the identities of CC3-1 “Infiltrated epithelial” and CC1-2 “Infiltrated Mesenchymal” tumors by comparing expression levels of genes characteristic of epithelial-mesenchymal-transition (EMT) as well as epithelial- and urothelial differentiation and found good agreement with our chosen nomenclature (Supplementary Figure 2b–d). Additionally AmiGO-analysis on genes upregulated in CC3-1 versus CC1-2 showed significant enrichment of GO-terms related to lipid metabolism and epithelial differentiation (Supplementary table 2). CC3-2 tumors expressed cell-cycle genes highly, and were the only subgroup in which the literature-derived signatures did not capture additional positive biological features (Fig. 2d). CC3-2 tumors were enriched for *RB1* aberrations (11 of 17 tumors) when comparing with CC3-1 or all other subgroups combined (both p < 0.01, Fisher’s exact test). Given the association of *RB1* aberrations with aggressive disease and the high expression of proliferation associated genes, this subset may represent a rare and highly proliferative subset of undifferentiated tumors.

By further substratification of the three main groups of tumors we provide an additional level of biological interpretation of the processes underlying UC subtypes. We show that tumors belonging to the CC2 group can be further divided into subgroups showing differential expression of biological signatures with strong associations to the Lund Genomically Unstable and Urobasal subtypes, indicating phenotypic convergence of two distinct molecular phenotypes representative of different paths of urothelial tumorigenesis. This conclusion is further substantiated by the differential mutation and genomic aberration spectrum of the respective subgroups. We also found that CC1 tumors may be split on the basis of expression of genes involved in keratinization and epidermal differentiation as well as immune- and ECM processes. The CC1-1 subgroup corresponds to the Lund SCC-like subtype characterized by squamous differentiation, while the CC1-2 subgroup corresponds to a subset of Lund Infiltrated tumors with a “mesenchymal” phenotype. The CC3-1 subgroup corresponds to Lund Infiltrated tumors expressing urothelial differentiation genes as well as Urobasal and Genomically Unstable tumors with high-level expression of immune- and ECM genes. CC3-2 tumors may represent a novel subtype of highly proliferative undifferentiated tumors previously uncharacterized due to cohort size or composition effects.

### Biological signatures defining UC gene expression subtypes

**Figure 3:**
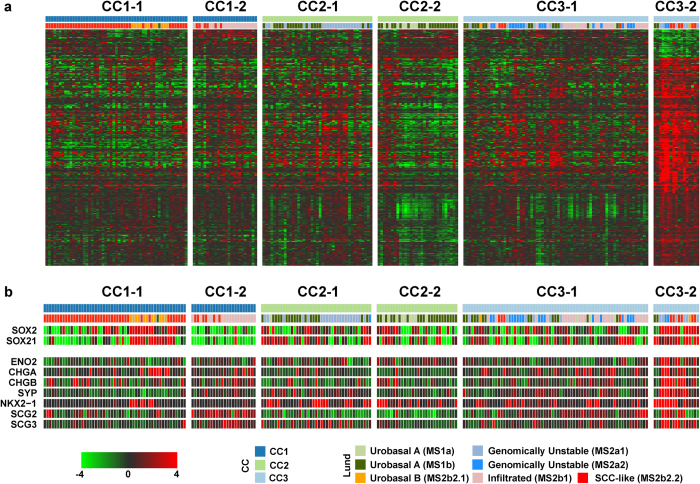
The CC3-2 subgroup exhibits high expression of cell cycle genes and markers of variant histologies. (**a**) Clustered heatmap visualization of genes differentially expressed between CC3-2 and the remaining subgroups with consensus clusters and Lund subtypes indicated. (**b**) CC3-2 and CC1-1/Lund Urobasal B tumors express the pluripotency markers *SOX2* and *SOX21*. CC3-2 tumors exhibit consistent high expression of genes characteristic of variant histologies of urothelial cancers.

To characterize the CC3-2 group further, we derived lists of genes specifically up- (N = 388) or downregulated (N = 53) in this subgroup (Fig. 3a, Methods). AmiGO-analysis revealed enrichment of cell-cycle related genes in the upregulated set, indicating a proliferative phenotype (Supplementary table 3). In addition this subgroup expressed high levels of pluripotency-associated genes *SOX2* and *SOX21*^31^,^32^, pointing to a stem-like undifferentiated phenotype. Though significantly higher in the CC3-2 group, we also observed high *SOX2* and *SOX21* expression in CC1-1 tumors classified as Lund Urobasal B (Fig. 3b). We also found high expression of small-cell/neuroendocrine markers *ENO2*, *CHGA*/*CHGB*, *SYP*, *NKX2-1*, and *SCG2*/*SCG3* in CC3-2 tumors (Fig. 3b)^33^,^34^,^35^,^36^. This small but distinct subgroup may represent a small-cell/neuroendocrine-like tumor phenotype.

**Figure 4:**
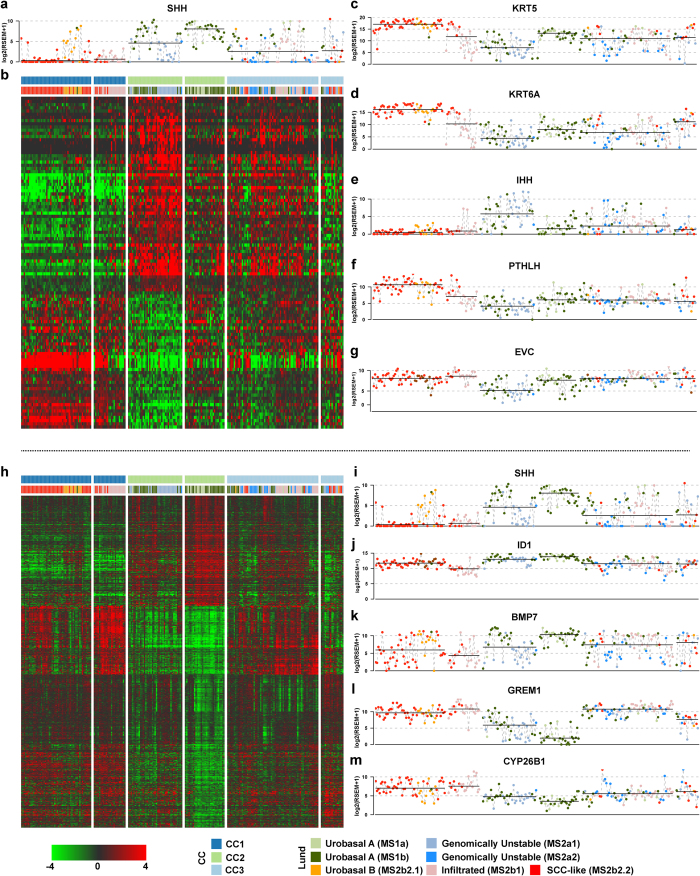
Differential expression of hedgehog-linked genes within the CC2 cluster. (**a**) Uncentered data on SHH expression accross the consensus subgroups with Lund classification indicated for each tumor. The solid black bar indicates the within-cluster median gene expression level. (**b**) Heatmap visualization of genes differentially expressed between CC2-1 and the remaining subgroups. (**c–g**) As in (**a**) for the CC2-1 downregulated genes *KRT5*, *KRT6A*, EVC and *PTHLH* as well as the upregulated gene *IHH*. (**h**) As in (**b**) for genes differentially expressed between CC2-2 and the remaining subgroups. (**i–m**) As in (**a**) for the genes *SHH*, *ID1*, *BMP7*, *GREM1* and *CYP26B1*.

Recent efforts using mouse models have yielded insights into urothelial development and tumorigenesis^37^,^38^,^39^,^40^,^41^. A subset of these studies has focused on the prominent role of SHH-signaling in urothelial regeneration and tumor formation^38^,^39^. Using a subset of the present TCGA cohort, Shin *et al.* 2014 showed that *SHH* and hedgehog pathway gene expression is significantly higher in tumors described as “papillary-like” compared to the remaining tumors, and proposed that loss of *SHH* expression is a prerequisite for and a ubiquitous feature of invasive UC^40^. The *SHH*-expressing subset of tumors corresponds to the CC2-2 cluster. We also found *SHH* expression significantly reduced in the remaining subtypes (Fig. 4a), consistent with recent findings^40^. We noted a significant drop in *SHH* mRNA levels between the CC2-1 and CC2-2 subgroups, which was associated with Lund Genomically Unstable as opposed to Urobasal A classification status (Fig. 4a). Reinforcing the link between *SHH* and a Urobasal identity, CC2-1 tumors classified as Urobasal B showed signs of *SHH* expression. Shin *et al.* linked the varying levels of *SHH* expression in TCGA tumors to presence of normal urothelium or a residual responsiveness to SHH/BMP signaling^40^. While feasible, this hypothesis does not account for the observed differences in the underlying genomic alterations or Lund gene expression subtypes. We performed statistical tests to derive a list of genes specifically up- (N = 61) or downregulated (N = 43) in the CC2-1 group (Fig. 4b, Methods). The list of CC2-1 downregulated genes included *KRT5* and *KRT6A-C*, suggesting a phenotype characterized by low expression of genes expressed in the Lund Urobasal or SCC-like subtypes and consistent with the observed Genomically Unstable classification (Fig. 4c,d). CC2-1 upregulated genes included *IHH*, one of the three human hedgehog homologs. Within the CC2 subgroup, high *IHH* expression was restricted to tumors with low *SHH* expression (Spearman’s Rho = −0.31, p < 0.01), and only showed sporadic expression in the other subgroups (Fig. 4e). The role of *IHH* has not been characterized in the context of urothelial development and carcinogenesis. However in support of a putative role for *IHH* signaling in UC we found specific downregulation of *PTHLH*^42^ and EVC^43^ in CC2-1 tumors (Fig. 4f,g).

To further characterize the CC2-2 subgroup, we identified specifically up- (N = 511) or downregulated (N = 1030) genes within this subgroup (Fig. 4h, Methods). CC2-2 upregulated genes were enriched for protein synthesis, processing and targeting, while the downregulated genes were involved in proliferation (Supplementary table 4). Shin *et al.* provided evidence for urothelial SHH- and stromal BMP expression impeeding UC progression^40^. In addition to the previously reported association between *SHH* and *ID1* expression in TCGA data^40^ (Fig. 4i,j), we found a strong correlation to *BMP7* expression (Spearman’s Rho = 0.62, p < 0.001, Fig. 4k). Functional links between the two genes have been established in the context of developmental morphogenesis^44^,^45^ and *BMP7* expression is regulated by *SHH*^46^. In further support for a role of hedgehog/BMP signaling, we found specific downregulation of *GREM1* in CC2-2 tumors as well as overall lower expression levels in CC2 tumors (Fig. 4l). Importantly *GREM1* expression is repressed in *SHH*-lineage cells^47^ potentially through active FGF signaling^48^. Finally, we also found specific downregulation of *CYP26B1* in CC2-2 tumors (Fig. 4m), a key enzyme in retinoic acid (RA) inactivation. RA deficiency has been shown to induce keratinizing squamous metaplasia in rodents^49^. Consistent high expression of *CYP26B1* was observed in CC1 tumors, suggesting that impairment of RA signaling may be a driver of the SCC-like phenotype (Fig. 4m). Given that RA is a key factor in urothelial differentiation^37^ and that aberrant SHH signaling is a prominent feature of urothelial tumorigenesis^39^,^40^ our findings suggest that active SHH/RA signaling may underlie the Lund Urobasal tumor phenotype. In summary our results indicate the existence of a UC phenotype characterized by high expression of cell cycle genes and markers of small-cell/neuroendocrine histologies, link *SHH* expression to the Lund Urobasal A tumor phenotype, and suggest that *IHH* signaling may also be involved in UC tumorigenesis.

## Discussion

We have mapped the interrelations of the three proposed gene expression based classification systems in a large independent cohort of tumors. In addition to the three classifiers, TCGA consortium performed clustering of tumors based on mRNA expression and found four main clusters of tumors (Cluster I-IV)^26^. TCGA used a descriptive nomenclature for their clusters and described Cluster I as “papillary-like”, Cluster III as “basal/squamous-like”, Cluster I and II tumors were described as expressing markers of urothelial differentiation while characteristics of Cluster IV were not expanded upon.

**Figure 5:**
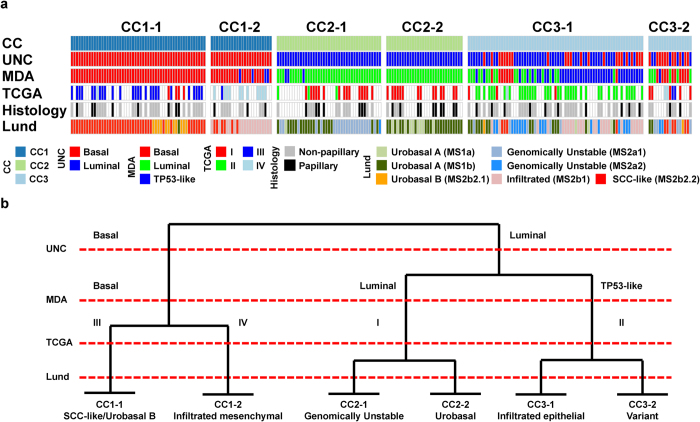
Interrelations between four classification schemes for UC. (**a**) Consensus cluster subgroups with UNC, MDA, TCGA and Lund classes as well as histology indicated for tumors (columns) included in the present study. (**b**) A schematic representation detailing how the different proposed subtypes of UC are interrelated.

Expectedly, our current stratification scheme corresponds well to TCGA clusters (Fig. 5a). TCGA Cluster I corresponds to the CC2 subgroup, but does not differentiate between CC2-1/Genomically Unstable and CC2-2/Urobasal tumors. This cluster also corresponds to the UNC and MDA Luminal subgroups. Cluster II tumors correspond to the CC3-1/”Infiltrated epithelial” subgroup, show mixed UNC classification and are mainly comprised of MDA Luminal and TP53-like tumors. All three classification systems exhibit marked heterogeneity within the CC3 subgroup, and this is also the least stable in terms of sample co-clustering. A potential explanation for the relative instability of CC3 could be the effects of intrinsic- (tumor phenotype) and extrinsic (e.g. immune cell infiltration) factors affecting sample clustering. Cluster III tumors correspond to CC1-1/SCC-like tumors and are UNC and MDA Basal. Cluster IV tumors correspond to the CC1-2/”Infiltrated mesenchymal” subgroup and are also classified as UNC and MDA Basal.

We conclude that a broad agreement exists between the three proposed classification systems for UC as well as the TCGA classes. Ultimately the conclusions drawn in each individual study relate to the cohort stage composition and basic questions posed by the respective investigators. The UNC group based their interpretation on the highest level split in an MI UC dataset and therefore designed a classifier optimized to make this distinction. The MDA researchers derived their classification system using gene expression data for 73 MI tumors, limiting the resolution to identify more than three subgroups. The Lund subtypes were derived using the largest dataset compiled to date and the cohort was selected to cover the full stage and grade distribution of UC. The Lund subgroups are therefore found at a lower level in the hierarchical tree than the UNC and MDA classes, and thus capture more detailed aspects of UC biology (Fig. 5b). As the Lund study was the only one to include NMI tumors, and centroid-based classifiers are sensitive to the cohort composition, the Lund classifier alone captures the CC2-1/Genomically Unstable and CC2-2/Urobasal distinction.

Both the UNC and MDA classifiers assign CC1 tumors a Basal identity. The Lund classification system however distinguishes between CC1-1 (Lund SCC-like/Urobasal B) tumors expressing markers of squamous differentiation and CC1-2/”Infiltrated mesenchymal” (Lund Infiltrated) tumors that do not. Using immunohistochemistry, we have shown that samples classified as Lund Infiltrated/MDA TP53-like often display a Genomically Unstable or SCC-like tumor cell phenotype^25^. Hence, it should be challenged whether Lund Infiltrated/MDA TP53-like tumors represent intrinsic or composite phenotypes.

The more granular Lund classification system can separate TCGA-I as well as UNC and MDA Luminal tumors (CC2) into subtypes characterized by *TP53*/*RB1*- or by chromosome 9/*FGFR3* alterations, with potential implications for targeted interventions in the NMI setting in which these tumors constitute the majority of cases^24^,^25^. Ultimately one must however separate the concepts of clinical utility and biological completeness. A classification system implemented in the clinical setting does not necessarily have to capture the full biological spectrum of UC, and a two- or three-tier stratification system may prove clinically sufficient in the MI setting. The specific molecular pathways underlying a top level phenotype may however interact with or modulate the clinical course of disease. In this context, the phenotypic convergence of Urobasal A and Genomically Unstable tumors within CC2, or Urobasal B and SCC-like tumors in CC1-1 may be of significance.

In summary, we use multilevel public data to map the interrelations between the Lund, UNC, and MDA classification systems for UC. We map biological themes of subtype-defining gene expression patterns as well as genomic aberrations and show that these provide a coherent explanation for tumor stratification. We also provide strong evidence in favor of substratification of MI UC into more than three distinct entities. Further, we show that whereas Lund Urobasal tumors may be defined by active SHH signaling, Lund Genomically Unstable tumors instead show signs of IHH expression. With respect to hedgehog signaling in UC molecular subtypes, we expect that studies utilizing animal models of UC will yield valuable insight into the biological processes underlying UC development and progression. A limitation of the current study is the lack of large independent datasets characterized on the levels of gene expression, copy-number aberrations and somatic mutations, making independent validation of novel results infeasible. In addition to the continued generation of large and well-characterized cohorts, future investigations in the field of molecular subtyping of bladder cancer should recognize that the clinical distinction between NMI and MI UC may not be well founded in biology, and that cohort size and stage composition affect conclusions drawn from molecular studies. We also emphasize that all UC subtyping schemes proposed to date capture aspects of the same underlying biological processes on different levels, a fact that should facilitate the establishment of consensus subtypes for UC.

## Methods

### TCGA data acquisition and processing

We retrieved data on gene expression (RNA-seq), CNAs (SNP6-arrays), and somatic mutations (DNA-seq) from TCGA (files listed in Supplementary Table 5). Only samples for which data was available on all three levels were included (N = 234). Gene expression estimates were derived using RSEM^50^ and within-sample normalized to a fixed upper quartile by TCGA. The data was further processed by adding the constant 1 followed by log2 transformation. For CN-estimates we used copy-number-variant filtered data mapped to HG19 coordinates. Gene models were extracted from the TCGA GAF2.1 (TCGA.hg19.June2011.gaf) and for each sample, gene-level CN-estimates were defined as the average segmentation level across the length of the gene model. The CN data was further processed by binning into the categories homozygous loss (CN < −0.8 ), hemizygous loss (CN < -0.4), neutral (−0.4 ≤ CN ≤ 0.4), gain (CN > 0.4), amplification (CN > 1.2). Loss of 9p and 9q was determined using a cutoff of at least 50% of genes affected on the respective chromosome arms. For somatic mutations we used data generated by an automated mutation calling pipeline in order to maximize the number of included samples. The mutations were filtered of duplicate calls, synonymous as well as noncoding variants, and dichotomized on the sample and gene level. Supplementary Table 6 lists by-sample annotations for gene mutations and genomic aberrations.

### Subtype classification and biological signature extraction

Centroids for sample classification were derived in the Lund, MDA and UNC datasets respectively^22^,^23^,^24^. Gene symbols in the respective datasets were updated to current nomenclature^51^ and matched to the independent TCGA data. Each tumor sample was assigned subtype identities using the nearest centroid method with Pearson correlation as the similarity metric (Supplementary Figure 3 and Supplementary Table 6). Biological signatures were obtained from the respective publications^12^,^22^,^23^,^24^ and mapped to the TCGA gene expression data by official gene symbol. The UNC and MDA signatures correspond to the centroid classifier genes. The genes included in each signature that could be mapped to TCGA gene expression data are listed in Supplementary Table 7.

### Bootstrap hierarchical clustering

Prior to hierarchical clustering, low-varying genes were filtered out by applying an inter-quartile range (IQR) threshold of log2 1.5. The resulting matrix consisted of expression values for 6689 genes and was used in all subsequent clusterings. For bootstrap hierarchical clustering we used ConsensusClusterPlus^28^ in R^52^ with 2000 iterations, Pearson correlation as the similarity metric, and Ward’s algorithm for clustering. For the top level split we focused on the two- and three group solutions in accordance with the UNC and MDA approaches to UC classification^22^,^23^. The three top level subgroups (CC1, CC2 and CC3) were further split using the same method and settings as described above and a two-group solution was selected for each, yielding a total of six subgroups. Supplemetary table 5 lists sample consensus cluster annotations.

### Statistical analyses and data visualization

Statistical enrichment analyses for molecular subtypes, genomic aberrations and mutations with respect to tumor consensus cluster subgroups were performed using a two-tailed Fisher-test, a p-value below 0.05 was considered significant. For determining differentially expressed genes in two-group comparisons we used one-versus-rest Mann-Whitney (MW) tests, p-values were adjusted for multiple testing using the method of Benjamini and Hochberg^53^. An adjusted p-value below 0.01 and a absolute median fold difference of 0.5 was set as the criterion for differential expression. Specific up- or downregulation of genes in the CC2-1-, CC2-2- and CC3-2 subgroups was determined by first performing a one-versus-rest MW-test as described above. The list of significant genes was further subjected to post-hoc MW-tests comparing the expression level within the subgroup in question to each of the other five subgroups and applying additional median fold difference (all absolute delta > 0.5) and p-value (all p < 0.01) criteria. Enrichment analyses were performed using the AmiGO^29^ web tool with default settings. All statistical analyses and data visualization was performed in the R programming environment^52^. Gene expression heatmaps were pseudocolored using 100 color bins with eight logs (base 2) between the extremes, rows were clustered using Pearson distance and Ward’s algorithm.

## Additional Information

**How to cite this article**: Aine, M. *et al.* Biological determinants of bladder cancer gene expression subtypes. *Sci. Rep.* **5**, 10957; doi: 10.1038/srep10957 (2015).

## Supplementary Material

Supplementary Information
