# Supplementary material for: Biological determinants of bladder cancer gene expression subtypes
Source: Sci Rep. 2015 Jun 8;5:10957. doi: 10.1038/srep10957 (PMC4650643; doi:10.1038/srep10957)
Supplement: Supplementary Information [file srep10957-s1.pdf]

Supplementary Information

Biological determinants of bladder cancer gene expression subtypes

Mattias Aine, Pontus Eriksson, Fredrik Liedberg, Gottfrid Sjödahl, Mattias Höglund

Supplementary Figure Legends

Supplementary Figure 1 | Substratifcation of the three main subgroups of tumors. (**a-c**) Heatmap visualization of sample coclustering frequencies for two-group splits of the CC1 (**a**), CC2 (**b**), and CC3 (**c**) subgroups generated using ConsensusClusterPlus in R. (**d-e**) Heatmap visualization of the UNC- (**d**) and MDA (**e**) classifier genes accross the six consensus subgroups.

Supplementary Figure 2 |CC1-2 subgroup tumors exhibit high VIM and variable CDH1 expression. Uncentered mRNA expression levels of the genes *VIM* (Vimentin) and *CDH1* (E-cadherin) accross the consensus cluster subgroups with Lund molecular subtype indicated **(a)**. Boxplots showing relative difference in mRNA exprssion between the CC1-2 and CC3-1 subgroups for genes related to EMT **(b)**, epithelial differentiation **(c)** and urothelial differentiation **(d)**.

Supplementary Figure 3 | UC gene expression classifier calls accross TCGA tumor samples. TCGA samples were classified using UNC, MDA and Lund gene experssion centroids wiht Pearson correlation as the similarity metric. Final sample-level classifier calls and correlations to the respective centroids are shown in heatmap format for the three classification systems and consensus cluster subgroups.

Supplementary Figures

Supplementary Figure 1 - Höglund

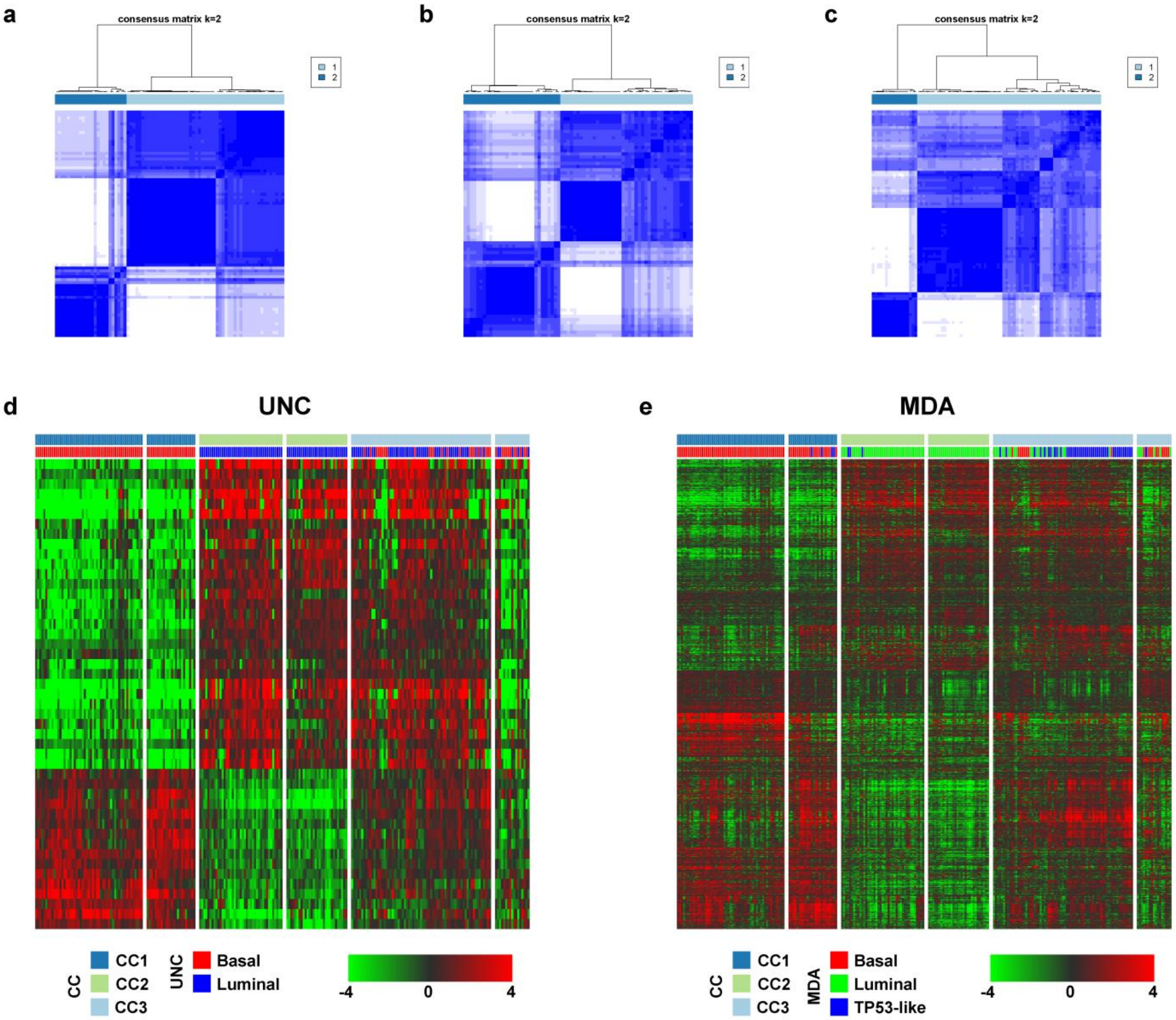

Supplementary Figure 2 - Höglund

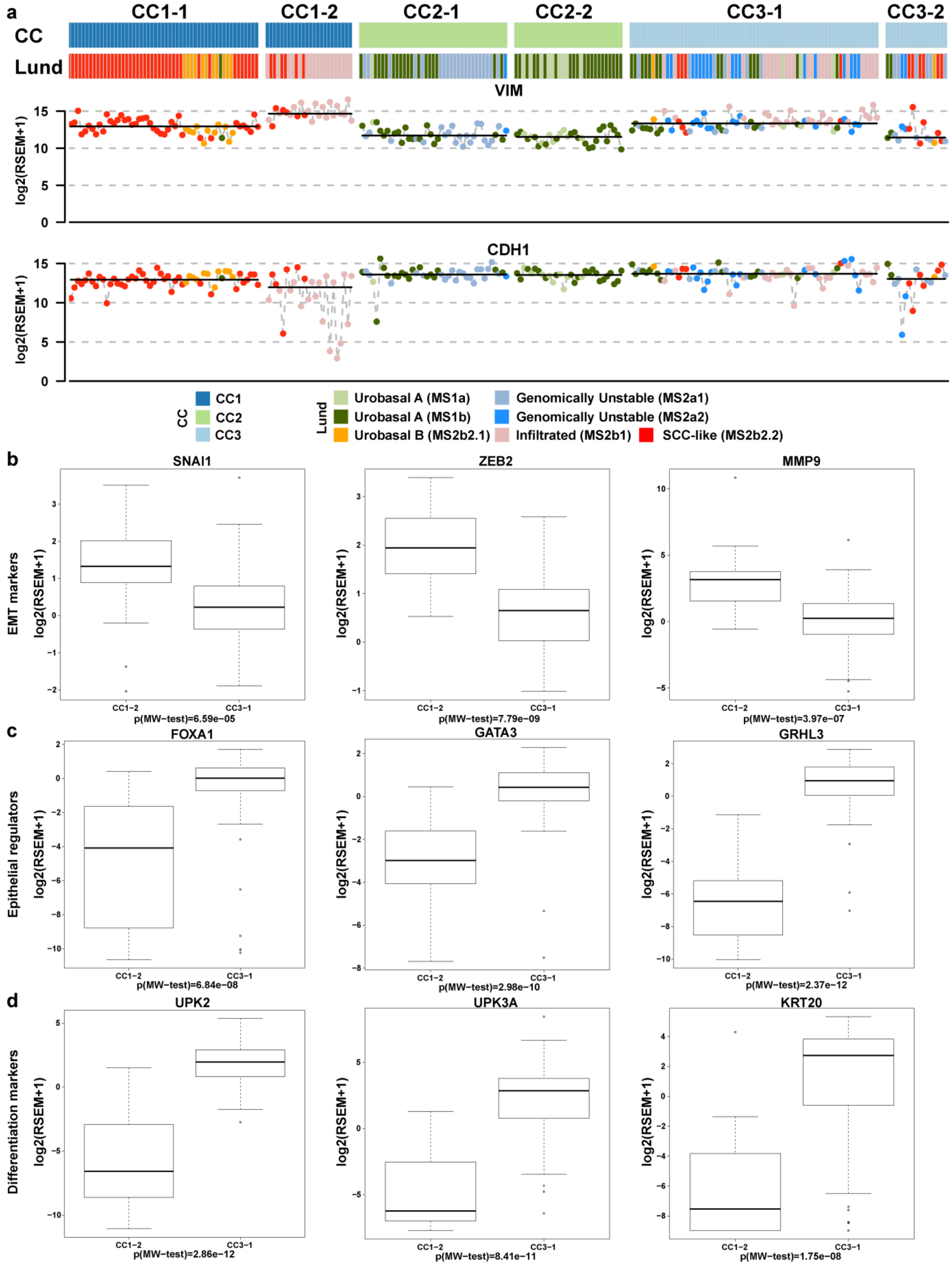

Supplementary Figure 3 - Höglund

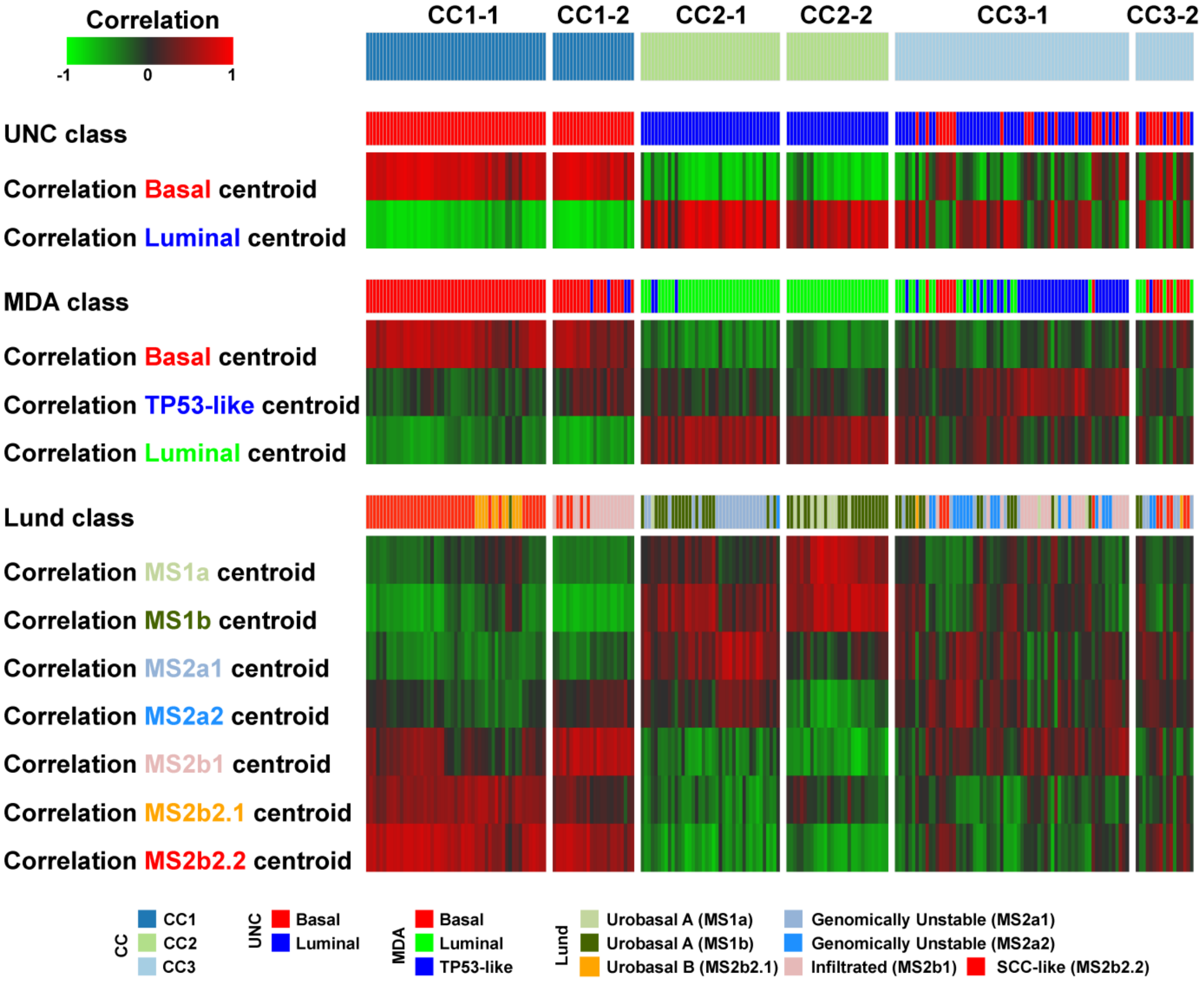

Supplementary table 1
AmiGO analysis (amigo.geneontology.org/rte) output for differentially expressed genes between CC1 and CC2, table derived using PANTHER (go.pantherdb.org)

Significant GO-terms for CC1 upregulated- (N=2886) and downregulated (N=3233) genes, output truncated at p(boferroni)<0.05 or top 25 GO-terms

Analysis Type: PANTHER Overrepresentation Test (release 20141219)
Annotation Version and Release Date: GO Ontology database Released 2015-02-17
Reference List: Homo sapiens (all genes in database)
Bonferroni correction: TRUE

CC1 upregulated genes

GO biological process complete	Enrichment/Depletion	p(bonferroni)
immune system process	+	7.48E-117
biological_process	+	5.99E-105
single-organism process	+	1.32E-97
response to stimulus	+	5.31E-96
immune response	+	2.15E-93
single-organism cellular process	+	9.64E-92
defense response	+	1.95E-89
response to stress	+	1.14E-82
biological regulation	+	1.12E-75
regulation of immune system process	+	2.44E-73
regulation of response to stimulus	+	7.02E-73
positive regulation of biological process	+	5.98E-69
regulation of biological process	+	1.27E-65
positive regulation of response to stimulus	+	1.31E-61
cellular response to stimulus	+	2.14E-60
positive regulation of immune system process	+	1.71E-59
regulation of cellular process	+	3.37E-59
cellular process	+	4.65E-57
signal transduction	+	5.87E-57
single organism signaling	+	1.34E-56
signaling	+	1.34E-56
cell communication	+	4.22E-56
response to external stimulus	+	1.36E-55
positive regulation of cellular process	+	1.93E-51
biological adhesion	+	5.29E-51

CC1 downregulated genes

GO biological process complete	Enrichment/Depletion	p(bonferroni)
biological_process	+	2.11E-33
cellular process	+	2.83E-18
biological regulation	+	9.50E-15
transcription, DNA-templated	+	9.35E-12
nucleic acid-templated transcription	+	9.97E-12
lipid metabolic process	+	1.88E-11
regulation of biological process	+	1.49E-10
detection of chemical stimulus involved in sensory perception of smell	-	3.27E-10
regulation of transcription, DNA-templated	+	3.49E-10
regulation of nucleic acid-templated transcription	+	8.93E-10
regulation of cellular process	+	9.35E-10
translation	-	1.11E-09
regulation of RNA biosynthetic process	+	2.25E-09
biosynthetic process	+	2.29E-09
RNA biosynthetic process	+	3.37E-09
cellular lipid metabolic process	+	3.97E-09
regulation of RNA metabolic process	+	5.51E-09
organic substance biosynthetic process	+	5.55E-09
cellular macromolecule catabolic process	-	5.99E-09
small molecule metabolic process	+	2.16E-08
sensory perception of smell	-	2.38E-08
detection of chemical stimulus involved in sensory perception	-	2.49E-08
xenobiotic metabolic process	+	3.29E-08
cellular response to xenobiotic stimulus	+	4.15E-08
single-organism process	+	4.50E-08

Supplementary table 2

AmiGO analysis (amigo.geneontology.org/rte) output for genes upregulated in CC3-1 vs CC1-2, table derived using PANTHER (go.pantherdb.org)

Significant GO-terms for CC3-1 upregulated genes (N=1984), output truncated at p(boferroni)<0.05 or top 25 GO-terms

Analysis Type: PANTHER Overrepresentation Test (release 20141219)
Annotation Version and Release Date: GO Ontology database Released 2015-02-17
Reference List: Homo sapiens (all genes in database)
Bonferroni correction: TRUE

GO biological process complete	Enrichment/Depletion	p(bonferroni)
biological_process	+	4.95E-24
lipid metabolic process	+	5.86E-19
cellular lipid metabolic process	+	2.14E-14
cellular process	+	8.22E-11
organic hydroxy compound metabolic process	+	1.60E-09
small molecule metabolic process	+	1.56E-08
fatty acid metabolic process	+	1.70E-08
biosynthetic process	+	3.51E-08
lipid biosynthetic process	+	5.20E-08
carboxylic acid metabolic process	+	6.16E-08
single-organism process	+	1.46E-07
oxoacid metabolic process	+	1.66E-07
organic acid metabolic process	+	1.71E-07
organic substance biosynthetic process	+	1.87E-07
monocarboxylic acid metabolic process	+	1.94E-07
metabolic process	+	1.78E-06
epithelial cell differentiation	+	2.29E-06
organic acid catabolic process	+	3.57E-06
carboxylic acid catabolic process	+	3.57E-06
cellular biosynthetic process	+	4.66E-06
organic substance metabolic process	+	1.04E-05
biological regulation	+	1.11E-05
primary metabolic process	+	1.24E-05
small molecule catabolic process	+	1.36E-05
monocarboxylic acid catabolic process	+	1.42E-05

Supplementary table 3
AmiGO analysis (amigo.geneontology.org/rte) output for genes upregulated in CC3-2 vs rest, table derived using PANTHER (go.pantherdb.org)

Significant GO-terms for CC3-2 upregulated genes (N=289), output truncated at p(boferroni)<0.05 or top 25 GO-terms

Analysis Type: PANTHER Overrepresentation Test (release 20141219)
Annotation Version and Release Date: GO Ontology database Released 2015-02-17
Reference List: Homo sapiens (all genes in database)
Bonferroni correction: TRUE

GO biological process complete	Enrichment/Depletion	p(bonferroni)
cell cycle	+	6.50E-12
mitotic cell cycle	+	2.89E-10
mitotic cell cycle process	+	7.00E-09
cell cycle process	+	9.19E-08
single-organism process	+	1.95E-07
organelle fission	+	5.11E-07
cellular process	+	5.64E-07
biological_process	+	6.73E-07
nuclear division	+	8.19E-07
single-organism cellular process	+	5.10E-06
DNA metabolic process	+	1.31E-05
mitotic nuclear division	+	2.75E-05
organelle organization	+	6.73E-05
biological regulation	+	3.50E-04
single-organism organelle organization	+	3.73E-04
chromosome organization	+	6.32E-04
regulation of biological process	+	8.57E-04
cell division	+	9.95E-04
regulation of cellular process	+	1.03E-03
chromosome segregation	+	1.13E-03
nucleic acid metabolic process	+	1.27E-03
nucleobase-containing compound metabolic process	+	1.82E-03
cellular component organization	+	1.88E-03
anatomical structure development	+	3.17E-03
meiotic cell cycle	+	4.56E-03

Supplementary table 4

AmiGO analysis (amigo.geneontology.org/rte) output for genes upregulated in CC2-2 vs rest, table derived using PANTHER (go.pantherdb.org)

Significant GO-terms for CC2-2 upregulated- (N=511) and downregulated (N=1030) genes, output truncated at p(boferroni)<0.05 or top 25 GO-terms

Analysis Type: PANTHER Overrepresentation Test (release 20141219)
Annotation Version and Release Date: GO Ontology database Released 2015-02-17
Reference List: Homo sapiens (all genes in database)
Bonferroni correction: TRUE

CC2-2 upregulated genes

GO biological process complete	Enrichment/Depletion	p(bonferroni)
SRP-dependent cotranslational protein targeting to membrane	+	3.83E-29
cotranslational protein targeting to membrane	+	7.37E-29
protein targeting to ER	+	1.02E-28
establishment of protein localization to endoplasmic reticulum	+	3.59E-28
viral transcription	+	2.51E-27
nuclear-transcribed mRNA catabolic process, nonsense-mediated decay	+	1.13E-26
protein localization to endoplasmic reticulum	+	4.70E-26
viral gene expression	+	4.79E-26
translational termination	+	2.03E-25
multi-organism metabolic process	+	2.49E-25
cellular protein complex disassembly	+	5.02E-24
protein targeting to membrane	+	3.84E-23
translational elongation	+	5.38E-23
protein complex disassembly	+	1.55E-22
macromolecular complex disassembly	+	5.96E-22
nuclear-transcribed mRNA catabolic process	+	3.20E-21
RNA catabolic process	+	1.33E-20
viral life cycle	+	1.52E-20
mRNA catabolic process	+	1.97E-20
translational initiation	+	2.97E-20
establishment of protein localization to membrane	+	2.55E-19
aromatic compound catabolic process	+	5.64E-17
nucleobase-containing compound catabolic process	+	1.39E-16
organic cyclic compound catabolic process	+	2.16E-16
cellular nitrogen compound catabolic process	+	2.80E-16

CC2-2 downregulated genes

GO biological process complete	Enrichment/Depletion	p(bonferroni)
single-organism process	+	4.40E-43
biological_process	+	1.15E-40
single-organism cellular process	+	9.97E-40
mitotic cell cycle	+	4.73E-33
cellular process	+	1.05E-31
cell cycle	+	6.22E-31
cell cycle process	+	1.28E-27
mitotic cell cycle process	+	6.73E-27
biological phase	+	1.84E-26
mitotic cell cycle phase	+	4.84E-26
cell cycle phase	+	4.84E-26
nuclear division	+	4.01E-22
organelle fission	+	3.45E-21
biological regulation	+	6.92E-21
response to stress	+	1.22E-19
cellular component organization	+	2.23E-19
response to stimulus	+	1.32E-18
mitotic nuclear division	+	3.01E-18
cellular component organization or biogenesis	+	6.95E-18
cellular response to stimulus	+	7.54E-18
mitotic prometaphase	+	2.25E-17
regulation of cellular process	+	4.64E-17
regulation of biological process	+	7.67E-17
cell division	+	3.83E-16
positive regulation of biological process	+	9.45E-16

Supplementary table 5
Files downloaded through the TCGA public ftp-site available at "https://tcga-data.nci.nih.gov/tcgafiles/ftp_auth/distro_ftpusers/anonymous/tumor/blca/"

TCGA sample clusters file according to TCGA, Nature, Volume 507 Number 7492, 315-322, Mar. 20, 2014 [doi:10.1038]
BLCA_cluster-assign-k4.tsv

TCGA somatic variant annotation file
PR_TCGA_BLCA_PAIR_Capture_All_Pairs_QCPASS_v5.aggregated.capture.tcga.uuid.automated.somatic.maf

TCGA gene expression files
unc.edu.09898301-3dac-4b86-af1b-6cbad9f2d380.1182808.rsem.isoforms.normalized_results
unc.edu.8038ffb4-38d5-437a-9457-4943c0202a8a.2307668.rsem.isoforms.normalized_results
unc.edu.ac9729e9-6565-4e11-8828-ff43b752a5b6.2097421.rsem.isoforms.normalized_results
unc.edu.370c2202-46b7-4721-a1db-ee80f6ca7782.2116096.rsem.isoforms.normalized_results
unc.edu.4c4fbc19-727b-48b0-82e0-42ca8309f315.1384825.rsem.isoforms.normalized_results
unc.edu.c07c2f21-21d4-49cb-93bb-8d128966fcd6.1351737.rsem.isoforms.normalized_results
unc.edu.d7c8fab5-a3c9-4650-9935-56c0fe27cbe6.1468997.rsem.isoforms.normalized_results
unc.edu.bd49f808-37de-43a4-8a5f-7a36b9661a16.1987611.rsem.isoforms.normalized_results
unc.edu.57baff29-25d2-46ce-937f-f25343dcebc3.2031731.rsem.isoforms.normalized_results
unc.edu.169e537f-9ffc-4d56-9e77-1f38951ee3d3.1475175.rsem.isoforms.normalized_results
unc.edu.18bb3804-de1e-4988-abbf-89c36d07cc01.1966968.rsem.isoforms.normalized_results
unc.edu.cfb10485-b097-4470-bd7e-5ed2abd2e8ac.1181243.rsem.isoforms.normalized_results
unc.edu.a83e6d94-8bef-4ffe-889a-2eecc4b97cae.2120613.rsem.isoforms.normalized_results
unc.edu.c270c34c-0cfd-410e-ab3c-231033196d25.1113454.rsem.isoforms.normalized_results
unc.edu.341d1383-93bd-42d7-80ad-5dba732c1925.2116249.rsem.isoforms.normalized_results
unc.edu.842484b0-6661-4dc2-bf70-b1216ab05475.2316269.rsem.isoforms.normalized_results
unc.edu.9ad7733f-a2a6-4985-8ed3-d2c539eeab96.1474832.rsem.isoforms.normalized_results
unc.edu.a45e5587-488f-4348-a724-fe64e0c5be7d.1351225.rsem.isoforms.normalized_results
unc.edu.d68e2da7-7bd4-4510-8751-02e9f16ad4e6.2095487.rsem.isoforms.normalized_results
unc.edu.fa74ebb2-007d-407a-ba07-12976b75056d.2306747.rsem.isoforms.normalized_results
unc.edu.1a1e861c-14bd-45ea-904a-b14d4059ac95.1350519.rsem.isoforms.normalized_results
unc.edu.c8fd7f63-e309-4a7e-876d-7ca2cf954abe.2309041.rsem.isoforms.normalized_results
unc.edu.32d604cb-3bf5-427c-b60d-3c9cfeb7f0a5.1352036.rsem.isoforms.normalized_results
unc.edu.958520af-f270-49ab-8fb6-57e1ae8ae68f.1179858.rsem.isoforms.normalized_results
unc.edu.20da407e-268c-4a10-9d1f-751974e09067.2089887.rsem.isoforms.normalized_results
unc.edu.f6660fcd-6277-4eb7-8135-5aea56fedd7c.2306172.rsem.isoforms.normalized_results
unc.edu.fac8f7fd-3e80-410a-a281-2b564862b335.1227036.rsem.isoforms.normalized_results
unc.edu.74cbc344-560b-439b-bd14-1542ce579967.2158457.rsem.isoforms.normalized_results
unc.edu.968143b4-2bc1-4c77-88e7-a907a7cd018c.1213298.rsem.isoforms.normalized_results
unc.edu.511d6289-1558-4e90-a77a-05298e15b17c.1390766.rsem.isoforms.normalized_results
unc.edu.df755d45-141e-40d1-96c8-6bfe29c17edc.1351625.rsem.isoforms.normalized_results
unc.edu.b3797921-4b9a-43c4-8904-6fb80fc80c08.1214096.rsem.isoforms.normalized_results
unc.edu.a09bfd63-c31c-461a-b5a4-60670f421233.1352353.rsem.isoforms.normalized_results
unc.edu.1afbe0f3-e320-4d74-8648-0c26ddbeab7f.1351296.rsem.isoforms.normalized_results
unc.edu.575f9b1e-4952-488a-bbdd-ac98c1f68382.1388352.rsem.isoforms.normalized_results
unc.edu.fd79008e-fae5-4276-bc25-eae14eb7b7e4.1112024.rsem.isoforms.normalized_results
unc.edu.7206d218-0e58-4722-8566-cbcbd8b8b4e0.1112177.rsem.isoforms.normalized_results
unc.edu.e7925508-8b2f-4c39-bd7a-033e78b1f875.1387987.rsem.isoforms.normalized_results
unc.edu.3208f694-4779-4548-8cb4-74df829b3345.1474555.rsem.isoforms.normalized_results
unc.edu.3d1b5ffe-38d6-4073-9020-c6897727341c.1113517.rsem.isoforms.normalized_results
unc.edu.1cc49a56-ee39-4fd1-bf80-a6b71695c1f1.1228295.rsem.isoforms.normalized_results
unc.edu.80e9793f-4e30-45ad-8660-3686685f2db9.1227801.rsem.isoforms.normalized_results
unc.edu.4000adba-115e-40bf-afbb-35ad20c4e416.1217948.rsem.isoforms.normalized_results
unc.edu.f89742f4-3575-4dd4-860f-ea9ab0321a48.1821708.rsem.isoforms.normalized_results
unc.edu.edde0611-3cdb-4f1c-a259-68cb4a905bea.1782310.rsem.isoforms.normalized_results
unc.edu.5968c8d3-5f1a-4eb8-8d9b-8bd3fd3fd9f1.1468903.rsem.isoforms.normalized_results
unc.edu.159e6f18-943e-4e7b-af13-da273c52dc16.2157534.rsem.isoforms.normalized_results
unc.edu.325c6503-12a1-4985-99a0-b968850f9d92.1211517.rsem.isoforms.normalized_results
unc.edu.10e3d6b9-8f72-4902-94ca-f89811b98e35.1781760.rsem.isoforms.normalized_results
unc.edu.90c55ddf-3ac3-4599-80f6-e29e486d2070.1966528.rsem.isoforms.normalized_results
unc.edu.024bc00e-0419-4dc6-8df4-ad3af476314d.1475265.rsem.isoforms.normalized_results
unc.edu.06d4cd28-11fb-4f25-8ae0-3fa8e3f3e325.1989602.rsem.isoforms.normalized_results
unc.edu.3167baad-cb92-49e6-8a7d-c59f81709a52.2222238.rsem.isoforms.normalized_results
unc.edu.538ad820-310d-4463-83a2-44ce1cac920c.2115724.rsem.isoforms.normalized_results
unc.edu.d6280a13-a376-4ede-8aab-7a7633cb3b9e.2233431.rsem.isoforms.normalized_results
unc.edu.20ca7c4d-7ef0-4fc5-907b-a90fec12a561.1469257.rsem.isoforms.normalized_results
unc.edu.36f98bd7-f044-4497-9d19-e25c8e847def.1821818.rsem.isoforms.normalized_results
unc.edu.bd1ba628-0cb5-4103-baa4-3f430d24d359.1474894.rsem.isoforms.normalized_results
unc.edu.652f8ab9-33b6-4e10-a84e-1e1b1ef0b707.1326775.rsem.isoforms.normalized_results
unc.edu.94309b2e-c58c-4e2b-981d-0badb3872db7.2032635.rsem.isoforms.normalized_results
unc.edu.2f3c17f6-be91-4195-9aa7-e02298d118f3.1180727.rsem.isoforms.normalized_results
unc.edu.3bea01e7-e5bc-484b-91f4-a143caed0aed.1475070.rsem.isoforms.normalized_results
unc.edu.36a95ee7-1822-4d07-8110-843792006625.1180018.rsem.isoforms.normalized_results
unc.edu.d3cf6983-3dfc-4533-8809-605c197fc24e.2089346.rsem.isoforms.normalized_results
unc.edu.7668e1a3-cf31-411a-8bce-aff592a0f053.1927204.rsem.isoforms.normalized_results
unc.edu.b9f76fc8-ca33-46c5-89a0-ae16d42f987f.1114904.rsem.isoforms.normalized_results
unc.edu.a56ef438-c2ac-4c6f-9283-59e38ba14f28.1966591.rsem.isoforms.normalized_results
unc.edu.c32574a0-5f85-4053-bb98-8472b496975e.1214631.rsem.isoforms.normalized_results
unc.edu.69b30673-9ec0-4c05-b75c-12bcb1f7268f.2308016.rsem.isoforms.normalized_results
unc.edu.b4bb6509-7d67-4d5e-8fd1-624fa373df4d.1474749.rsem.isoforms.normalized_results
unc.edu.1b08c8c9-5739-409d-9cbc-3b06ad1f60e6.1215497.rsem.isoforms.normalized_results
unc.edu.f873fe6d-9733-48a9-b7e0-23dc50874fbc.2157834.rsem.isoforms.normalized_results

unc.edu.61b0add8-ad93-4f2a-a95b-fa257934962f.1468793.rsem.isoforms.normalized_results
unc.edu.6621636c-4a29-4ad3-99bc-406b1820d063.2089043.rsem.isoforms.normalized_results
unc.edu.ad775597-406f-4470-894a-e37d0666761b.1248454.rsem.isoforms.normalized_results
unc.edu.221bd562-e77d-4374-9491-efb5c3b026c3.1783495.rsem.isoforms.normalized_results
unc.edu.e356927b-b100-4adf-9935-e4fbf31d1fd3.2315435.rsem.isoforms.normalized_results
unc.edu.d1b0c4db-59b7-47ac-b26f-fb14c5aac200.2097134.rsem.isoforms.normalized_results
unc.edu.21c8f741-963f-463e-ac5b-1c2c1aa9d8cb.1989368.rsem.isoforms.normalized_results
unc.edu.433eed62-f9d3-440a-bc04-f0156c70ee65.2167080.rsem.isoforms.normalized_results
unc.edu.dbffd0f0-d063-45a2-8cdb-e6ffa6c9e66d.1987864.rsem.isoforms.normalized_results
unc.edu.fa1f8758-485c-4148-87b7-af891f652f48.2096404.rsem.isoforms.normalized_results
unc.edu.227c6e50-7801-4fb6-91d3-eda4313024bb.2233605.rsem.isoforms.normalized_results
unc.edu.bd5b7c93-fabe-4bb8-a78f-981aeed75d79.1783807.rsem.isoforms.normalized_results
unc.edu.99471b35-21e6-44cb-863e-d60521a71b31.1966750.rsem.isoforms.normalized_results
unc.edu.12c0b9eb-97fc-4fa1-8f64-49c0e3e171a2.1180376.rsem.isoforms.normalized_results
unc.edu.eb5b988d-308e-4afb-b21b-8bdd45010c72.1225719.rsem.isoforms.normalized_results
unc.edu.70c7f658-5208-4b3f-9767-27a3b06b8ddc.1389051.rsem.isoforms.normalized_results
unc.edu.1002d9de-3bd1-4d22-8f2a-64b3114264e0.2032159.rsem.isoforms.normalized_results
unc.edu.388f02e0-68bb-4be2-9271-60a42d3a3e9d.1327084.rsem.isoforms.normalized_results
unc.edu.535736bf-6e19-4fc6-bf3d-a2fb4739c763.2316495.rsem.isoforms.normalized_results
unc.edu.7d6ea81b-b8a5-4d3b-aa54-9bf51d8b698b.1476019.rsem.isoforms.normalized_results
unc.edu.e68b253d-6149-4c42-a5b3-1ddc2448759b.1784324.rsem.isoforms.normalized_results
unc.edu.81018f98-fee6-4cde-b6f6-3b6254e3e8d7.1928138.rsem.isoforms.normalized_results
unc.edu.dc901511-9e0f-4fc6-b6f4-bee8e627ce25.1469018.rsem.isoforms.normalized_results
unc.edu.4c8aa0aa-c1ae-4aff-96f2-4636bb1d5c47.1969083.rsem.isoforms.normalized_results
unc.edu.73220a3d-e4e3-4494-a832-86baa98333b4.1784001.rsem.isoforms.normalized_results
unc.edu.a66ddf41-6af9-47c0-952b-688aac83c8c8.2097240.rsem.isoforms.normalized_results
unc.edu.c40ff5cd-bffa-40c4-a192-9830b0da3daa.1468850.rsem.isoforms.normalized_results
unc.edu.5ddb876d-dd32-4f33-89d1-d95e460e1e60.1469231.rsem.isoforms.normalized_results
unc.edu.eb210789-9eb4-4def-a83d-3f0254b3c6dd.1228931.rsem.isoforms.normalized_results
unc.edu.0aba0f10-05b2-4045-822b-045f61457427.2096018.rsem.isoforms.normalized_results
unc.edu.50fdf7c3-0e77-4013-ae2b-0e7c70b8d3e3.2095428.rsem.isoforms.normalized_results
unc.edu.4c23170b-a5e0-41c8-8309-a5bfb2823491.2308975.rsem.isoforms.normalized_results
unc.edu.2480ce78-21a7-4717-8b08-8176d9fe6834.2306418.rsem.isoforms.normalized_results
unc.edu.2d83d812-8f9b-443b-8643-dd3104b5068e.2306999.rsem.isoforms.normalized_results
unc.edu.01a7643f-d7be-40e8-bdc1-562d825e8ba8.2096835.rsem.isoforms.normalized_results
unc.edu.c130027b-a3f5-49be-b26d-a0416b7904b5.2306458.rsem.isoforms.normalized_results
unc.edu.d74030bf-dfa1-4f65-9596-fd505733139e.1475644.rsem.isoforms.normalized_results
unc.edu.8f7b5e84-55c2-4f98-a6a2-56b862161a98.1475149.rsem.isoforms.normalized_results
unc.edu.fd93cd6f-ab1c-460c-bfca-268c485a4f5b.1384511.rsem.isoforms.normalized_results
unc.edu.2c681223-21a5-4c58-acbb-2d51516bff12.1386366.rsem.isoforms.normalized_results
unc.edu.50dd2bb1-e069-42f8-89f4-1e3055adafee.1782706.rsem.isoforms.normalized_results
unc.edu.5b0896f2-8fd9-42ee-b56d-b140f8d8657e.1113050.rsem.isoforms.normalized_results
unc.edu.b5036d12-d9cc-46a4-b60a-49740310380c.1107947.rsem.isoforms.normalized_results
unc.edu.fc2ec949-9292-4709-b0ea-37368add311d.1821548.rsem.isoforms.normalized_results
unc.edu.f68894de-6db1-472b-bbda-f1c6e60a818b.1927248.rsem.isoforms.normalized_results
unc.edu.d6d7412b-5ec2-413e-a85d-7f765c67afa3.1213748.rsem.isoforms.normalized_results
unc.edu.cbc4cdac-a504-47ed-96cf-5c459f7febd0.1821839.rsem.isoforms.normalized_results
unc.edu.c87fa518-41c5-417a-88ac-0b300f1a8732.2231970.rsem.isoforms.normalized_results
unc.edu.b6ebbe90-feb4-4e8e-9408-031db2297c1c.2157382.rsem.isoforms.normalized_results
unc.edu.a5118740-bf71-47e8-a2e3-506792865c90.2090201.rsem.isoforms.normalized_results
unc.edu.791a46e8-4d45-4878-b09d-a4f64ce88746.1474973.rsem.isoforms.normalized_results
unc.edu.772b8a77-bb90-40a8-ad48-f7a1fc1769de.1347684.rsem.isoforms.normalized_results
unc.edu.58e49daa-eced-4ca8-af92-98af61f8b6f1.2039021.rsem.isoforms.normalized_results
unc.edu.4e0401b2-6ad4-4709-aa43-f2af0afef259.2095196.rsem.isoforms.normalized_results
unc.edu.459468e0-79bc-4544-b168-27a85342d4c1.2236144.rsem.isoforms.normalized_results
unc.edu.0c1e7ecc-d20d-4047-8419-6347537cb8a3.1821630.rsem.isoforms.normalized_results
unc.edu.38e62ae5-729f-41fa-94e7-7e3caa0a246b.1351677.rsem.isoforms.normalized_results
unc.edu.a40c2a50-5627-4fd2-a6a3-2661c4ee5839.1990169.rsem.isoforms.normalized_results
unc.edu.e43f2bcb-9ba5-4b65-9042-4d3b06224c0f.1821649.rsem.isoforms.normalized_results
unc.edu.429f0e9d-c160-4b36-a31b-dab1097c6129.1388311.rsem.isoforms.normalized_results
unc.edu.5eb594ca-ba46-45d2-8532-0fabac564e34.1821986.rsem.isoforms.normalized_results
unc.edu.63fcdb32-0c58-4541-afbc-96b34020c5c8.1351589.rsem.isoforms.normalized_results
unc.edu.83c4827f-742b-42bf-96d0-335b484ee724.1966818.rsem.isoforms.normalized_results
unc.edu.5d19bc28-408e-4dc6-bc4b-95b28fa0dddc.2307467.rsem.isoforms.normalized_results
unc.edu.6ab37abf-8cc9-4274-bf5c-f08ec3f4d136.2090236.rsem.isoforms.normalized_results
unc.edu.febead73-3b12-44e6-8bab-a7cfa7bf1129.1179295.rsem.isoforms.normalized_results
unc.edu.68b08ead-5e38-490f-8654-b3db19c80fca.1475048.rsem.isoforms.normalized_results
unc.edu.c27a2180-0dec-43ec-bf0f-2cb0acaea022.1821762.rsem.isoforms.normalized_results
unc.edu.fee1bb5e-af08-43f5-a313-ebe56ceb6a37.2159042.rsem.isoforms.normalized_results
unc.edu.791bcd93-c949-49b3-aaa4-4c8af82f5ec1.2246338.rsem.isoforms.normalized_results
unc.edu.f7df4f7f-cd8e-44ba-b16f-203d6049c4fa.1782627.rsem.isoforms.normalized_results
unc.edu.4be53c32-b222-44dd-b0b9-6a935591380a.2090034.rsem.isoforms.normalized_results
unc.edu.f6b30464-1e34-4b52-baff-544f5fc8f52f.1782936.rsem.isoforms.normalized_results
unc.edu.f732f86b-5190-4827-8471-656a43b47681.1475015.rsem.isoforms.normalized_results
unc.edu.57eda6ed-26b0-4bf6-98ad-80f4872b9832.1328757.rsem.isoforms.normalized_results
unc.edu.eb59f0f9-3b20-4dce-833e-6a67076ddc01.1356427.rsem.isoforms.normalized_results
unc.edu.2fdb2cad-53e5-44e4-8b80-ee5801964b20.1112811.rsem.isoforms.normalized_results
unc.edu.ad2ee4b7-ec51-4076-bc5b-6601b5abeecb.1475005.rsem.isoforms.normalized_results
unc.edu.c7f36dba-a507-46d3-8e68-7f98b8b645e9.1468920.rsem.isoforms.normalized_results
unc.edu.ed8b8326-fa77-4e72-b146-49c0543a5792.2306565.rsem.isoforms.normalized_results
unc.edu.7773fd96-8ab7-417b-befd-ea63dc596a7a.2032568.rsem.isoforms.normalized_results
unc.edu.5e7e3e20-cbfa-476d-bb92-fa3a58207a9b.1113971.rsem.isoforms.normalized_results

unc.edu.30f1a64e-5df3-4e26-b820-a4254e7e3a7e.2306586.rsem.isoforms.normalized_results
unc.edu.ae3c4446-cb58-4b5b-9eb8-6c81e1bf4535.1474941.rsem.isoforms.normalized_results
unc.edu.fe6853dd-864f-4257-869f-a425aab32346.2256101.rsem.isoforms.normalized_results
unc.edu.3add40ce-6d64-4ded-9ab6-2f076c420dc4.1112696.rsem.isoforms.normalized_results
unc.edu.bf7a27d9-35a8-4c59-bf27-e3fa9b271e6e.1475132.rsem.isoforms.normalized_results
unc.edu.ee7d92c6-8a20-41ec-8e43-7a7c462b13d5.2032904.rsem.isoforms.normalized_results
unc.edu.f1e23b9a-a79f-439c-a4b9-58fbe9899e98.2096608.rsem.isoforms.normalized_results
unc.edu.e33ab42a-bfb3-4923-a305-a6babcee61f4.1350228.rsem.isoforms.normalized_results
unc.edu.088b27e8-38f6-41ff-a861-d371415fd7f0.2233156.rsem.isoforms.normalized_results
unc.edu.6321d37f-3658-447d-96a4-099e8072217a.2306399.rsem.isoforms.normalized_results
unc.edu.d22b6ef3-9b08-44d9-b37f-e7f61ce89404.1113722.rsem.isoforms.normalized_results
unc.edu.dafc2af2-7b91-45a4-ae67-fe22ab060f06.1475167.rsem.isoforms.normalized_results
unc.edu.d692892d-d63b-4ac2-9627-ae255ab17cab.1474926.rsem.isoforms.normalized_results
unc.edu.d785b32a-686a-43d6-9e0b-0b63cfd63772.1212370.rsem.isoforms.normalized_results
unc.edu.ca10d08d-3cc5-4171-9a70-66fdff3c0c3d.1182043.rsem.isoforms.normalized_results
unc.edu.029cb0c8-9a67-4af0-9137-a5fbe08fce20.1474870.rsem.isoforms.normalized_results
unc.edu.c394bf58-ce01-4ad3-a0f7-f2c5a251356d.2116128.rsem.isoforms.normalized_results
unc.edu.518085b3-f993-4346-bb4d-b7244e9435d6.1244669.rsem.isoforms.normalized_results
unc.edu.b68f9935-3265-4930-a221-61bc9f8e2eda.2282065.rsem.isoforms.normalized_results
unc.edu.b6a8b608-8bf6-4d57-a720-bd4aaced4591.1245475.rsem.isoforms.normalized_results
unc.edu.6c392517-fe3d-4d86-8397-c08734fe20cc.1772371.rsem.isoforms.normalized_results
unc.edu.8f1bfe41-e48c-4b5d-8b03-8def7666da02.1966931.rsem.isoforms.normalized_results
unc.edu.a36865ec-a5fa-4277-a887-325373acadb1.1928598.rsem.isoforms.normalized_results
unc.edu.d27207d8-a3cf-475b-963a-e393eef98538.2045790.rsem.isoforms.normalized_results
unc.edu.c11e3246-c33d-47ef-b4b4-be287b92d91e.1111951.rsem.isoforms.normalized_results
unc.edu.c1ce17a6-3e8b-41b7-97bc-9213b352ce84.2233859.rsem.isoforms.normalized_results
unc.edu.b8ecfb28-27d0-4ad4-b73c-cc21be41af31.1226319.rsem.isoforms.normalized_results
unc.edu.f154cc00-e118-4d4c-b9d2-2f1f71e0a964.1373741.rsem.isoforms.normalized_results
unc.edu.6c390573-7472-441d-956b-9f4747a18f2b.1212880.rsem.isoforms.normalized_results
unc.edu.b660dd38-f986-4143-9445-b66cb60f42dd.2166914.rsem.isoforms.normalized_results
unc.edu.10efe18b-413b-4e57-b6e2-ce5cd2dbb839.2176809.rsem.isoforms.normalized_results
unc.edu.1cd3bf53-27a7-4fda-8501-8270da2b8ceb.1927223.rsem.isoforms.normalized_results
unc.edu.91c8d1c3-1afe-4c4c-ae81-7d1f989e0139.1225980.rsem.isoforms.normalized_results
unc.edu.e9acf520-2eb5-4b29-84c8-10ae6c5919e7.1989250.rsem.isoforms.normalized_results
unc.edu.5676c0ee-c460-492c-bf00-73d5ddb1d647.1468866.rsem.isoforms.normalized_results
unc.edu.66132b23-e10a-49d9-89c5-129d7d98c17b.1474640.rsem.isoforms.normalized_results
unc.edu.dfc4aacd-32b1-4cf8-91f9-3c865cf56ec9.1179832.rsem.isoforms.normalized_results
unc.edu.dfffbe9c-c83a-4087-a3f7-2be3d63a2eaa.2254656.rsem.isoforms.normalized_results
unc.edu.ea9d4bd8-1f4e-4ad5-81f7-803b078f2603.1468934.rsem.isoforms.normalized_results
unc.edu.fd7fac82-d8e3-4133-be0e-ec5b2badc5d1.2306303.rsem.isoforms.normalized_results
unc.edu.168e440a-b356-45c7-849c-43823703417e.2032107.rsem.isoforms.normalized_results
unc.edu.22e154de-0e3b-443b-8420-48d68d6c1ce4.1179561.rsem.isoforms.normalized_results
unc.edu.381b51af-d1f7-41db-a69e-49d681b3ef39.1114412.rsem.isoforms.normalized_results
unc.edu.cec64615-07c6-4e49-8e59-afb97d37464f.1112300.rsem.isoforms.normalized_results
unc.edu.72ea067a-e5ae-43f3-97b8-2283b35df8d6.1987767.rsem.isoforms.normalized_results
unc.edu.b1984010-7ef5-4df4-abfc-d18502cf124c.1179032.rsem.isoforms.normalized_results
unc.edu.3c499f07-67e2-4032-a0b5-b9edd90035f7.1179259.rsem.isoforms.normalized_results
unc.edu.7e8935da-773b-4337-8fa2-f52ebbaceaee.2159073.rsem.isoforms.normalized_results
unc.edu.043d2756-1abf-411f-aaac-b448ed088be1.1929154.rsem.isoforms.normalized_results
unc.edu.b290727b-23ed-4e73-9071-cf2c06671430.1469040.rsem.isoforms.normalized_results
unc.edu.f3872d71-1889-4756-bdb4-d5ae383758a8.1385605.rsem.isoforms.normalized_results
unc.edu.037904cf-92c3-443c-8800-0156db1e3a89.1112964.rsem.isoforms.normalized_results
unc.edu.8c79f5ec-ce4f-4c86-8d1c-71495c276d74.1798459.rsem.isoforms.normalized_results
unc.edu.0fa20811-219b-4d76-a8f6-d838e4de093e.1929317.rsem.isoforms.normalized_results
unc.edu.d31897c3-5d0a-4104-bdc6-4b12eb061c2a.2316197.rsem.isoforms.normalized_results
unc.edu.6491f391-9704-4249-bc81-d712e755ec74.1110655.rsem.isoforms.normalized_results
unc.edu.bc993392-5f2f-4cb8-8d27-26f3df2b3583.1214137.rsem.isoforms.normalized_results
unc.edu.3833acbb-2ea2-4442-90af-6b009ae2543b.2235558.rsem.isoforms.normalized_results
unc.edu.b8afe780-f680-40db-a941-e984660ff90c.1469133.rsem.isoforms.normalized_results
unc.edu.42db28fc-4d5f-4b0f-aa86-7d27197c7649.1386234.rsem.isoforms.normalized_results
unc.edu.3cefcb0b-796f-4ef0-b50d-a6ed17e9be4d.1179777.rsem.isoforms.normalized_results
unc.edu.94447759-c20c-49f5-b51f-cbfdea41f2e8.1121022.rsem.isoforms.normalized_results
unc.edu.58382a39-eb95-4cd0-ad04-48c6da4b4a56.1348868.rsem.isoforms.normalized_results
unc.edu.bb0c2732-5fb4-44e7-bc62-a645cf1c1a56.1385181.rsem.isoforms.normalized_results
unc.edu.41836f0e-f807-4afa-ae39-c0eb16fa6b54.1474957.rsem.isoforms.normalized_results
unc.edu.4a0b6e3c-a167-4ca6-876b-6fb82fa8fdb6.2115956.rsem.isoforms.normalized_results
unc.edu.e71db8e9-8ee2-46dc-935a-3274f1fab863.2234031.rsem.isoforms.normalized_results
unc.edu.6aec2686-fcea-4d3e-8311-0b6ccad4c000.1469245.rsem.isoforms.normalized_results
unc.edu.6f3cb879-cf6d-4347-8aae-b6a6ee7a4274.1349325.rsem.isoforms.normalized_results
unc.edu.b7290db7-112b-494e-80f7-bb05860f6c7e.2158295.rsem.isoforms.normalized_results
unc.edu.c4fbd136-4227-4792-ac53-8c887aa6ab1a.1474622.rsem.isoforms.normalized_results
unc.edu.80cdd385-2a6a-4d59-81c7-f36786637323.1179666.rsem.isoforms.normalized_results
unc.edu.92e94724-a03e-4b58-91b2-f309ab8dc943.2157870.rsem.isoforms.normalized_results
unc.edu.9a7e2ecf-3470-427e-93cd-8461a3a3a5b8.1385817.rsem.isoforms.normalized_results
unc.edu.8f482c29-37fe-400f-b178-224765a0c037.1841691.rsem.isoforms.normalized_results
unc.edu.312ea080-3831-453d-aa8c-e466192c8309.1247529.rsem.isoforms.normalized_results
unc.edu.3458d1fe-d159-4522-a627-70c5cc180daa.1228164.rsem.isoforms.normalized_results
unc.edu.2bdc311e-59cc-449f-b8dc-6662052678fd.1179061.rsem.isoforms.normalized_results
unc.edu.80b2573f-d8e0-4d0d-84a6-301c5103267d.1469202.rsem.isoforms.normalized_results
unc.edu.80ddbbc6-11ff-469a-96ec-a72e0e2ec9c4.1475105.rsem.isoforms.normalized_results

TCGA SNP6 copy number files

SNOWS_p_TCGA_Batch_309_310_NSP_GenomeWideSNP_6_A06_1362050
WHIPS_p_1TCGA_220_235_N_GenomeWideSNP_6_E11_1230920
WHIPS_p_1TCGA_220_235_N_GenomeWideSNP_6_H01_1230950
HORNS_p_TCGA_b110_113_SNP_N_GenomeWideSNP_6_A09_772252
WAXEN_p_TCGA_252_253_N_GenomeWideSNP_6_B06_1270710
ENDUE_p_TCGA_271_76_79_280_81_N_GenomeWideSNP_6_D04_1346856
FRUIT_p_TCGAb_327_328_329_NSP_GenomeWideSNP_6_D10_1367932
FOXED_p_TCGA_b122_128_SNP_N_GenomeWideSNP_6_F03_781824
SNOWS_p_TCGA_Batch_309_310_NSP_GenomeWideSNP_6_B04_1362052
WAXEN_p_TCGA_252_253_N_GenomeWideSNP_6_A12_1270610
WAXEN_p_TCGA_252_253_N_GenomeWideSNP_6_B10_1270600
CODON_p_TCGA_293_294_295_N_GenomeWideSNP_6_H01_1343650
AVISO_p_4TCGA_242_238_250_mN_GenomeWideSNP_6_A11_1231488
CONGA_p_TCGA_b_317_318_319_NSP_GenomeWideSNP_6_C02_1365210
AMAZE_p_TCGASNP_b86_87_88_N_GenomeWideSNP_6_H02_735486
CONGA_p_TCGA_b_317_318_319_NSP_GenomeWideSNP_6_A10_1365246
CODON_p_TCGA_293_294_295_N_GenomeWideSNP_6_E09_1343612
WHIPS_p_1TCGA_220_235_N_GenomeWideSNP_6_F05_1230986
ENDUE_p_TCGA_271_76_79_280_81_N_GenomeWideSNP_6_B04_1346730
OKRAS_p_TCGA_198_199_200_SNP_N_GenomeWideSNP_6_F11_955304
OKRAS_p_TCGA_198_199_200_SNP_N_GenomeWideSNP_6_G10_955454
TYPED_p_TCGA_188_192_Mirn_SNP_N_GenomeWideSNP_6_B08_913832
TYPED_p_TCGA_188_192_Mirn_SNP_N_GenomeWideSNP_6_B09_913896
WAXEN_p_TCGA_252_253_N_GenomeWideSNP_6_C06_1270620
LOLLS_p_TCGASNP_223_225_N_GenomeWideSNP_6_H07_1152008
CODON_p_TCGA_293_294_295_N_GenomeWideSNP_6_E02_1343674
ENDUE_p_TCGA_271_76_79_280_81_N_GenomeWideSNP_6_D03_1346864
AVISO_p_4TCGA_242_238_250_mN_GenomeWideSNP_6_A04_1231534
FRUIT_p_TCGAb_327_328_329_NSP_GenomeWideSNP_6_E04_1367920
FOXED_p_TCGA_b122_128_SNP_N_GenomeWideSNP_6_G03_781782
COPAL_p_TCGAb_337_338_339_NSP_GenomeWideSNP_6_A09_1373638
AMAZE_p_TCGASNP_b86_87_88_N_GenomeWideSNP_6_D02_735530
WHIRR_p_TCGA_168_169_170_redo_N_GenomeWideSNP_6_A08_845278
COPAL_p_TCGAb_337_338_339_NSP_GenomeWideSNP_6_A11_1373690
AMAZE_p_TCGASNP_b86_87_88_N_GenomeWideSNP_6_C09_735556
AMAZE_p_TCGASNP_b86_87_88_N_GenomeWideSNP_6_E09_735456
COPAL_p_TCGAb_337_338_339_NSP_GenomeWideSNP_6_B08_1373704
BISON_p_TCGA_164_173_175_SNP_N_GenomeWideSNP_6_F08_863276
CONGA_p_TCGA_b_317_318_319_NSP_GenomeWideSNP_6_B12_1365186
WHIPS_p_1TCGA_220_235_N_GenomeWideSNP_6_F07_1230886
WHIRR_p_TCGA_168_169_170_redo_N_GenomeWideSNP_6_C05_845156
OKRAS_p_TCGA_198_199_200_SNP_N_GenomeWideSNP_6_G01_955444
SNOWS_p_TCGA_Batch_309_310_NSP_GenomeWideSNP_6_C02_1362012
BISON_p_TCGA_164_173_175_SNP_N_GenomeWideSNP_6_H02_863238
FOXED_p_TCGA_b122_128_SNP_N_GenomeWideSNP_6_G04_781802
WAXEN_p_TCGA_252_253_N_GenomeWideSNP_6_B05_1270718
SNOWS_p_TCGA_Batch_309_310_NSP_GenomeWideSNP_6_C04_1362056
HORNS_p_TCGA_b110_113_SNP_N_GenomeWideSNP_6_A04_772348
FRUIT_p_TCGAb_327_328_329_NSP_GenomeWideSNP_6_E10_1367966
OKRAS_p_TCGA_198_199_200_SNP_N_GenomeWideSNP_6_D02_955420
OKRAS_p_TCGA_198_199_200_SNP_N_GenomeWideSNP_6_C10_955466
HORNS_p_TCGA_b110_113_SNP_N_GenomeWideSNP_6_A02_772344
WHIPS_p_1TCGA_220_235_N_GenomeWideSNP_6_G07_1230992
AMAZE_p_TCGASNP_b86_87_88_N_GenomeWideSNP_6_C11_735468
FOXED_p_TCGA_b122_128_SNP_N_GenomeWideSNP_6_F11_781866
AMAZE_p_TCGASNP_b86_87_88_N_GenomeWideSNP_6_F05_735440
BISON_p_TCGA_164_173_175_SNP_N_GenomeWideSNP_6_F09_863298
WHIPS_p_1TCGA_220_235_N_GenomeWideSNP_6_F06_1230930
XYLEM_p_TCGASNP_207_212_N_GenomeWideSNP_6_E04_1051190
GLIAS_p_TCGA_204_205_206_207_N_GenomeWideSNP_6_E10_1051830
DLP_REDO_FROM_CONGA_B03
FRUIT_p_TCGAb_327_328_329_NSP_GenomeWideSNP_6_E06_1367852
SNOWS_p_TCGA_Batch_309_310_NSP_GenomeWideSNP_6_B12_1362068
CODON_p_TCGA_293_294_295_N_GenomeWideSNP_6_F05_1343540
COPAL_p_TCGAb_337_338_339_NSP_GenomeWideSNP_6_B06_1373642
GLIAS_p_TCGA_204_205_206_207_N_GenomeWideSNP_6_E01_1051786
ENDUE_p_TCGA_271_76_79_280_81_N_GenomeWideSNP_6_E05_1346908
SNOWS_p_TCGA_Batch_309_310_NSP_GenomeWideSNP_6_B06_1362010
AVISO_p_4TCGA_242_238_250_mN_GenomeWideSNP_6_A12_1231568
SNOWS_p_TCGA_Batch_309_310_NSP_GenomeWideSNP_6_B10_1362078
XYLEM_p_TCGASNP_207_212_N_GenomeWideSNP_6_B08_1051166
BISON_p_TCGA_164_173_175_SNP_N_GenomeWideSNP_6_C11_863354
COPAL_p_TCGAb_337_338_339_NSP_GenomeWideSNP_6_C12_1373596
SNOWS_p_TCGA_Batch_309_310_NSP_GenomeWideSNP_6_C09_1362008
LOLLS_p_TCGASNP_223_225_N_GenomeWideSNP_6_H04_1151988
XYLEM_p_TCGASNP_207_212_N_GenomeWideSNP_6_E05_1051216
CODON_p_TCGA_293_294_295_N_GenomeWideSNP_6_G07_1343692
OKRAS_p_TCGA_198_199_200_SNP_N_GenomeWideSNP_6_D01_955342
OKRAS_p_TCGA_198_199_200_SNP_N_GenomeWideSNP_6_D07_955430
CODON_p_TCGA_293_294_295_N_GenomeWideSNP_6_D05_1343632
BASIC_p_TCGASNP_219_221_223_N_GenomeWideSNP_6_D02_1148644
HORNS_p_TCGA_b110_113_SNP_N_GenomeWideSNP_6_C04_772308

COPAL_p_TCGAb_337_338_339_NSP_GenomeWideSNP_6_A03_1373684
LOLLS_p_TCGASNP_223_225_N_GenomeWideSNP_6_B10_1151972
HORNS_p_TCGA_b110_113_SNP_N_GenomeWideSNP_6_B12_772400
WAXEN_p_TCGA_252_253_N_GenomeWideSNP_6_B09_1270730
LOLLS_p_TCGASNP_223_225_N_GenomeWideSNP_6_F04_1152004
COPAL_p_TCGAb_337_338_339_NSP_GenomeWideSNP_6_B01_1373628
OKRAS_p_TCGA_198_199_200_SNP_N_GenomeWideSNP_6_A11_955478
HORNS_p_TCGA_b110_113_SNP_N_GenomeWideSNP_6_D10_772298
OKRAS_p_TCGA_198_199_200_SNP_N_GenomeWideSNP_6_F05_955438
WHIPS_p_1TCGA_220_235_N_GenomeWideSNP_6_G01_1231006
CODON_p_TCGA_293_294_295_N_GenomeWideSNP_6_E10_1343560
WHIPS_p_1TCGA_220_235_N_GenomeWideSNP_6_E03_1231040
COPAL_p_TCGAb_337_338_339_NSP_GenomeWideSNP_6_C06_1373730
DLP_REDO_FROM_CODON_E01
WHIPS_p_1TCGA_220_235_N_GenomeWideSNP_6_D11_1230932
WHIRR_p_TCGA_168_169_170_redo_N_GenomeWideSNP_6_A07_845096
AVISO_p_4TCGA_242_238_250_mN_GenomeWideSNP_6_A08_1231504
OKRAS_p_TCGA_198_199_200_SNP_N_GenomeWideSNP_6_H03_955388
XYLEM_p_TCGASNP_207_212_N_GenomeWideSNP_6_G06_1051242
HORNS_p_TCGA_b110_113_SNP_N_GenomeWideSNP_6_B10_772410
ENDUE_p_TCGA_271_76_79_280_81_N_GenomeWideSNP_6_E04_1346828
AVISO_p_4TCGA_242_238_250_mN_GenomeWideSNP_6_A06_1231506
CONGA_p_TCGA_b_317_318_319_NSP_GenomeWideSNP_6_B10_1365172
WAXEN_p_TCGA_252_253_N_GenomeWideSNP_6_B01_1270602
OKRAS_p_TCGA_198_199_200_SNP_N_GenomeWideSNP_6_A09_955332
CODON_p_TCGA_293_294_295_N_GenomeWideSNP_6_D06_1343660
WHIPS_p_1TCGA_220_235_N_GenomeWideSNP_6_H06_1230924
FRUIT_p_TCGAb_327_328_329_NSP_GenomeWideSNP_6_D04_1367994
WHIPS_p_1TCGA_220_235_N_GenomeWideSNP_6_E05_1231008
CONGA_p_TCGA_b_317_318_319_NSP_GenomeWideSNP_6_B08_1365270
COPAL_p_TCGAb_337_338_339_NSP_GenomeWideSNP_6_B12_1373736
LOLLS_p_TCGASNP_223_225_N_GenomeWideSNP_6_C07_1152044
FOXED_p_TCGA_b122_128_SNP_N_GenomeWideSNP_6_F08_781776
RUNIC_p_2TCGA235_237_250_242_mN_GenomeWideSNP_6_A01_1231086
TYPED_p_TCGA_188_192_Mirn_SNP_N_GenomeWideSNP_6_D04_913826
WAXEN_p_TCGA_252_253_N_GenomeWideSNP_6_A08_1270664
ENDUE_p_TCGA_271_76_79_280_81_N_GenomeWideSNP_6_G04_1346752
COPAL_p_TCGAb_337_338_339_NSP_GenomeWideSNP_6_B10_1373668
AVISO_p_4TCGA_242_238_250_mN_GenomeWideSNP_6_A10_1231472
ENDUE_p_TCGA_271_76_79_280_81_N_GenomeWideSNP_6_A04_1346840
AMAZE_p_TCGASNP_b86_87_88_N_GenomeWideSNP_6_A11_735446
WHIPS_p_1TCGA_220_235_N_GenomeWideSNP_6_E06_1231034
ENDUE_p_TCGA_271_76_79_280_81_N_GenomeWideSNP_6_C05_1346858
WHIRR_p_TCGA_168_169_170_redo_N_GenomeWideSNP_6_C02_845262
CONGA_p_TCGA_b_317_318_319_NSP_GenomeWideSNP_6_A04_1365272
CODON_p_TCGA_293_294_295_N_GenomeWideSNP_6_G06_1343670
HORNS_p_TCGA_b110_113_SNP_N_GenomeWideSNP_6_A05_772296
AMAZE_p_TCGASNP_b86_87_88_N_GenomeWideSNP_6_C08_735516
WHIRR_p_TCGA_168_169_170_redo_N_GenomeWideSNP_6_C10_845098
ENDUE_p_TCGA_271_76_79_280_81_N_GenomeWideSNP_6_A05_1346796
XYLEM_p_TCGASNP_207_212_N_GenomeWideSNP_6_E02_1051158
WHIPS_p_1TCGA_220_235_N_GenomeWideSNP_6_G06_1230994
OKRAS_p_TCGA_198_199_200_SNP_N_GenomeWideSNP_6_D05_955386
WAXEN_p_TCGA_252_253_N_GenomeWideSNP_6_C02_1270572
ENDUE_p_TCGA_271_76_79_280_81_N_GenomeWideSNP_6_E03_1346846
OKRAS_p_TCGA_198_199_200_SNP_N_GenomeWideSNP_6_G02_955376
CODON_p_TCGA_293_294_295_N_GenomeWideSNP_6_F12_1343606
ENDUE_p_TCGA_271_76_79_280_81_N_GenomeWideSNP_6_B05_1346898
SNOWS_p_TCGA_Batch_309_310_NSP_GenomeWideSNP_6_A10_1361998
SNOWS_p_TCGA_Batch_309_310_NSP_GenomeWideSNP_6_C07_1362144
SNOWS_p_TCGA_Batch_309_310_NSP_GenomeWideSNP_6_A04_1362132
WHIPS_p_1TCGA_220_235_N_GenomeWideSNP_6_F01_1230902
BISON_p_TCGA_164_173_175_SNP_N_GenomeWideSNP_6_H04_863300
WHIPS_p_1TCGA_220_235_N_GenomeWideSNP_6_E12_1230956
AMAZE_p_TCGASNP_b86_87_88_N_GenomeWideSNP_6_F02_735478
BASIC_p_TCGASNP_219_221_223_N_GenomeWideSNP_6_F04_1148722
WRIED_p_TCGA_143_147_150_Hahn_N_GenomeWideSNP_6_D03_800004
WHIPS_p_1TCGA_220_235_N_GenomeWideSNP_6_G12_1230882
HORNS_p_TCGA_b110_113_SNP_N_GenomeWideSNP_6_E01_772322
CONGA_p_TCGA_b_317_318_319_NSP_GenomeWideSNP_6_A12_1365192
OKRAS_p_TCGA_198_199_200_SNP_N_GenomeWideSNP_6_E12_955440
BISON_p_TCGA_164_173_175_SNP_N_GenomeWideSNP_6_H12_863324
CONGA_p_TCGA_b_317_318_319_NSP_GenomeWideSNP_6_A02_1365296
CONGA_p_TCGA_b_317_318_319_NSP_GenomeWideSNP_6_A06_1365314
WHIPS_p_1TCGA_220_235_N_GenomeWideSNP_6_D10_1231016
WHIRR_p_TCGA_168_169_170_redo_N_GenomeWideSNP_6_C03_845100
HORNS_p_TCGA_b110_113_SNP_N_GenomeWideSNP_6_D11_772290
XYLEM_p_TCGASNP_207_212_N_GenomeWideSNP_6_B06_1051308
BISON_p_TCGA_164_173_175_SNP_N_GenomeWideSNP_6_H11_863386
WHIPS_p_1TCGA_220_235_N_GenomeWideSNP_6_F12_1231012
ENDUE_p_TCGA_271_76_79_280_81_N_GenomeWideSNP_6_F04_1346904
AVISO_p_4TCGA_242_238_250_mN_GenomeWideSNP_6_A02_1231630

RUNIC_p_2TCGA235_237_250_242_mN_GenomeWideSNP_6_A04_1231116
OKRAS_p_TCGA_198_199_200_SNP_N_GenomeWideSNP_6_C11_955468
HORNS_p_TCGA_b110_113_SNP_N_GenomeWideSNP_6_B08_772304
COPAL_p_TCGAb_337_338_339_NSP_GenomeWideSNP_6_B02_1373644
FRUIT_p_TCGAb_327_328_329_NSP_GenomeWideSNP_6_E02_1367874
HORNS_p_TCGA_b110_113_SNP_N_GenomeWideSNP_6_A06_772372
WAXEN_p_TCGA_252_253_N_GenomeWideSNP_6_A07_1270578
TYPED_p_TCGA_188_192_Mirn_SNP_N_GenomeWideSNP_6_C12_913854
SNOWS_p_TCGA_Batch_309_310_NSP_GenomeWideSNP_6_C11_1362150
WHIPS_p_1TCGA_220_235_N_GenomeWideSNP_6_D09_1230954
WHIPS_p_1TCGA_220_235_N_GenomeWideSNP_6_G05_1231046
LOLLS_p_TCGASNP_223_225_N_GenomeWideSNP_6_G09_1152018
FRUIT_p_TCGAb_327_328_329_NSP_GenomeWideSNP_6_D06_1367872
COPAL_p_TCGAb_337_338_339_NSP_GenomeWideSNP_6_B05_1373572
FOXED_p_TCGA_b122_128_SNP_N_GenomeWideSNP_6_D11_781766
WAXEN_p_TCGA_252_253_N_GenomeWideSNP_6_A03_1270714
AMAZE_p_TCGASNP_b86_87_88_N_GenomeWideSNP_6_E11_735426
FOXED_p_TCGA_b122_128_SNP_N_GenomeWideSNP_6_F05_781904
SNOWS_p_TCGA_Batch_309_310_NSP_GenomeWideSNP_6_A08_1362082
HORNS_p_TCGA_b110_113_SNP_N_GenomeWideSNP_6_E04_772238
CODON_p_TCGA_293_294_295_N_GenomeWideSNP_6_F06_1343648
COPAL_p_TCGAb_337_338_339_NSP_GenomeWideSNP_6_C03_1373560
CODON_p_TCGA_293_294_295_N_GenomeWideSNP_6_G01_1343702
FRUIT_p_TCGAb_327_328_329_NSP_GenomeWideSNP_6_E08_1367830
SNOWS_p_TCGA_Batch_309_310_NSP_GenomeWideSNP_6_B08_1362054
WHIPS_p_1TCGA_220_235_N_GenomeWideSNP_6_E04_1230880
WRIED_p_TCGA_143_147_150_Hahn_N_GenomeWideSNP_6_H07_799908
WHIPS_p_1TCGA_220_235_N_GenomeWideSNP_6_F11_1231022
WRIED_p_TCGA_143_147_150_Hahn_N_GenomeWideSNP_6_F06_799978
LOLLS_p_TCGASNP_223_225_N_GenomeWideSNP_6_F05_1151894
WHIPS_p_1TCGA_220_235_N_GenomeWideSNP_6_H07_1231050
ENDUE_p_TCGA_271_76_79_280_81_N_GenomeWideSNP_6_G03_1346830
LOLLS_p_TCGASNP_223_225_N_GenomeWideSNP_6_C05_1151902
OKRAS_p_TCGA_198_199_200_SNP_N_GenomeWideSNP_6_C06_955368
FOXED_p_TCGA_b122_128_SNP_N_GenomeWideSNP_6_F12_781758
FRUIT_p_TCGAb_327_328_329_NSP_GenomeWideSNP_6_D02_1367886
OKRAS_p_TCGA_198_199_200_SNP_N_GenomeWideSNP_6_F03_955462
COPAL_p_TCGAb_337_338_339_NSP_GenomeWideSNP_6_C05_1373674
WAXEN_p_TCGA_252_253_N_GenomeWideSNP_6_C05_1270692
AVISO_p_4TCGA_242_238_250_mN_GenomeWideSNP_6_A09_1231536
FRUIT_p_TCGAb_327_328_329_NSP_GenomeWideSNP_6_D12_1367864
XYLEM_p_TCGASNP_207_212_N_GenomeWideSNP_6_B04_1051198
ENDUE_p_TCGA_271_76_79_280_81_N_GenomeWideSNP_6_D05_1346754
LOLLS_p_TCGASNP_223_225_N_GenomeWideSNP_6_C06_1152032
TYPED_p_TCGA_188_192_Mirn_SNP_N_GenomeWideSNP_6_C11_913922
OKRAS_p_TCGA_198_199_200_SNP_N_GenomeWideSNP_6_C08_955374
WHIRR_p_TCGA_168_169_170_redo_N_GenomeWideSNP_6_C08_845224
COPAL_p_TCGAb_337_338_339_NSP_GenomeWideSNP_6_C10_1373566
AVISO_p_4TCGA_242_238_250_mN_GenomeWideSNP_6_A05_1231574
CODON_p_TCGA_293_294_295_N_GenomeWideSNP_6_G12_1343642
OKRAS_p_TCGA_198_199_200_SNP_N_GenomeWideSNP_6_F10_955324
SNOWS_p_TCGA_Batch_309_310_NSP_GenomeWideSNP_6_A12_1362074
XYLEM_p_TCGASNP_207_212_N_GenomeWideSNP_6_A06_1051212
COPAL_p_TCGAb_337_338_339_NSP_GenomeWideSNP_6_A04_1373568
WAXEN_p_TCGA_252_253_N_GenomeWideSNP_6_A04_1270742
AVISO_p_4TCGA_242_238_250_mN_GenomeWideSNP_6_A03_1231578
WHIPS_p_1TCGA_220_235_N_GenomeWideSNP_6_H02_1230922
AVISO_p_4TCGA_242_238_250_mN_GenomeWideSNP_6_A07_1231484
CONGA_p_TCGA_b_317_318_319_NSP_GenomeWideSNP_6_B06_1365250
WAXEN_p_TCGA_252_253_N_GenomeWideSNP_6_A11_1270596
SNOWS_p_TCGA_Batch_309_310_NSP_GenomeWideSNP_6_B02_1362062
COPAL_p_TCGAb_337_338_339_NSP_GenomeWideSNP_6_C08_1373618
WHIRR_p_TCGA_168_169_170_redo_N_GenomeWideSNP_6_C12_845256
WAXEN_p_TCGA_252_253_N_GenomeWideSNP_6_C01_1270618
HORNS_p_TCGA_b110_113_SNP_N_GenomeWideSNP_6_F07_772336
COPAL_p_TCGAb_337_338_339_NSP_GenomeWideSNP_6_A07_1373574
XYLEM_p_TCGASNP_207_212_N_GenomeWideSNP_6_E01_1051160
FRUIT_p_TCGAb_327_328_329_NSP_GenomeWideSNP_6_D08_1367900
FOXED_p_TCGA_b122_128_SNP_N_GenomeWideSNP_6_D10_781864
CONGA_p_TCGA_b_317_318_319_NSP_GenomeWideSNP_6_A08_1365346

Supplementary table 6

Sample annotations for all tumors included in Aine et al.

Gene mutations (m) indicated as 0 (WT) and 1 (Mutant)
Gene copy-number status (cna) indicated as -2 (deletion), -1 (loss), 0 (Neutral), 1 (gain) and 2 (amplification)
Centroid correlations were calculated using Pearson correlation
Cells with NA indicate that the sample was not included in the TCGA publication on BLCA (Nature, Volume 507 Number 7492, 315-322, Mar. 20, 2014 [doi:10.1038])

SampleID	Histology	TCGAclass	Two_group	Three_group	Six_group	UNC	MDA	Lund	FGFR3m	FGFR3cna	PIK3CAm	RASm	chr9pDel	chr9qDel	CDKN2Acna	TP53m	MDM2cna	RB1m	RB1cna	E2F3cna	cor.UNC.Basal	cor.UNC.Luminal	cor.MDA.Basal	cor.MDA.tp53like	cor.MDA.Luminal	cor.Lund.MS1a	cor.Lund.MS1b	cor.Lund.MS2a1	cor.Lund.MS2a2	cor.Lund.MS2b1	cor.Lund.MS2b2.1	cor.Lund.MS2b2.2
TCGA-DK-A6AV	NA	NA	2	2	21	Luminal	Luminal	MS1b	0	0	0	0	0	0	0	0	0	0	0	0	-0.537	0.796	-0.335	-0.189	0.487	0.065	0.475	0.167	0.024	-0.334	-0.214	-0.388
TCGA-DK-A3WX	Non-papillary	3	1	1	11	Basal	Basal	MS2b2.2	0	0	0	0	0	0	-1	0	0	0	0	0	0.843	-0.796	0.744	-0.141	-0.517	-0.293	-0.532	-0.411	-0.064	0.389	0.481	0.754
TCGA-FD-A3SQ	Non-papillary	2	2	3	31	Basal	TP53-like	MS2b1	0	0	0	0	0	0	-1	0	0	0	0	0	0.066	-0.286	-0.062	0.583	-0.269	0.139	-0.046	-0.358	-0.196	0.444	-0.054	-0.09
TCGA-DK-A1AC	Non-papillary	2	2	3	31	Luminal	Luminal	MS2a2	0	0	1	1	0	0	1	1	0	0	-1	2	-0.075	0.296	-0.136	-0.067	0.119	-0.414	-0.292	0.421	0.532	0.214	-0.342	-0.039
TCGA-FD-A43N	NA	NA	2	3	31	Luminal	Luminal	MS1b	0	0	1	0	0	0	0	1	0	0	0	2	-0.62	0.757	-0.183	0.157	0.286	0.014	0.222	0.221	0.16	-0.051	-0.296	-0.424
TCGA-FD-A5BU	NA	NA	1	1	11	Basal	Basal	MS2b2.2	0	0	0	0	0	0	-1	0	0	0	0	0	0.889	-0.822	0.696	0.095	-0.568	-0.173	-0.493	-0.536	-0.203	0.449	0.532	0.709
TCGA-FD-A6TG	NA	NA	2	3	31	Luminal	TP53-like	MS2b1	0	0	0	0	0	0	0	0	1	0	0	1	0.096	0.231	-0.174	0.454	0.019	-0.073	-0.018	-0.021	0.134	0.293	-0.227	-0.206
TCGA-BT-A20J	Non-papillary	4	1	1	12	Basal	Basal	MS2b1	0	0	0	0	0	0	-2	1	0	0	0	0	0.701	-0.639	0.241	0.031	-0.319	-0.312	-0.488	-0.137	0.177	0.43	0.118	0.414
TCGA-E7-A677	NA	NA	2	2	21	Luminal	TP53-like	MS1b	0	0	0	1	1	0	-2	0	0	0	0	0	-0.092	0.007	-0.086	0.101	0.08	0.267	0.39	-0.129	-0.275	-0.219	-0.059	-0.202
TCGA-CF-A47Y	NA	NA	2	2	22	Luminal	Luminal	MS1a	1	0	0	0	0	0	0	1	0	0	0	0	-0.631	0.562	-0.48	0.15	0.296	0.758	0.681	-0.147	-0.589	-0.395	-0.202	-0.578
TCGA-FD-A43P	NA	NA	2	3	31	Luminal	Luminal	MS2a2	0	0	0	1	0	0	0	1	0	1	0	2	-0.33	0.409	-0.182	-0.007	0.189	-0.368	-0.257	0.361	0.544	0.245	-0.367	-0.118
TCGA-LT-A5Z6	NA	NA	2	2	21	Luminal	Luminal	MS2a1	0	0	0	0	0	0	0	1	0	0	-2	1	-0.725	0.787	-0.382	-0.152	0.588	-0.062	0.258	0.647	0.353	-0.387	-0.405	-0.485
TCGA-GC-A3YS	Non-papillary	3	1	1	11	Basal	Basal	MS2b2.2	0	0	0	0	0	0	0	1	0	1	-1	0	0.838	-0.848	0.651	-0.038	-0.608	-0.308	-0.573	-0.4	-0.035	0.439	0.459	0.747
TCGA-S5-A6DX	NA	NA	2	3	31	Luminal	TP53-like	MS2a2	0	0	0	0	0	0	-2	0	1	0	0	0	-0.41	0.314	-0.054	0.306	0.082	-0.202	-0.074	0.016	0.306	0.299	-0.208	-0.18
TCGA-BL-A0C8	Papillary	1	2	2	21	Luminal	Luminal	MS2a1	0	1	0	0	0	0	-1	0	0	0	0	0	-0.579	0.729	-0.438	0.14	0.346	-0.106	0.169	0.471	0.349	-0.12	-0.485	-0.433
TCGA-GC-A6I1	NA	NA	1	1	11	Basal	Basal	MS2b2.2	0	0	0	0	0	0	0	1	0	0	0	0	0.841	-0.854	0.727	-0.212	-0.515	-0.247	-0.56	-0.455	-0.069	0.448	0.449	0.73
TCGA-CU-A5W6	NA	NA	2	3	31	Luminal	Luminal	MS1b	0	1	1	0	0	0	-2	0	0	0	0	0	-0.713	0.644	-0.146	-0.033	0.39	0.034	0.261	0.176	0.072	-0.251	0.019	-0.302
TCGA-DK-A3WY	Non-papillary	4	1	1	12	Basal	TP53-like	MS2b1	0	0	0	0	0	0	0	0	0	0	0	0	0.824	-0.826	0.29	0.296	-0.528	-0.413	-0.61	-0.179	0.244	0.667	0.019	0.444
TCGA-FD-A5BS	NA	NA	1	1	12	Basal	Basal	MS2b1	0	0	0	0	0	0	0	0	0	0	0	0	0.787	-0.819	0.365	0.324	-0.571	-0.355	-0.626	-0.293	0.164	0.75	0.09	0.445
TCGA-FD-A3B6	Papillary	4	1	1	12	Basal	Basal	MS2b2.2	0	0	1	0	0	0	0	1	0	1	0	2	0.752	-0.758	0.582	-0.227	-0.449	-0.413	-0.608	-0.197	0.143	0.411	0.316	0.687
TCGA-FD-A3B5	Non-papillary	3	1	1	11	Basal	Basal	MS2b2.2	0	0	1	0	0	0	-2	0	0	0	0	0	0.721	-0.642	0.625	-0.164	-0.299	-0.034	-0.216	-0.324	-0.244	-0.003	0.509	0.556
TCGA-FT-A3EE	Papillary	1	1	1	11	Basal	Basal	MS1b	0	0	0	1	0	0	0	0	0	0	1	0	0.181	-0.343	0.032	-0.13	0.015	0.211	0.247	-0.236	-0.331	-0.14	0.169	-0.006
TCGA-FD-A3B3	Papillary	3	1	1	11	Basal	Basal	MS2b2.2	0	0	0	0	0	0	0	0	0	0	0	1	0.918	-0.837	0.76	0.046	-0.569	-0.376	-0.662	-0.417	0.022	0.555	0.433	0.788
TCGA-FD-A43U	NA	NA	2	3	31	Luminal	TP53-like	MS2b1	0	0	0	0	0	0	-1	0	0	0	0	0	-0.015	0.049	-0.018	0.345	-0.131	-0.116	-0.146	-0.106	0.131	0.448	-0.166	-0.114
TCGA-BT-A3PK	Papillary	3	1	1	11	Basal	Basal	MS2b2.2	0	0	0	0	0	0	0	0	0	0	0	0	0.814	-0.744	0.659	-0.049	-0.504	-0.333	-0.575	-0.369	0.012	0.469	0.391	0.711
TCGA-FD-A5C1	NA	NA	1	1	11	Basal	Basal	MS2b2.2	0	0	1	0	0	0	0	0	0	0	0	0	0.79	-0.798	0.669	-0.025	-0.526	-0.337	-0.607	-0.427	0.006	0.516	0.47	0.727
TCGA-E7-A4IJ	NA	NA	2	2	21	Luminal	Luminal	MS2a1	0	0	1	0	0	0	0	0	0	0	-2	1	-0.698	0.762	-0.383	-0.117	0.425	0.08	0.257	0.45	0.191	-0.31	-0.354	-0.448
TCGA-GV-A40E	Non-papillary	3	1	1	11	Basal	Basal	MS2b2.2	0	0	0	0	0	0	0	0	0	0	0	2	0.731	-0.708	0.571	-0.226	-0.38	-0.356	-0.459	-0.208	0.098	0.221	0.313	0.678
TCGA-GD-A76B	NA	NA	2	2	21	Luminal	Luminal	MS2a1	0	0	1	0	0	0	-1	0	0	0	0	0	-0.74	0.83	-0.387	0.154	0.472	0.034	0.174	0.344	0.237	-0.044	-0.406	-0.504
TCGA-BT-A20T	Non-papillary	2	2	3	31	Basal	TP53-like	MS2b2.1	1	1	0	0	1	1	-2	0	0	0	0	0	0.054	0.046	0.063	0.183	0.018	0.214	0.138	-0.375	-0.306	0.113	0.243	-0.07
TCGA-UY-A78O	NA	NA	2	2	21	Luminal	Luminal	MS1b	0	0	0	0	0	0	0	1	0	0	0	0	-0.764	0.84	-0.513	0.11	0.554	0.218	0.561	0.284	0.01	-0.341	-0.403	-0.653
TCGA-BT-A0S7	Non-papillary	3	2	3	32	Basal	Basal	MS2b2.2	1	0	0	0	0	0	-2	0	2	0	1	0	0.653	-0.638	0.348	0.016	-0.303	-0.227	-0.388	-0.129	0.061	0.241	0.22	0.441
TCGA-H4-A2HQ	Non-papillary	1	2	2	22	Luminal	Luminal	MS1b	1	0	0	0	0	0	-2	0	2	0	0	0	-0.203	0.316	-0.162	-0.276	0.289	0.328	0.331	0.016	-0.268	-0.462	0.116	-0.125
TCGA-KQ-A41N	NA	NA	2	2	21	Luminal	Luminal	MS1b	0	0	0	0	0	0	0	0	0	0	0	1	-0.459	0.591	-0.392	0.083	0.378	0.204	0.466	0.297	-0.011	-0.372	-0.341	-0.504
TCGA-BT-A0YX	Non-papillary	3	1	1	11	Basal	Basal	MS2b2.1	0	0	0	0	0	0	-2	0	0	0	0	0	0.713	-0.661	0.53	-0.217	-0.297	-0.042	-0.292	-0.456	-0.263	0.149	0.627	0.578
TCGA-CU-A0YO	Non-papillary	2	2	3	31	Luminal	TP53-like	MS1a	0	0	0	0	0	0	0	0	0	0	0	0	-0.449	0.275	-0.142	0.464	0.066	0.397	0.222	-0.333	-0.396	0.148	0.064	-0.291
TCGA-FD-A6TC	NA	NA	2	3	31	Basal	TP53-like	MS2a2	0	0	0	0	0	0	0	1	0	1	0	0	0.426	-0.343	-0.07	0.189	-0.076	-0.206	-0.074	0.06	0.169	0.135	-0.112	0.029
TCGA-BT-A2LA	NA	NA	2	3	32	Basal	TP53-like	MS2a2	0	0	0	0	0	0	0	1	0	1	0	2	0.556	-0.384	0.095	0.111	-0.203	-0.228	-0.24	0.268	0.292	0.081	-0.258	0.112
TCGA-PQ-A6FN	NA	NA	1	1	11	Basal	Basal	MS2b2.2	0	0	0	0	0	0	0	1	0	1	0	0	0.682	-0.694	0.631	-0.025	-0.329	-0.222	-0.372	-0.313	-0.062	0.236	0.411	0.586
TCGA-GC-A3RC	Non-papillary	3	1	1	11	Basal	Basal	MS2b2.2	0	0	0	0	0	0	0	1	0	0	0	0	0.831	-0.801	0.717	-0.261	-0.426	-0.239	-0.456	-0.356	-0.078	0.257	0.426	0.726
TCGA-DK-A2I2	Non-papillary	3	1	1	11	Basal	Basal	MS2b2.2	0	0	0	0	0	0	0	1	0	0	0	1	0.88	-0.835	0.715	-0.083	-0.515	-0.308	-0.579	-0.422	-0.051	0.478	0.456	0.747
TCGA-FD-A3B7	Non-papillary	4	1	1	12	Basal	Basal	MS2b2.2	0	0	0	0	0	0	0	1	0	1	0	0	0.882	-0.861	0.67	0.068	-0.636	-0.321	-0.618	-0.456	-0.045	0.591	0.423	0.728
TCGA-FD-A62S	NA	NA	1	1	12	Basal	Basal	MS2b2.2	0	0	0	0	0	0	-1	1	0	0	0	0	0.912	-0.879	0.673	0.189	-0.679	-0.308	-0.627	-0.462	-0.048	0.618	0.373	0.723
TCGA-G2-A2EL	NA	NA	2	3	32	Basal	Basal	MS2a1	0	0	0	0	0	1	0	1	0	1	0	0	0.49	-0.291	0.166	-0.088	-0.056	-0.197	-0.16	0.262	0.21	-0.116	-0.102	0.192
TCGA-BT-A20U	Non-papillary	3	1	1	11	Basal	Basal	MS2b2.1	0	0	0	0	0	0	-2	1	0	0	0	0	0.703	-0.654	0.477	-0.094	-0.294	-0.032	-0.248	-0.339	-0.23	0.077	0.526	0.51
TCGA-BT-A42F	NA	NA	1	1	11	Basal	Basal	MS2b2.2	0	0	0	0	0	0	0	1	0	0	0	0	0.729	-0.725	0.635	-0.245	-0.371	-0.27	-0.485	-0.385	-0.022	0.352	0.502	0.649
TCGA-FT-A61P	NA	NA	1	1	12	Basal	Basal	MS2b1	0	0	0	0	0	0	0	0	2	0	0	0	0.649	-0.686	0.339	0.236	-0.486	-0.303	-0.507	-0.221	0.074	0.506	0.193	0.469
TCGA-DK-A1A6	Non-papillary	1	2	3	31	Luminal	Luminal	MS2a2	0	0	1	0	0	1	0	1	0	0	0	2	-0.133	0.359	-0.17	-0.306	0.341	-0.273	-0.016	0.283	0.391	-0.016	-0.229	-0.122
TCGA-GU-A767	NA	NA	2	3	31	Luminal	TP53-like	MS1b	0	0	0	0	0	0	0	1	0	0	0	1	-0.654	0.761	-0.354	0.449	0.277	0.12	0.35	0.268	0.1	-0.126	-0.447	-0.549
TCGA-GV-A3JX	Non-papillary	2	2	2	21	Luminal	Luminal	MS2a1	0	0	1	0	0	0	0	1	0	1	0	0	-0.303	0.45	0.07	-0.132	0.181	-0.257	-0.175	0.412	0.364	-0.079	-0.086	-0.005
TCGA-CF-A3MH	Papillary	1	2	2	22	Luminal	Luminal	MS1b	0	1	0	0	1	1	-2	0	1	0	0	0	-0.599	0.515	-0.318	-0.166	0.417	0.492	0.66	-0.035	-0.408	-0.594	0.076	-0.406
TCGA-DK-A1AF	Non-papillary	2	2	3	31	Basal	TP53-like	MS2b1	0	0	0	0	0	0	0	0	0	0	0	0	0.096	-0.13	0.276	0.433	-0.288	-0.063	-0.221	-0.391	-0.128	0.555	0.19	0.107
TCGA-GC-A3WC	Non-papillary	3	1	1	11	Basal	Basal	MS2b2.2	0	0	1	0	0	0	0	1	0	0	0	0	0.8	-0.771	0.484	0.142	-0.458	-0.138	-0.474	-0.42	-0.125	0.408	0.407	0.587
TCGA-BL-A13J	Papillary	2	2	3	31	Luminal	TP53-like	MS1b	0	0	0	0	0	0	-2	1	0	0	-1	1	-0.112	0.13	0.083	0.135	0.046	-0.032	0.088	0.015	-0.031	-0.052	0.032	-0.022
TCGA-BT-A20W	Non-papillary	2	2	3	31	Luminal	Luminal	MS2a1	0	0	0	1	0	0	-1	0	0	0	0	1	-0.682	0.818	-0.445	0.167	0.469	-0.04	0.288	0.403	0.282	-0.095	-0.562	-0.543
TCGA-CU-A0YN	Non-papillary	3	1	1	11	Basal	Basal	MS2b2.2	0	0	0	0	0	0	0	0	0	1	0	1	0.815	-0.799	0.695	-0.186	-0.348	-0.299	-0.455	-0.332	-0.04	0.291	0.463	0.692
TCGA-G2-A2EF	Non-papillary	4	1	1	11	Basal	Basal	MS2b2.2	0	0	0	1	0	0	-2	0	0	0	0	1	0.88	-0.834	0.575	-0.207	-0.451	-0.267	-0.477	-0.304	-0.014	0.355	0.281	0.647
TCGA-FD-A3SN	Papillary	2	1	3	31	Basal	Basal	MS2b2.2	0	0	1	0	0	0	0	0	0	0	0	0	0.524	-0.505	0.306	-0.002	-0.245	-0.189	-0.191	-0.164	0.024	0.109	0.156	0.368
TCGA-CF-A3MF	Papillary	1	2	2	22	Luminal	Luminal	MS1b	0	0	0	0	1	1	-1	0	1	0	0	0	-0.864	0.716	-0.436	0.137	0.418	0.375	0.62	0.147	-0.141	-0.411	-0.37	-0.608
TCGA-DK-A3IK	Papillary	2	2	3	31	Luminal	Luminal	MS1b	0	0	0	0	0	0	-1	1	0	0	0	0	-0.713	0.751	-0.459	0.39	0.423	0.205	0.462	0.184	0.001	-0.131	-0.489	-0.615
TCGA-GD-A6C6	NA	NA	2	2	21	Luminal	Luminal	MS2a1	0	0	1	0	0	0	-2	1	0	0	0	0	-0.17	0.02	-0.193	-0.049	0.138	0.057	0.169	0.209	0.106	-0.254	-0.134	-0.205
TCGA-GU-A763	NA	NA	2	2	22	Luminal	Luminal	MS1b	1	0	0	0	0	1	-2	0	0	0	0	0	-0.778	0.686	-0.378	-0.098	0.442	0.618	0.664	-0.036	-0.457	-0.551	-0.059	-0.496
TCGA-FD-A62P	NA	NA	1	3	31	Basal	Basal	MS2b2.2	0	0	1	0	0	0	0	0	1	0	0	0	0.485	-0.449	0.307	0.165	-0.208	-0.272	-0.338	-0.272	0.092	0.371	0.191	0.387
TCGA-E7-A5KE	NA	NA	2	2	21	Luminal	Luminal	MS2a1	0	1	0	0	1	0	-1	0	0	0	-1	1	-0.847	0.919	-0.471	-0.197	0.589	0.061	0.489	0.51	0.164	-0.616	-0.309	-0.452
TCGA-KQ-A41P	NA	NA	2	2	21	Luminal	TP53-like	MS1b	0	0	0	0	0	0	0	1	0	0	0	0	-0.539	0.493	-0.172	0.27	0.192	0.108	0.155	-0.006	-0.033	0.04	-0.066	-0.288
TCGA-DK-A2I4	Papillary	4	1	1	12	Basal	Basal	MS2b1	0	0	0	0	0	0	0	0	0	0	-1	1	0.648	-0.677	0.369	0.144	-0.476	-0.36	-0.598	-0.216	0.178	0.628	0.138	0.452
TCGA-MV-A51V	NA	NA	2	2	21	Luminal	Luminal	MS2a1	0	0	0	0	0	0	0	0	0	1	-1	0	-0.783	0.832	-0.46	0.165	0.449	0.049	0.351	0.568	0.231	-0.348	-0.458	-0.579
TCGA-E7-A678	NA	NA	2	2	22	Luminal	Luminal	MS1a	1	0	0	0	0	0	0	0	0	0	0	0	-0.717	0.699	-0.482	-0.058	0.423	0.814	0.637	-0.155	-0.59	-0.432	-0.122	-0.565
TCGA-GU-A42R	Papillary	1	2	2	22	Luminal	Luminal	MS1a	1	0	0	0	0	0	-2	0	1	0	0	1	-0.394	0.437	-0.177	-0.146	0.32	0.465	0.453	-0.096	-0.414	-0.47	0.206	-0.244
TCGA-FD-A62O	NA	NA	2	2	21	Luminal	Luminal	MS1b	0	0	0	0	1	1	-1	0	0	0	0	0	-0.681	0.467	-0.426	0.096	0.446	0.261	0.576	0.181	-0.099	-0.328	-0.37	-0.578
TCGA-CU-A3KJ	Non-papillary	3	1	1	11	Basal	Basal	MS2b2.2	0	0	0	0	1	1	-2	1	0	0	0	0	0.81	-0.711	0.612	-0.283	-0.363	-0.206	-0.409	-0.275	-0.084	0.161	0.452	0.666
TCGA-DK-A2HX	Non-papillary	2	2	3	31	Basal	TP53-like	MS2b1	0	0	0	0	0	0	0	0	0	0	0	0	0.255	-0.401	-0.073	0.496	-0.221	-0.206	-0.277	0.061	0.207	0.44	-0.223	-0.015
TCGA-FD-A6TE	NA	NA	2	2	21	Luminal	Luminal	MS2a1	0	0	0	0	0	0	0	1	0	1	0	0	-0.362	0.374	-0.146	-0.294	0.304	-0.019	0.099	0.433	0.14	-0.429	0	-0.106
TCGA-LC-A66R	NA	NA	1	1	11	Basal	Basal	MS2b2.2	0	0	0	0	0	0	0	1	0	1	0	2	0.742	-0.735	0.638	-0.146	-0.439	-0.374	-0.593	-0.263	0.085	0.385	0.375	0.727
TCGA-GC-A3RB	Non-papillary	1	2	3	32	Luminal	Luminal	MS2a1	0	-2	0	0	0	0	0	1	0	0	-1	2	-0.101	0.291	-0.077	-0.326	0.25	-0.001	0.13	0.267	0.122	-0.365	-0.112	-0.05
TCGA-FD-A3N6	NA	NA	1	1	11	Basal	Basal	MS2b2.2	0	0	0	0	0	0	-2	0	0	0	0	0	0.758	-0.691	0.682	-0.259	-0.341	-0.072	-0.304	-0.371	-0.225	0.091	0.477	0.643
TCGA-K4-A5RJ	NA	NA	1	1	12	Basal	Basal	MS2b1	0	0	0	0	0	0	0	1	0	1	0	0	0.667	-0.673	0.386	-0.012	-0.43	-0.395	-0.613	-0.125	0.244	0.527	0.128	0.492
TCGA-GV-A3JW	Non-papillary	1	2	2	21	Luminal	Luminal	MS1b	0	0	0	0	0	0	-2	1	0	0	-2	0	-0.705	0.883	-0.444	-0.017	0.603	0.265	0.561	0.475	0.03	-0.542	-0.448	-0.641
TCGA-DK-A3IN	Papillary	2	2	3	31	Basal	Basal	MS2b1	0	0	0	0	0	0	0	1	0	1	0	0	0.473	-0.358	0.428	0.079	-0.287	-0.359	-0.417	-0.1	0.216	0.425	0.035	0.351
TCGA-CF-A5U8	NA	NA	2	2	22	Luminal	Luminal	MS1b	1	0	0	0	1	1	-2	1	0	0	0	0	-0.843	0.797	-0.532	0.005	0.507	0.669	0.762	0.008	-0.463	-0.502	-0.285	-0.666
TCGA-BT-A3PJ	Papillary	3	1	1	11	Basal	Basal	MS2b2.2	0	0	0	0	0	0	1	1	0	1	-1	2	0.851	-0.826	0.648	-0.268	-0.431	-0.294	-0.498	-0.301	-0.027	0.273	0.421	0.74
TCGA-DK-A1AA	Papillary	1	2	2	22	Luminal	Luminal	MS1a	0	0	0	0	1	1	-2	0	0	0	0	0	-0.655	0.481	-0.244	-0.073	0.322	0.643	0.626	-0.314	-0.663	-0.385	0.199	-0.363
TCGA-GV-A6ZA	NA	NA	2	2	21	Luminal	Luminal	MS1b	0	0	0	0	0	0	-2	0	0	0	0	0	-0.755	0.766	-0.405	-0.27	0.512	0.278	0.478	0.247	-0.052	-0.474	-0.167	-0.487
TCGA-GV-A3JV	Non-papillary	2	2	2	21	Luminal	Luminal	MS1b	0	0	0	0	0	1	0	0	0	0	0	0	-0.687	0.651	-0.384	0.167	0.278	0.014	0.263	0.188	0.139	0.012	-0.447	-0.442
TCGA-DK-A1A5	Non-papillary	2	2	3	31	Basal	Basal	MS2b2.2	0	0	1	0	0	0	-2	1	0	0	0	0	0.501	-0.493	0.131	0.101	-0.233	-0.192	-0.131	-0.205	-0.029	0.212	0.148	0.269
TCGA-CF-A47S	NA	NA	2	2	22	Luminal	Luminal	MS1b	0	0	0	0	0	0	0	0	0	0	0	0	-0.881	0.742	-0.44	-0.021	0.483	0.561	0.687	-0.071	-0.403	-0.386	-0.202	-0.597
TCGA-GD-A3OQ	Papillary	1	1	1	11	Basal	Basal	MS2b2.1	0	0	0	1	0	0	-2	0	0	0	0	0	0.33	-0.329	0.287	-0.284	0.02	0.199	0.276	-0.204	-0.364	-0.362	0.391	0.116
TCGA-FD-A6TA	NA	NA	2	3	31	Luminal	TP53-like	MS2b1	0	0	0	0	0	0	-2	0	0	0	0	1	-0.437	0.478	-0.21	0.291	0.161	-0.011	0.048	-0.071	0.058	0.243	-0.104	-0.248
TCGA-CF-A3MI	Papillary	1	2	2	22	Luminal	Luminal	MS1b	0	-1	0	1	0	0	0	0	1	0	0	0	-0.845	0.818	-0.382	0.026	0.454	0.335	0.533	0.062	-0.16	-0.303	-0.21	-0.546
TCGA-DK-A1AD	Non-papillary	2	2	3	31	Luminal	Luminal	MS2a1	0	0	0	0	0	0	0	1	0	0	0	2	-0.692	0.711	-0.406	0.22	0.387	0.073	0.247	0.264	0.114	-0.118	-0.302	-0.442
TCGA-GC-A3I6	Non-papillary	3	1	1	11	Basal	Basal	MS2b2.1	0	0	1	0	0	0	0	0	0	0	0	0	0.692	-0.732	0.566	-0.172	-0.325	0	-0.271	-0.414	-0.288	0.082	0.602	0.574

TCGA-GC-A3RD	Non-papillary		1	2	3	32	Luminal	Luminal	MS1b	0	0	0	0	0	0	0	0	2	0	0	0	-0.499	0.642	-0.109	-0.192	0.443	0.184	0.387	0.175	-0.063	-0.397	-0.119	-0.31
TCGA-HQ-A5NE	NA	NA		1	1	11	Basal	Basal	MS2b2.1	0	0	1	0	0	0	0	1	0	0	0	1	0.49	-0.68	0.502	-0.049	-0.231	0.058	-0.016	-0.317	-0.301	-0.013	0.397	0.306
TCGA-CU-A3YL	Papillary		1	2	2	21	Luminal	Luminal	MS1b	0	0	0	0	1	0	-2	0	0	0	0	0	-0.845	0.817	-0.482	0.218	0.431	0.296	0.486	0.203	-0.043	-0.265	-0.359	-0.629
TCGA-CU-A72E	NA	NA		1	1	11	Basal	Basal	MS2b2.1	1	0	1	0	1	1	-2	0	0	0	0	0	0.501	-0.463	0.335	0.101	-0.185	0.18	-0.056	-0.474	-0.431	0.063	0.612	0.297
TCGA-CF-A5UA	NA	NA		2	2	22	Luminal	Luminal	MS1b	0	1	1	1	0	0	-2	0	2	0	0	1	-0.599	0.538	-0.359	-0.073	0.26	0.434	0.469	-0.003	-0.301	-0.424	-0.022	-0.354
TCGA-FD-A3SS	Non-papillary		2	1	3	31	Basal	Basal	MS2a1	0	0	0	0	0	0	0	1	-1	0	-2	0	0.308	-0.13	0.197	0.021	-0.048	-0.314	-0.229	0.313	0.313	-0.068	-0.101	0.229
TCGA-DK-A2I1	Papillary		2	2	3	31	Luminal	TP53-like	MS2a2	0	0	0	0	0	0	-2	1	0	0	0	0	-0.424	0.6	-0.282	0.36	0.23	-0.122	0.079	0.192	0.258	0.223	-0.432	-0.389
TCGA-K4-A3WU	Non-papillary		2	2	3	31	Luminal	TP53-like	MS2b1	0	0	0	0	0	0	0	0	1	0	0	0	-0.396	0.56	-0.126	0.611	0.087	-0.061	-0.044	0.041	0.147	0.352	-0.269	-0.286
TCGA-BT-A2LD	Non-papillary		4	1	3	31	Basal	Basal	MS2b2.2	0	0	0	0	0	0	0	1	0	0	0	2	0.436	-0.463	0.225	0.11	-0.226	-0.409	-0.406	0.094	0.338	0.254	-0.062	0.353
TCGA-DK-A3IV	Non-papillary		1	2	2	21	Luminal	Luminal	MS2a1	0	0	0	0	0	0	0	1	0	1	-1	0	-0.735	0.783	-0.247	-0.152	0.365	-0.077	0	0.428	0.301	-0.119	-0.23	-0.276
TCGA-CF-A1HS	Non-papillary		3	1	1	11	Basal	Basal	MS2b2.2	0	0	0	0	0	0	0	1	0	0	-1	1	0.702	-0.76	0.674	-0.244	-0.274	-0.24	-0.338	-0.191	-0.026	0.095	0.379	0.597
TCGA-FD-A5BV	NA	NA		2	3	31	Luminal	Luminal	MS1b	0	0	0	0	1	0	-1	1	0	0	0	0	-0.748	0.792	-0.393	0.045	0.353	0.149	0.379	0.356	0.022	-0.353	-0.346	-0.427
TCGA-GV-A40G	Non-papillary		1	2	2	21	Luminal	Luminal	MS2a1	0	-1	0	0	0	1	0	1	0	1	0	1	-0.762	0.648	-0.412	-0.119	0.475	-0.106	0.236	0.67	0.37	-0.422	-0.382	-0.416
TCGA-PQ-A6FI	NA	NA		1	1	11	Basal	Basal	MS2b2.2	0	0	0	0	0	0	0	1	0	1	0	2	0.833	-0.787	0.706	-0.268	-0.422	-0.222	-0.411	-0.283	-0.078	0.114	0.448	0.736
TCGA-GU-A42Q	NA	NA		1	1	11	Basal	Basal	MS2b2.2	0	0	0	0	0	0	0	1	0	0	0	0	0.802	-0.749	0.562	-0.16	-0.382	-0.026	-0.203	-0.278	-0.233	-0.053	0.428	0.568
TCGA-CF-A3MG	Papillary		1	2	2	22	Luminal	Luminal	MS1b	0	0	0	0	1	1	-2	0	0	0	0	0	-0.876	0.867	-0.453	-0.267	0.611	0.369	0.744	0.261	-0.206	-0.743	-0.166	-0.53
TCGA-FD-A5C0	NA	NA		2	3	31	Luminal	TP53-like	MS2a2	0	0	0	0	0	0	-1	0	0	1	0	0	-0.029	0.125	-0.111	0.199	0.12	-0.176	-0.026	0.174	0.19	-0.001	-0.064	-0.044
TCGA-E7-A3Y1	Papillary		1	2	2	22	Luminal	Luminal	MS1a	1	0	0	0	0	0	0	0	0	0	0	0	-0.587	0.583	-0.429	0.191	0.269	0.718	0.501	-0.184	-0.547	-0.304	-0.012	-0.479
TCGA-E7-A6MF	NA	NA		2	2	22	Luminal	Luminal	MS1a	0	0	0	1	0	0	0	0	0	0	0	0	-0.165	-0.021	-0.252	0.105	0.109	0.647	0.36	-0.221	-0.534	-0.17	-0.079	-0.324
TCGA-G2-A3VY	Non-papillary		1	2	2	21	Luminal	Luminal	MS2a1	0	0	1	0	1	0	-1	1	0	0	0	2	-0.775	0.873	-0.524	-0.142	0.618	0.125	0.458	0.618	0.219	-0.555	-0.528	-0.585
TCGA-K4-A6MB	NA	NA		2	3	31	Luminal	Luminal	MS2a1	0	1	0	0	1	0	-1	1	0	0	0	2	-0.612	0.75	-0.114	0.17	0.317	-0.045	0.233	0.327	0.227	-0.19	-0.324	-0.36
TCGA-FD-A6TB	NA	NA		1	1	12	Basal	TP53-like	MS2b1	0	0	0	0	0	0	0	0	1	0	0	0	0.529	-0.747	0.171	0.456	-0.411	-0.282	-0.502	-0.215	0.164	0.617	0.068	0.3
TCGA-GD-A3OS	Non-papillary		4	2	3	32	Basal	Basal	MS2b2.2	0	0	0	0	1	0	-2	1	0	0	0	0	0.848	-0.763	0.447	0.191	-0.508	-0.275	-0.499	-0.128	0.11	0.388	0.062	0.499
TCGA-BT-A20V	NA	NA		2	3	32	Basal	Basal	MS2b2.2	0	-1	0	0	1	0	-1	0	0	0	0	0	0.409	-0.458	0.175	-0.229	-0.071	-0.117	-0.17	0.049	0.078	-0.083	0.055	0.295
TCGA-K4-A3WV	Non-papillary		3	2	3	32	Basal	Basal	MS2b2.2	0	-1	0	0	0	0	-2	0	1	0	0	1	0.628	-0.5	0.213	0.006	-0.149	-0.076	-0.145	-0.009	0.008	-0.067	0.103	0.289
TCGA-FD-A3B4	NA	NA		1	1	11	Basal	Basal	MS2b2.2	0	0	0	0	0	0	0	0	0	0	0	0	0.887	-0.853	0.74	0.083	-0.585	-0.227	-0.525	-0.462	-0.141	0.446	0.421	0.742
TCGA-GU-A42P	NA	NA		2	2	21	Luminal	Luminal	MS2a1	0	0	0	0	0	0	0	1	0	0	0	1	-0.751	0.844	-0.457	0.002	0.447	-0.114	0.233	0.693	0.412	-0.381	-0.532	-0.441
TCGA-FJ-A3Z9	NA	NA		2	2	22	Luminal	Luminal	MS1b	0	0	0	1	0	0	0	0	0	0	0	0	-0.742	0.761	-0.328	-0.216	0.566	0.406	0.736	0.184	-0.221	-0.665	-0.273	-0.528
TCGA-FD-A6TK	NA	NA		1	1	12	Basal	Basal	MS2b2.2	0	0	0	0	0	0	0	1	0	0	0	0	0.769	-0.759	0.54	0.155	-0.575	-0.334	-0.59	-0.333	0.056	0.579	0.226	0.616
TCGA-FJ-A3Z7	Non-papillary		2	2	3	31	Luminal	TP53-like	MS2a2	0	0	0	0	0	0	0	1	0	0	0	1	-0.097	0.283	-0.202	0.28	0.02	-0.148	-0.133	0.284	0.322	0.256	-0.42	-0.255
TCGA-FD-A5BR	NA	NA		2	3	31	Luminal	TP53-like	MS2b1	0	0	0	0	0	0	-2	1	0	0	0	0	-0.352	0.447	-0.02	0.441	0.076	0.114	0.075	-0.112	-0.073	0.213	-0.11	-0.242
TCGA-C4-A0EZ	NA	NA		2	3	32	Basal	Basal	MS2a2	0	0	0	0	0	0	0	1	0	0	-2	2	0.664	-0.528	0.194	0.126	-0.17	-0.288	-0.268	0.233	0.277	0.04	-0.118	0.237
TCGA-GC-A3BM	Non-papillary		1	2	2	22	Luminal	Luminal	MS1a	1	0	0	0	0	1	-1	0	0	0	-1	0	-0.162	0.287	-0.112	-0.153	0.235	0.52	0.353	-0.231	-0.536	-0.364	0.316	-0.125
TCGA-K4-A4AC	NA	NA		1	1	11	Basal	Basal	MS2b2.2	0	0	0	0	0	0	-1	1	0	0	-1	1	0.812	-0.863	0.686	-0.178	-0.446	-0.239	-0.443	-0.372	-0.092	0.233	0.487	0.737
TCGA-G2-A2EO	Non-papillary		2	2	3	31	Basal	TP53-like	MS2b1	1	0	1	0	0	0	0	1	0	0	0	0	0.079	-0.002	-0.012	0.131	-0.092	-0.051	-0.084	-0.212	-0.04	0.295	0.093	0.018
TCGA-DK-A6B5	NA	NA		2	3	32	Basal	Basal	MS2b2.2	0	-1	1	0	1	1	-2	0	0	0	0	0	0.553	-0.57	0.379	-0.161	-0.181	-0.091	-0.319	-0.275	-0.064	0.118	0.452	0.464
TCGA-FD-A5BY	NA	NA		1	1	11	Basal	Basal	MS2b2.2	0	-1	0	0	0	0	0	0	0	0	-1	0	0.717	-0.73	0.473	-0.01	-0.362	-0.131	-0.328	-0.322	-0.156	0.181	0.456	0.56
TCGA-DK-A1A7	Non-papillary		1	2	3	31	Luminal	Luminal	MS1b	0	-1	0	0	0	0	0	0	2	0	0	0	-0.652	0.721	-0.382	0.173	0.458	0.167	0.381	0.266	0.058	-0.227	-0.353	-0.535
TCGA-C4-A0F0	Non-papillary		3	1	1	12	Basal	Basal	MS2b2.2	0	0	0	0	0	0	0	0	0	1	0	0	0.848	-0.839	0.683	-0.122	-0.51	-0.316	-0.504	-0.292	-0.04	0.326	0.367	0.74
TCGA-G2-A2EJ	Non-papillary		3	1	1	11	Basal	Basal	MS2b2.2	0	0	1	1	0	0	0	1	0	0	0	0	0.779	-0.755	0.663	-0.354	-0.291	-0.146	-0.265	-0.3	-0.154	0.001	0.496	0.645
TCGA-HQ-A2OF	NA	NA		2	2	21	Luminal	Luminal	MS2a1	0	0	0	0	1	1	-1	1	0	0	0	-1	-0.16	0.257	-0.185	-0.06	0.134	-0.06	0.087	0.336	0.174	-0.23	-0.219	-0.124
TCGA-BL-A3JM	NA		4	2	3	32	Basal	Basal	MS2b2.2	0	0	0	0	0	0	0	1	0	1	-1	0	0.659	-0.495	0.193	0.024	-0.296	-0.293	-0.401	0.113	0.251	0.23	-0.101	0.322
TCGA-FD-A3SP	Non-papillary		4	1	1	12	Basal	Basal	MS2b1	0	0	0	0	0	0	-1	1	0	0	0	1	0.855	-0.84	0.447	0.285	-0.672	-0.367	-0.677	-0.325	0.1	0.706	0.165	0.613
TCGA-DK-A3IM	Papillary		3	1	1	11	Basal	Basal	MS2b2.2	0	0	0	0	0	0	0	1	0	0	0	0	0.773	-0.674	0.615	-0.322	-0.241	-0.118	-0.189	-0.094	-0.087	-0.179	0.311	0.563
TCGA-FD-A43S	NA	NA		2	3	31	Basal	TP53-like	MS2b1	0	0	0	0	0	0	0	0	0	0	0	0	0.045	-0.166	-0.021	0.661	-0.241	0.219	-0.052	-0.4	-0.263	0.476	0.032	-0.142
TCGA-E7-A4XJ	NA	NA		2	2	22	Luminal	Luminal	MS1b	0	0	0	1	0	0	0	0	0	0	0	0	-0.659	0.72	-0.317	0.015	0.425	0.327	0.619	0.182	-0.139	-0.462	-0.35	-0.541
TCGA-FD-A3B8	Non-papillary		4	1	1	12	Basal	Basal	MS2b1	0	0	0	0	0	0	0	1	0	0	0	0	0.874	-0.853	0.566	0.329	-0.676	-0.318	-0.666	-0.461	-0.009	0.729	0.343	0.65
TCGA-E7-A5KF	NA	NA		2	2	22	Luminal	Luminal	MS1b	1	0	0	0	1	1	-2	0	0	0	0	0	-0.862	0.791	-0.464	-0.231	0.568	0.559	0.742	0.195	-0.323	-0.727	-0.178	-0.591
TCGA-HQ-A5ND	NA	NA		1	1	11	Basal	Basal	MS2b2.1	0	0	0	0	0	0	-2	1	0	0	0	0	0.539	-0.49	0.313	-0.287	-0.152	0.033	-0.042	-0.134	-0.226	-0.257	0.441	0.396
TCGA-DK-A6B1	NA	NA		2	2	21	Luminal	Luminal	MS2a1	0	0	0	0	0	0	0	0	0	0	-1	1	-0.802	0.811	-0.514	0.043	0.424	-0.064	0.206	0.663	0.345	-0.225	-0.558	-0.53
TCGA-K4-A5RH	NA	NA		1	1	12	Basal	Basal	MS2b1	0	0	0	0	0	0	0	1	0	1	0	0	0.848	-0.809	0.413	0.144	-0.572	-0.398	-0.647	-0.162	0.218	0.628	0.058	0.51
TCGA-BL-A5ZZ	NA	NA		1	1	12	Basal	Basal	MS2b1	0	0	0	0	0	0	-1	0	0	0	0	0	0.797	-0.766	0.54	0.252	-0.584	-0.347	-0.568	-0.307	0.077	0.634	0.155	0.537
TCGA-GC-A3OO	Non-papillary		2	2	3	31	Luminal	TP53-like	MS2b1	0	0	1	0	0	0	0	1	0	0	0	0	-0.109	0.118	-0.011	0.468	-0.148	-0.023	-0.052	-0.043	-0.058	0.174	0.073	-0.034
TCGA-DK-A2I6	Non-papillary		3	1	1	11	Basal	Basal	MS2b2.2	0	0	0	0	0	0	0	1	0	0	-1	1	0.715	-0.711	0.348	-0.234	-0.301	-0.277	-0.284	-0.057	0.081	0.027	0.239	0.501
TCGA-FD-A3SM	Papillary		2	2	3	31	Basal	TP53-like	MS2b1	1	0	0	0	1	1	-2	0	0	0	0	0	0.358	-0.423	-0.029	0.181	-0.173	-0.034	-0.123	-0.086	-0.025	0.173	0.058	0.078
TCGA-C4-A0F6	Non-papillary		2	2	3	31	Luminal	Luminal	MS1b	0	0	0	0	0	0	-2	0	2	0	0	0	-0.46	0.509	-0.192	0.026	0.276	0.066	0.172	0.162	0.039	-0.131	-0.072	-0.275
TCGA-BT-A3PH	Non-papillary		1	2	3	31	Luminal	Luminal	MS1b	0	0	0	1	0	0	0	1	0	1	0	0	-0.654	0.645	-0.277	-0.116	0.414	-0.041	0.338	0.318	0.179	-0.346	-0.196	-0.34
TCGA-BT-A20X	NA	NA		1	1	11	Basal	Basal	MS2b2.2	0	0	0	0	0	0	-1	1	0	0	0	0	0.84	-0.766	0.723	-0.128	-0.474	-0.145	-0.444	-0.436	-0.209	0.291	0.535	0.706
TCGA-E7-A3X6	Papillary		1	1	1	11	Basal	Basal	MS2b2.1	1	0	0	0	1	1	-2	0	0	0	-1	0	0.498	-0.562	0.263	-0.22	-0.113	0.173	-0.084	-0.517	-0.433	0.038	0.696	0.367
TCGA-DK-A1AG	Papillary		1	2	2	22	Luminal	Luminal	MS1a	1	0	1	0	1	1	-2	0	0	0	0	0	-0.603	0.311	-0.188	-0.143	0.273	0.601	0.494	-0.287	-0.585	-0.405	0.328	-0.265
TCGA-GC-A6I3	NA	NA		2	3	31	Luminal	Luminal	MS2a2	0	0	1	0	0	0	-2	1	0	0	0	1	-0.341	0.374	-0.199	0.118	0.159	-0.083	-0.062	0.15	0.248	0.187	-0.309	-0.223
TCGA-DK-A3IQ	Papillary	NA		2	3	31	Basal	TP53-like	MS2b1	0	0	0	0	0	0	0	0	0	0	0	0	0.71	-0.745	0.117	0.593	-0.468	-0.035	-0.254	-0.343	-0.126	0.585	-0.057	0.141
TCGA-BT-A2LB	Non-papillary		2	2	3	31	Luminal	TP53-like	MS2b1	0	0	0	1	0	0	0	1	0	0	0	0	-0.27	0.252	0.029	0.307	0.008	0.024	0.142	-0.077	-0.023	0.152	-0.158	-0.221
TCGA-DK-A6B0	NA	NA		2	2	22	Luminal	Luminal	MS1a	1	0	0	0	0	0	0	0	0	0	0	0	-0.787	0.647	-0.461	-0.081	0.411	0.752	0.694	-0.105	-0.562	-0.499	-0.106	-0.571
TCGA-E5-A4TZ	NA	NA		2	3	32	Luminal	Basal	MS2b2.1	0	-1	0	0	0	0	0	1	0	1	0	2	0.099	0.12	0.244	-0.358	0.149	0.02	0.041	0.086	-0.029	-0.361	0.209	0.175
TCGA-FD-A3SJ	Non-papillary		2	2	3	31	Luminal	TP53-like	MS2a1	0	0	1	0	0	0	0	1	0	0	0	2	-0.383	0.568	-0.343	0.422	0.168	-0.094	0.08	0.432	0.335	0.035	-0.552	-0.426
TCGA-GD-A2C5	Papillary		2	2	3	31	Luminal	Luminal	MS1b	0	-1	0	0	0	1	0	1	0	0	0	0	-0.536	0.535	-0.461	0.271	0.331	0.067	0.322	0.256	0.114	-0.069	-0.454	-0.515
TCGA-DK-A1AE	Non-papillary		3	1	1	11	Basal	Basal	MS2b2.2	0	0	0	0	0	0	-2	0	0	0	-1	0	0.664	-0.584	0.282	-0.075	-0.213	0	-0.168	-0.196	-0.171	-0.015	0.29	0.399
TCGA-FD-A3NA	Non-papillary		2	2	3	31	Luminal	Luminal	MS1b	0	0	1	0	0	0	-2	1	0	0	0	0	-0.439	0.593	0.121	-0.108	0.247	-0.004	0.236	0.076	0.012	-0.165	0.025	-0.198
TCGA-G2-A2EK	Non-papillary		2	2	3	31	Luminal	TP53-like	MS1b	0	0	0	0	0	0	0	0	2	0	0	0	-0.779	0.72	-0.452	0.383	0.351	0.406	0.463	-0.055	-0.285	-0.13	-0.152	-0.54
TCGA-FD-A3SO	Non-papillary		3	1	1	11	Basal	Basal	MS2b2.2	0	0	1	0	0	0	0	1	0	1	0	0	0.804	-0.774	0.644	-0.119	-0.499	-0.371	-0.596	-0.289	0.078	0.424	0.38	0.712
TCGA-FD-A5BX	NA	NA		1	1	12	Basal	TP53-like	MS2b1	0	0	1	1	0	0	-2	0	0	0	0	0	0.722	-0.822	0.299	0.311	-0.587	-0.22	-0.519	-0.355	0.002	0.664	0.167	0.407
TCGA-GV-A3QK	Non-papillary		1	2	2	21	Luminal	Luminal	MS1b	0	0	0	0	0	0	-2	0	0	0	0	0	-0.8	0.847	-0.487	-0.123	0.634	0.206	0.483	0.287	0.034	-0.323	-0.3	-0.62
TCGA-K4-A3WS	Non-papillary		2	2	3	31	Basal	TP53-like	MS2b1	0	0	1	0	0	0	-1	0	0	0	0	0	0.068	-0.115	-0.03	0.615	-0.216	-0.199	-0.217	-0.102	0.181	0.543	-0.192	-0.07
TCGA-DK-A3IU	NA		4	1	1	12	Basal	Basal	MS2b1	0	0	0	0	0	0	0	1	0	0	1	1	0.719	-0.742	0.44	0.322	-0.511	-0.371	-0.646	-0.294	0.167	0.686	0.157	0.526
TCGA-CF-A1HR	Non-papillary		2	2	3	31	Luminal	Luminal	MS1b	0	0	0	0	0	0	-2	0	0	0	0	0	-0.68	0.585	-0.399	0.209	0.321	0.271	0.37	0.057	-0.096	-0.147	-0.194	-0.486
TCGA-KQ-A41Q	NA	NA		2	2	21	Luminal	Luminal	MS2a1	0	0	0	0	0	0	0	0	0	1	-1	1	-0.712	0.814	-0.412	-0.12	0.525	0.019	0.428	0.525	0.221	-0.455	-0.481	-0.488
TCGA-FD-A6TI	NA	NA		2	3	31	Luminal	TP53-like	MS1a	0	0	0	0	0	0	0	0	0	0	0	0	-0.536	0.542	-0.308	0.335	0.235	0.296	0.265	0.048	-0.138	0	-0.291	-0.453
TCGA-BL-A13I	Non-papillary		4	1	1	12	Basal	Basal	MS2b1	0	0	0	0	0	0	-1	0	0	0	0	0	0.735	-0.757	0.32	0.081	-0.498	-0.384	-0.558	-0.164	0.206	0.555	0.084	0.459
TCGA-CF-A47X	NA	NA		2	2	21	Luminal	Luminal	MS1b	0	0	0	1	0	0	0	0	0	0	0	1	-0.79	0.918	-0.547	-0.017	0.595	0.514	0.673	0.173	-0.248	-0.44	-0.368	-0.722
TCGA-E5-A2PC	Non-papillary		1	1	1	11	Basal	Basal	MS2b2.1	1	0	1	0	1	1	-2	1	0	0	-1	-1	0.518	-0.495	0.148	-0.299	-0.049	0.138	-0.009	-0.264	-0.304	-0.186	0.549	0.295
TCGA-R3-A69X	NA	NA		2	3	31	Luminal	TP53-like	MS2b1	0	0	0	0	0	0	-1	0	0	0	0	0	-0.209	0.042	-0.182	0.442	-0.074	-0.116	-0.106	-0.052	0.127	0.379	-0.143	-0.14
TCGA-CU-A3QU	Non-papillary		1	2	2	21	Luminal	Luminal	MS2a1	0	0	0	0	0	1	1	0	0	0	-2	1	-0.783	0.871	-0.42	-0.192	0.542	-0.123	0.241	0.751	0.413	-0.41	-0.484	-0.473
TCGA-DK-A3X2	Papillary		1	2	3	32	Basal	Basal	MS2a1	0	-1	0	0	0	0	0	1	0	1	-2	-1	0.248	-0.208	0.176	-0.321	0.084	-0.016	0.038	0.227	0.037	-0.426	0.133	0.151
TCGA-GD-A3OP	Papillary		2	2	3	31	Luminal	TP53-like	MS1b	0	0	1	0	0	0	0	0	0	0	0	0	-0.739	0.828	-0.526	0.434	0.3	0.327	0.374	0.153	-0.097	-0.012	-0.498	-0.646
TCGA-E7-A7DU	NA	NA		2	2	22	Luminal	Luminal	MS1b	1	0	1	0	1	1	-2	1	0	0	0	0	-0.821	0.794	-0.486	-0.067	0.487	0.617	0.699	0.015	-0.424	-0.556	-0.101	-0.583
TCGA-E7-A7DV	NA	NA		1	1	12	Basal	Basal	MS2b1	1	0	0	0	0	0	0	1	0	0	0	2	0.647	-0.687	0.368	-0.056	-0.416	-0.452	-0.608	-0.149	0.277	0.556	0.178	0.489
TCGA-BT-A20Q	Non-papillary		2	2	3	31	Basal	TP53-like	MS2b1	0	0	1	0	1	1	-2	0	0	0	0	0	0.246	-0.459	-0.071	0.346	-0.167	0.258	0.05	-0.453	-0.361	0.291	0.199	-0.065
TCGA-CF-A47W	NA	NA		2	2	22	Luminal	Luminal	MS1b	1	0	0	0	0	0	0	0	0	0	0	0	-0.727	0.662	-0.494	0.004	0.379	0.677	0.691	0	-0.447	-0.505	-0.224	-0.611
TCGA-CU-A0YR	Non-papillary		2	2	3	31	Luminal	Luminal	MS2a2	0	0	0	0	0	0	0	0	0	0	0	0	-0.273	0.358	-0.028	-0.097	0.183	-0.354	-0.073	0.21	0.422	0.143	-0.24	-0.095
TCGA-BT-A20O	Non-papillary		4	1	1	12	Basal	Basal	MS2b1	0	0	1	0	0	0	0	1	0	0	0	0	0.812	-0.787	0.405	0.082	-0.558	-0.401	-0.652	-0.218	0.192	0.641	0.143	0.537
TCGA-DK-A6AW	NA	NA		2	2	21	Luminal	TP53-like	MS1a	0	0	1	1	0	0	0	0	0	0	0	0	-0.179	0.059	-0.13	0.098	0.062	0.191	0.135	0.121	-0.086	-0.113	-0.138	-0.222
TCGA-DK-A1A3	Papillary		2	2	3	31	Luminal	TP53-like	MS2a2	0	0	0	0	0	0	-1	1	0	0	0	0	-0.152	0.205	-0.246	0.246	0.126	-0.269	-0.193	0.24	0.384	0.283	-0.323	-0.134
TCGA-K4-A4AB	NA	NA		2	3	31	Basal	TP53-like	MS1a	0	0	1	0	0	0	-2	1	0	0	0	0	0.195	-0.374	0.07	0.399	-0.196	0.391	0.047	-0.473	-0.499	0.239	0.288	-0.054
TCGA-UY-A78K	NA	NA		2	3	31	Luminal	Luminal	MS2a2	0	0	0	0	0	0	0	1	0	0	-1	2	-0.485	0.686	-0.278	0.018	0.229	-0.353	-0.229	0.51	0.576	0.172	-0.435	-0.205
TCGA-K4-A5RI	NA	NA		1	1	11	Basal	Basal	MS2b2.1	0	0	0	0	0	0	-2	1	0	0	0	0	0.695	-0.665	0.376	0.043	-0.348	-0.043	-0.319	-0.358	-0.238	0.145	0.577	0.543
TCGA-GU-A766	NA	NA		1	1	11	Basal	Basal	MS2b2.2	0	0	0	0	0	0	0	1	0	0	0	0	0.772	-0.761	0.692	0.019	-0.53	-0.317	-0.587	-0.419	0.002	0.525	0.393	0.697
TCGA-FD-A62N	NA	NA		1	1	12	Basal	Basal	MS2b1	0	0	0	0	0	0	0	1	0	1	0	0	0.816	-0.808	0.61	0.115	-0.63	-0.363	-0.695	-0.384	0.074	0.682	0.292	0.673
TCGA-FD-A3SL	Non-papillary		2	2	3	31	Luminal	TP53-like	MS2b1	0	0	0	0	0	0	0	1	0	1	0	0	-0.256	0.338	-0.252	0.512	-0.017	-0.185	-0.147	0.272	0.318	0.357	-0.467	-0.271
TCGA-CF-A27C	Papillary		1	2	2	22	Luminal	Luminal	MS1b	1	1	0	0	1	1	-2	0	0	0	0	0	-0.792	0.802	-0.479	-0.218	0.562	0.586	0.752	0.106	-0.389	-0.67	-0.114	-0.592
TCGA-DK-A3X1	Non-papillary		2	2	2	21	Luminal	Luminal	MS1b	0	0	1	0	0	0	0	1	0	1	0	0	-0.195	0.325	-0.211	-0.055	0.327	0.022	0.267	0.249	0.111	-0.25	-0.256	-0.285

TCGA-BT-A20R	Non-papillary		2	2	3	31	Basal	TP53-like	MS2b1	0	0	1	0	0	0	0	1	0	1	0	2	0.257	-0.209	-0.049	0.333	-0.16	-0.318	-0.351	0.165	0.351	0.419	-0.286	0.045
TCGA-GV-A3QG	Papillary		4	1	1	12	Basal	TP53-like	MS2b1	0	0	0	0	0	0	0	0	0	0	0	0	0.344	-0.437	0.112	0.289	-0.309	-0.451	-0.484	0.02	0.421	0.6	-0.213	0.189
TCGA-FD-A3SR	Non-papillary		2	2	3	31	Basal	TP53-like	MS2a2	0	0	0	0	0	0	-1	1	0	0	0	1	0.106	0.001	-0.169	0.296	-0.023	-0.314	-0.353	0.264	0.429	0.375	-0.332	-0.033
TCGA-E7-A541	NA	NA		2	2	21	Luminal	Luminal	MS2a2	0	0	0	0	0	0	0	0	1	0	-2	0	-0.452	0.522	-0.18	-0.157	0.178	-0.11	-0.052	0.176	0.204	0.007	-0.089	-0.09
TCGA-GV-A3QH	Non-papillary		1	2	2	21	Luminal	Luminal	MS2a1	0	0	0	0	0	0	-2	1	0	0	0	2	-0.831	0.9	-0.409	-0.204	0.553	0.017	0.358	0.658	0.315	-0.523	-0.445	-0.517
TCGA-G2-A3IB	Non-papillary		3	1	1	11	Basal	Basal	MS2b2.2	0	-1	0	0	1	0	-1	1	0	0	0	0	0.697	-0.638	0.589	-0.319	-0.209	-0.001	-0.114	-0.257	-0.241	-0.159	0.487	0.509
TCGA-BT-A20N	Non-papillary		1	2	3	32	Basal	Luminal	MS1b	0	0	0	0	0	0	-1	1	0	0	-1	0	0.108	0.082	0.057	-0.31	0.19	0.08	0.176	0.129	-0.093	-0.374	0.056	0.031
TCGA-E7-A6ME	NA	NA		2	3	32	Luminal	Luminal	MS2a1	0	0	0	0	0	0	0	1	0	1	-1	0	-0.606	0.758	-0.291	0.077	0.369	-0.088	0.109	0.55	0.342	-0.171	-0.406	-0.408
TCGA-DK-A3IT	Non-papillary		2	2	3	31	Luminal	TP53-like	MS2a2	0	-1	0	0	0	0	0	0	0	0	0	0	-0.322	0.227	-0.141	0.256	0.116	-0.289	-0.034	0.215	0.347	0.122	-0.246	-0.131
TCGA-UY-A78L	NA	NA		1	1	11	Basal	Basal	MS2b2.2	0	0	0	0	0	0	0	1	0	1	0	0	0.699	-0.697	0.485	-0.081	-0.294	-0.368	-0.433	-0.055	0.167	0.191	0.268	0.535
TCGA-CF-A47V	NA	NA		2	2	22	Luminal	Luminal	MS1b	1	2	0	0	0	1	0	0	0	0	0	0	-0.727	0.511	-0.391	0.154	0.232	0.534	0.598	-0.133	-0.388	-0.251	-0.219	-0.553
TCGA-FJ-A3ZF	Papillary		1	2	2	21	Luminal	Luminal	MS2a1	1	0	0	0	0	0	0	1	0	0	0	2	-0.821	0.917	-0.518	-0.094	0.599	-0.037	0.343	0.703	0.378	-0.42	-0.651	-0.555
TCGA-FD-A6TH	NA	NA		1	3	31	Basal	Basal	MS2a2	0	0	0	0	0	0	0	0	0	1	0	0	0.577	-0.541	0.202	0.08	-0.238	-0.385	-0.401	0.118	0.337	0.248	-0.126	0.335
TCGA-FD-A3N5	Non-papillary		3	1	1	11	Basal	Basal	MS2b2.2	0	0	0	0	0	0	1	1	0	1	-2	2	0.699	-0.663	0.669	-0.169	-0.333	-0.26	-0.374	-0.195	-0.032	0.074	0.422	0.676
TCGA-K4-A54R	NA	NA		2	3	31	Basal	Basal	MS2b1	0	-1	1	0	0	0	0	1	0	1	-1	1	0.34	-0.478	0.103	-0.068	-0.119	-0.29	-0.262	-0.064	0.209	0.311	-0.004	0.194
TCGA-GV-A3QI	Papillary		1	2	3	32	Luminal	Luminal	MS2a1	0	0	0	0	0	0	0	0	0	0	-2	0	-0.137	0.176	-0.201	-0.066	0.181	0.061	0.216	0.438	0.087	-0.381	-0.292	-0.238
TCGA-HQ-A2OE	Papillary		1	2	2	21	Luminal	Luminal	MS1b	0	0	0	0	1	1	-1	0	1	0	-1	1	-0.623	0.464	-0.31	-0.178	0.403	0.02	0.345	0.321	0.154	-0.384	-0.263	-0.33
TCGA-G2-A3IE	Non-papillary		1	2	2	21	Luminal	Luminal	MS1b	0	0	0	0	0	0	-1	1	0	0	0	0	-0.816	0.863	-0.563	-0.038	0.602	0.368	0.626	0.273	-0.085	-0.448	-0.432	-0.685
TCGA-H4-A2HO	Papillary		1	2	2	21	Luminal	Luminal	MS2a1	0	0	1	0	0	0	0	0	1	0	0	0	-0.836	0.839	-0.553	0.226	0.426	0.069	0.251	0.37	0.199	-0.028	-0.552	-0.588
TCGA-KQ-A41S	NA	NA		2	3	31	Luminal	TP53-like	MS2b1	0	0	0	0	0	0	0	1	0	0	-1	0	-0.166	0.234	-0.208	0.35	-0.043	-0.15	-0.097	0.21	0.21	0.233	-0.266	-0.186
TCGA-DK-A3WW	Papillary		3	1	1	11	Basal	Basal	MS2b2.2	0	0	1	0	0	0	-2	0	0	0	0	0	0.767	-0.782	0.691	-0.309	-0.457	-0.205	-0.484	-0.427	-0.127	0.324	0.521	0.718
TCGA-FD-A5BZ	NA	NA		2	3	31	Luminal	TP53-like	MS2b1	0	0	0	0	1	1	-2	0	2	0	0	0	-0.142	0.225	-0.023	0.521	-0.087	-0.08	0.079	-0.02	0.05	0.252	-0.263	-0.2
TCGA-DK-A3IL	Non-papillary		2	2	3	31	Luminal	TP53-like	MS2a1	0	0	0	0	0	0	0	1	0	1	0	0	-0.363	0.654	-0.392	0.299	0.274	-0.026	0.231	0.404	0.253	-0.172	-0.455	-0.425
TCGA-DK-A6B2	NA	NA		2	3	31	Luminal	TP53-like	MS2a2	0	0	1	0	0	0	0	0	1	0	0	0	-0.191	0.513	-0.301	0.288	0.18	-0.152	0.001	0.194	0.321	0.258	-0.503	-0.323
TCGA-GV-A3JZ	Non-papillary		2	2	3	31	Luminal	Luminal	MS2a2	0	0	0	0	0	0	1	1	-1	0	0	2	-0.13	0.283	-0.217	0.038	0.133	-0.348	-0.136	0.461	0.495	0.078	-0.421	-0.136
TCGA-FD-A6TD	NA	NA		1	1	11	Basal	Basal	MS2b2.2	0	0	1	0	0	0	0	1	0	1	0	0	0.744	-0.774	0.632	-0.058	-0.516	-0.299	-0.564	-0.411	-0.037	0.47	0.493	0.693
TCGA-FD-A43X	NA	NA		2	2	22	Luminal	Luminal	MS1b	1	0	0	0	1	1	-2	0	0	0	0	0	-0.798	0.685	-0.463	-0.071	0.459	0.659	0.754	0.006	-0.474	-0.575	-0.16	-0.606
TCGA-BT-A42C	Papillary		1	2	2	21	Luminal	Luminal	MS1b	1	0	0	0	0	1	-2	0	2	0	-2	0	-0.749	0.72	-0.249	-0.165	0.475	0.241	0.537	0.198	-0.122	-0.553	-0.039	-0.397
TCGA-GV-A3QF	Papillary		1	2	2	21	Luminal	Luminal	MS2a1	0	0	0	0	0	0	-2	0	0	0	0	0	-0.826	0.866	-0.468	-0.144	0.561	0.08	0.457	0.46	0.178	-0.502	-0.289	-0.526
TCGA-FJ-A3ZE	Non-papillary		1	2	2	22	Luminal	Luminal	MS1b	1	0	0	0	0	0	-2	1	0	0	0	0	-0.313	0.389	-0.236	-0.231	0.401	0.288	0.417	0.252	-0.128	-0.574	-0.073	-0.298
TCGA-K4-A6FZ	NA	NA		1	1	11	Basal	Basal	MS2b2.1	0	0	0	0	0	0	0	1	0	1	0	2	0.22	-0.38	0.431	-0.191	-0.029	0.246	0.107	-0.364	-0.43	-0.121	0.502	0.168
TCGA-BT-A42E	NA	NA		1	1	11	Basal	Basal	MS2b2.2	0	0	0	0	0	0	-2	0	0	0	0	0	0.854	-0.832	0.701	-0.248	-0.433	-0.289	-0.504	-0.41	-0.044	0.334	0.474	0.757
TCGA-DK-A6B6	NA	NA		2	2	21	Luminal	Luminal	MS1b	0	0	0	0	0	0	0	1	0	0	0	0	-0.809	0.852	-0.504	-0.179	0.582	0.277	0.434	0.349	0.035	-0.325	-0.396	-0.643
TCGA-GU-A762	NA	NA		1	1	12	Basal	Basal	MS2b2.2	0	0	0	0	0	0	0	1	0	0	0	0	0.807	-0.809	0.617	-0.057	-0.617	-0.328	-0.61	-0.351	0.037	0.526	0.289	0.695
TCGA-G2-A2ES	Non-papillary		3	1	1	11	Basal	Basal	MS2b2.2	0	0	0	0	0	0	0	1	0	0	-1	0	0.796	-0.718	0.668	-0.23	-0.396	-0.153	-0.388	-0.461	-0.203	0.238	0.624	0.679
TCGA-CF-A47T	NA	NA		2	2	22	Luminal	Luminal	MS1b	0	0	0	1	0	0	-2	0	0	0	0	0	-0.748	0.876	-0.38	-0.047	0.477	0.47	0.696	0.065	-0.338	-0.564	-0.169	-0.502
TCGA-DK-A1AB	Non-papillary		3	1	1	11	Basal	Basal	MS2b2.2	0	0	1	0	0	0	0	1	0	0	0	0	0.859	-0.756	0.527	0.213	-0.513	-0.033	-0.415	-0.547	-0.306	0.437	0.491	0.584
TCGA-UY-A78N	NA	NA		2	3	32	Luminal	Luminal	MS2a1	1	0	0	0	0	0	0	1	0	0	-2	1	-0.515	0.711	-0.328	0.141	0.256	0.031	0.273	0.41	0.144	-0.313	-0.381	-0.34
TCGA-DK-A3IS	Papillary		1	2	2	22	Luminal	Luminal	MS1b	1	2	0	0	1	1	-1	1	0	0	0	0	-0.658	0.693	-0.305	-0.281	0.46	0.321	0.491	0.103	-0.236	-0.581	0.167	-0.306
TCGA-FD-A6TF	NA	NA		2	3	31	Basal	TP53-like	MS2b1	0	0	0	0	0	0	0	1	0	1	0	0	0.685	-0.677	0.272	0.354	-0.393	-0.4	-0.55	0.013	0.296	0.508	-0.061	0.363
TCGA-BT-A20P	Non-papillary		1	2	2	22	Luminal	Luminal	MS1b	0	2	0	0	0	1	-2	0	1	0	0	0	-0.484	0.45	-0.287	-0.073	0.194	0.401	0.452	0.009	-0.294	-0.431	0.066	-0.337
TCGA-E5-A4U1	NA	NA		2	2	21	Luminal	Luminal	MS1b	0	1	0	0	1	1	-1	1	0	0	0	0	-0.735	0.789	-0.441	-0.016	0.467	0.276	0.57	0.353	-0.016	-0.551	-0.337	-0.567

Supplementary table 7
Gene signatures used in Figures 1-2, Gene IDs are according to TCGA nomenclature
>Blaveri_SCCdown
CLDN4|1364
EPCAM|4072
ERBB2|2064
GATA3|2625
GPD1L|23171
S100P|6286
SLC9A1|6548

>Blaveri_SCCup
APBA1|320
CA2|760
CAD|790
COL17A1|1308
DSC2|1824
ELL2|22936
HBEGF|1839
IL13RA2|3598
KRT14|3861
KRT16|3868
KRT5|3852
LGALS7|3963
LOC100133331|100133331
MAF|4094
MMP3|4314
MXD1|4084
PI3|5266
PTHLH|5744
PURA|5813
S100A7|6278
S100A8|6279
SFN|2810

>Lund_QTC1_TCell
ABI3|51225
ACAP1|9744
ADAMDEC1|27299
ADAP2|55803
AIF1|199
ALOX5AP|241
AMICA1|120425
ANXA6|309
AOAH|313
AP1S2|8905
APOE|348
ARHGAP15|55843
ARHGAP25|9938
ARHGAP9|64333
ATP8B4|79895
BATF3|55509
BIN1|274
BTK|695
C1orf162|128346
C1orf54|79630
C1QA|712
C1QB|713
C1QC|714
C1S|716
C3AR1|719
CCL2|6347
CCL5|6352
CD14|929
CD163|9332
CD2|914
CD209|30835
CD247|919
CD300LF|146722
CD37|951
CD38|952
CD3D|915
CD48|962
CD52|1043
CD53|963
CD6|923
CD7|924
CD74|972
CD86|942
CLEC4A|50856

CORO1A|11151
CPVL|54504
CSF1R|1436
CST7|8530
CTLA4|1493
CTSK|1513
CYBB|1536
CYTH4|27128
CYTIP|9595
DOCK10|55619
DOCK11|139818
DOCK2|1794
EMP3|2014
ENTPD1|953
EVI2A|2123
EVI2B|2124
FAM65B|9750
FCER1G|2207
FCN1|2219
FERMT3|83706
FGL2|10875
FPR3|2359
FYB|2533
FYN|2534
GIMAP1|170575
GIMAP4|55303
GIMAP5|55340
GIMAP7|168537
GLIPR2|152007
GPR183|1880
GPR65|8477
GYPC|2995
GZMA|3001
GZMB|3002
GZMH|2999
GZMK|3003
HAVCR2|84868
HCST|10870
HLA-DMB|3109
HLA-DPA1|3113
HLA-DPB1|3115
HLA-DQA1|3117
HLA-DRA|3122
ICOS|29851
IFFO1|25900
IFI30|10437
IFITM1|8519
IGSF6|10261
IKZF1|10320
IL10RA|3587
IL18BP|10068
IL18RAP|8807
IL2RA|3559
IL2RB|3560
IL7R|3575
IQGAP2|10788
IRF8|3394
ISG20|3669
ITGAL|3683
ITGAM|3684
ITGB2|3689
ITK|3702
KIAA1949|170954
LAPTM5|7805
LAT2|7462
LILRB2|10288
LILRB4|11006
LILRB5|10990
LOC100133331|100133331
LST1|7940
LTA|4049
LTB|4050
LY86|9450
LY96|23643
LYL1|4066
MAP4K1|11184
MATK|4145
MFNG|4242
MMP9|4318
MS4A4A|51338
MS4A6A|64231
MSN|4478

MYO1G|64005
NCF1|653361
NCF1C|654817
NCKAP1L|3071
NKG7|4818
P2RY13|53829
PARVG|64098
PDE4B|5142
PECAM1|5175
PIK3AP1|118788
PIM2|11040
PLA2G4C|8605
PLEK|5341
PLEKHO1|51177
PLEKHO2|80301
PPP1R16B|26051
PRF1|5551
PTPN22|26191
PTPN7|5778
PTPRCAP|5790
PVRIG|79037
PYHIN1|149628
RASAL3|64926
RASSF4|83937
RGS1|5996
RGS18|64407
RGS2|5997
RNASE6|6039
RUNX3|864
SAMSN1|64092
SASH3|54440
SH2B3|10019
SIGLEC10|89790
SLA|6503
SLAMF1|6504
SLAMF6|114836
SLAMF8|56833
SLC7A7|9056
SLCO2B1|11309
SPI1|6688
SRGN|5552
ST6GAL1|6480
STAT4|6775
SUSD3|203328
TBC1D10C|374403
TBXAS1|6916
TLR8|51311
TMEM176A|55365
TNFRSF1B|7133
TNFRSF4|7293
TNFSF13B|10673
TOX2|84969
TRAF3IP3|80342
TRPV2|51393
TSPAN4|7106
TYROBP|7305
WAS|7454
WIPF1|7456

>Lund_QTC14_UrothelialDifferentiation
ACER2|340485
BCAS1|8537
C10orf116|10974
CAPN5|726
CYB5A|1528
CYP4B1|1580
CYP4F12|66002
CYP4F22|126410
DHRS2|10202
FAM174B|400451
FAM3B|54097
GGT6|124975
HMGCS2|3158
HPGD|3248
PKHD1|5314
PPARG|5468
PPFIBP2|8495
PSCA|8000
SNCG|6623
TBX3|6926
TRAK1|22906

UPK1A|11045
VSIG2|23584

>Lund_QTC16_FGFR3signature
C16orf74|404550
C3orf54|389119
CAPNS2|84290
CLCA4|22802
DUOX1|53905
DUOXA1|90527
FGFR3|2261
IRS1|3667
LOC100133331|100133331
PLCH2|9651
PTPN13|5783
SEMA4B|10509
SLC2A9|56606
SMAD3|4088
SSH3|54961
SYTL1|84958
TMPRSS4|56649
TP63|8626
WNT7B|7477
ZNF385A|25946

>Lund_QTC17_MyeloidCell
AQP9|366
BCL2A1|597
C19orf59|199675
CCL3|6348
CCR1|1230
CSF3R|1441
CXCL2|2920
FCGR3B|2215
ITGAX|3687
MMP25|64386
MNDA|4332
OSM|5008
PHACTR1|221692
PROK2|60675
SOD2|6648
TAGAP|117289
VNN2|8875

>Lund_QTC3_Proliferation
ANLN|54443
ASF1B|55723
ASPM|259266
ATAD2|29028
AURKA|6790
AURKB|9212
BIRC5|332
BUB1|699
BUB1B|701
C12orf48|55010
C15orf23|90417
C16orf59|80178
C17orf53|78995
C1orf112|55732
C1orf135|79000
CCNA2|890
CCNB1|891
CCNB2|9133
CCNE2|9134
CDC20|991
CDC25A|993
CDC25C|995
CDC45|8318
CDCA2|157313
CDCA3|83461
CDCA5|113130
CDCA8|55143
CDK1|983
CDKN3|1033
CDT1|81620
CENPA|1058
CENPE|1062
CENPF|1063
CENPL|91687

CENPM|79019
CENPN|55839
CENPW|387103
CEP55|55165
CHAF1B|8208
CHEK1|1111
CKAP2L|150468
CKS1B|1163
DEPDC1|55635
DEPDC1B|55789
DLGAP5|9787
DONSON|29980
DTL|51514
E2F2|1870
ECT2|1894
EXO1|9156
FAM64A|54478
FAM83D|81610
FANCI|55215
FEN1|2237
FOXM1|2305
GINS2|51659
H2AFZ|3015
HJURP|55355
HMGB2|3148
HMMR|3161
KIAA1524|57650
KIF11|3832
KIF14|9928
KIF15|56992
KIF18A|81930
KIF20A|10112
KIF20B|9585
KIF23|9493
KIF2C|11004
KIF4A|24137
KIFC1|3833
KPNA2|3838
LMNB1|4001
LOC100133331|100133331
MCM10|55388
MCM2|4171
MCM6|4175
MCM7|4176
MELK|9833
MND1|84057
NCAPG|64151
NCAPG2|54892
NDC80|10403
NEK2|4751
NUF2|83540
NUSAP1|51203
OIP5|11339
PKMYT1|9088
PLK4|10733
POC1A|25886
POLA2|23649
POLE2|5427
POLQ|10721
PRC1|9055
PRR11|55771
PTTG1|9232
PTTG3P|26255
RACGAP1|29127
RAD51AP1|10635
RFC3|5983
RFC4|5984
RNASEH2A|10535
RRM2|6241
SGOL1|151648
SGOL2|151246
SKA1|220134
SKA3|221150
SPC25|57405
STIL|6491
TK1|7083
TOP2A|7153
TPX2|22974
TRIP13|9319
TTK|7272
TYMS|7298
UBE2C|11065

UBE2T|29089
UHRF1|29128
ZWINT|11130

>Lund_QTC4_ECM
ACTA2|59
ACTG2|72
AEBP1|165
ANGPTL2|23452
ARHGAP24|83478
BNC2|54796
C1QTNF1|114897
C1R|715
CALD1|800
CD248|57124
CD93|22918
CDH11|1009
CHN1|1123
CLIP3|25999
COL15A1|1306
COL1A1|1277
COL1A2|1278
COL3A1|1281
COL5A1|1289
COL5A2|1290
COL6A1|1291
COL6A2|1292
COL6A3|1293
COL8A1|1295
COLEC12|81035
COX7A1|1346
CPXM1|56265
CRISPLD2|83716
CTHRC1|115908
DACT3|147906
DCN|1634
DDR2|4921
DPYSL2|1808
ECM2|1842
EDNRA|1909
EMILIN1|11117
FAM20C|56975
FERMT2|10979
FILIP1L|11259
FSTL1|11167
GEM|2669
GLT8D2|83468
GPR124|25960
GUCY1A3|2982
HEPH|9843
IGDCC4|57722
ISLR|3671
JAM3|83700
KCNMB1|3779
LAMA4|3910
LGALS1|3956
LHFP|10186
LMCD1|29995
LOC100133331|100133331
LRRC32|2615
LRRC33|375387
LUM|4060
MFGE8|4240
MSC|9242
MSRB3|253827
MYADM|91663
MYL9|10398
MYLK|4638
OLFML1|283298
OLFML2B|25903
PAPSS2|9060
PCOLCE|5118
PCSK5|5125
PDGFRB|5159
PDLIM3|27295
PDPN|10630
POSTN|10631
PRKCDBP|112464
PRRX1|5396
PTRF|284119
PXDN|7837

RAB3IL1|5866
RARRES2|5919
RCN3|57333
SCARF2|91179
SERPINF1|5176
SGIP1|84251
SPARC|6678
SPON2|10417
SSC5D|284297
STX2|2054
SULF1|23213
SYT11|23208
TAGLN|6876
TGFB3|7043
THY1|7070
TIMP2|7077
VASN|114990
VIM|7431
ZEB2|9839

>MDA_classifier
A2M|2
AADAT|51166
ABCA12|26154
ABCA3|21
ABCA8|10351
ABCC1|4363
ABCC4|10257
ABCD3|5825
ABHD12|26090
ABHD14A|25864
ABI3BP|25890
ABL1|25
ACAA1|30
ACACB|32
ACADM|34
ACER2|340485
ACER3|55331
ACOT4|122970
ACOT7|11332
ACOT9|23597
ACOX1|51
ACOX2|8309
ACOXL|55289
ACP1|52
ACP5|54
ACP6|51205
ACPL2|92370
ACSBG1|23205
ACSF2|80221
ACSL1|2180
ACSL5|51703
ACSM3|6296
ACSS1|84532
ACTA2|59
ACTC1|70
ACTG2|72
ACTL6A|86
ACTN1|87
ACVR2A|92
ACVRL1|94
ADA|100
ADAM10|102
ADAM19|8728
ADAM23|8745
ADAMTS1|9510
ADAMTS9|56999
ADAP2|55803
ADARB1|104
ADCK2|90956
ADCY1|107
ADCY4|196883
ADCY6|112
ADH1A|124
ADH1C|126
ADHFE1|137872
ADK|132
ADORA2B|136
ADSS|159
AEBP1|165
AFAP1|60312

AFF3|3899
AGFG1|3267
AGMAT|79814
AGPAT4|56895
AGPAT9|84803
AGPS|8540
AGR2|10551
AGTRAP|57085
AHNAK2|113146
AHR|196
AIF1|199
AIM2|9447
AK1|203
AK3|50808
AK3L1|205
AKIRIN1|79647
AKIRIN2|55122
AKR1C2|1646
AKR1C3|8644
AKTIP|64400
ALDH1A2|8854
ALDH1B1|219
ALDH2|217
ALDH4A1|8659
ALDH5A1|7915
ALDH7A1|501
ALDOA|226
ALOX12B|242
ALOX5|240
ALOX5AP|241
ALPK2|115701
AMACR|23600
AMOT|154796
AMT|275
ANGPTL2|23452
ANKDD1A|348094
ANKRD29|147463
ANKRD35|148741
ANKRD46|157567
ANKRD6|22881
ANKS1A|23294
ANLN|54443
ANPEP|290
ANTXR1|84168
ANTXR2|118429
ANXA1|301
ANXA2|302
ANXA2P1|303
ANXA2P3|305
ANXA4|307
ANXA5|308
ANXA6|309
ANXA8L1|728113
ANXA9|8416
AOC3|8639
AP1S1|1174
AP1S2|8905
APCDD1L|164284
APEX2|27301
APLNR|187
APOBEC3G|60489
APOD|347
APOL4|80832
AQP1|358
AQP9|366
ARHGAP1|392
ARHGAP21|57584
ARHGAP22|58504
ARHGAP23|57636
ARHGAP32|9743
ARHGAP9|64333
ARHGEF1|9138
ARHGEF10|9639
ARHGEF17|9828
ARHGEF2|9181
ARHGEF4|50649
ARL14|80117
ARL6IP1|23204
ARL6IP5|10550
ARL6IP6|151188
ARNTL|406
ASAM|79827

ASAP1|50807
ASAP3|55616
ASB5|140458
ASCC2|84164
ASCC3|10973
ASPM|259266
ASTN2|23245
ATAD2|29028
ATF7IP2|80063
ATG9A|79065
ATL2|64225
ATL3|25923
ATP10B|23120
ATP1A1|476
ATP1A2|477
ATP1B3|483
ATP2A2|488
ATP2B4|493
ATP2C1|27032
ATP5C1|509
ATP6AP1|537
ATP6V0E2|155066
ATP6V1B2|526
ATP8B1|5205
ATP8B2|57198
ATP8B4|79895
ATR|545
ATXN1|6310
AURKA|6790
AURKB|9212
AXIN2|8313
AXL|558
B3GALNT1|8706
B4GALT3|8703
B4GALT4|8702
BAG3|9531
BAMBI|25805
BANK1|55024
BATF|10538
BATF2|116071
BCAS1|8537
BCAT2|587
BCKDHB|594
BCL11B|64919
BCL3|602
BEND5|79656
BEX2|84707
BEX4|56271
BGN|633
BHLHE40|8553
BHLHE41|79365
BHMT|635
BICD2|23299
BIN1|274
BMP1|649
BMP3|651
BMP4|652
BNC1|646
BNC2|54796
BPGM|669
BPHL|670
BRAF|673
BRIX1|55299
BTBD1|53339
BTBD16|118663
BTG2|7832
BTG3|10950
BUB1|699
BZW1|9689
BZW2|28969
C10orf11|83938
C10orf116|10974
C10orf35|219738
C10orf58|84293
C11orf1|64776
C11orf52|91894
C11orf54|28970
C11orf82|220042
C13orf34|79866
C14orf129|51527
C14orf149|112849
C15orf17|57184

C15orf23|90417
C17orf28|283987
C17orf58|284018
C17orf82|388407
C18orf19|125228
C18orf34|374864
C19orf2|8725
C19orf46|163183
C19orf56|51398
C19orf59|199675
C1orf116|79098
C1orf162|128346
C1orf198|84886
C1orf43|25912
C1orf53|388722
C1orf54|79630
C1QA|712
C1QB|713
C1QC|714
C1QTNF1|114897
C1R|715
C1S|716
C20orf24|55969
C21orf2|755
C21orf34|388815
C21orf63|59271
C21orf81|391267
C22orf32|91689
C22orf36|388886
C2CD2|25966
C2orf15|150590
C2orf18|54978
C2orf40|84417
C2orf55|343990
C2orf64|493753
C3AR1|719
C3orf10|55845
C3orf19|51244
C3orf23|285343
C3orf38|285237
C3orf57|165679
C3orf58|205428
C4orf19|55286
C4orf34|201895
C5AR1|728
C5orf13|9315
C5orf39|389289
C5orf4|10826
C6orf141|135398
C6orf150|115004
C7|730
C7orf10|79783
C7orf23|79161
C7orf28A|51622
C7orf28B|221960
C7orf41|222166
C7orf46|340277
C7orf55|154791
C7orf58|79974
C7orf68|29923
C8orf47|203111
C8orf55|51337
C8orf84|157869
C9orf116|138162
C9orf140|89958
C9orf21|195827
C9orf30|91283
C9orf46|55848
CA2|760
CA9|768
CAB39L|81617
CABC1|56997
CACNA1H|8912
CACNA1I|8911
CACNB3|784
CADPS2|93664
CALB2|794
CALD1|800
CAMK1|8536
CAMK2G|818
CAMK2N1|55450
CAP2|10486

CAPN5|726
CAPN9|10753
CAPS|828
CARD10|29775
CARD11|84433
CARD17|440068
CASC4|113201
CASP1|834
CASP14|23581
CASP6|839
CASQ1|844
CASQ2|845
CAV1|857
CAV2|858
CBARA1|10367
CBLN2|147381
CBX7|23492
CBY1|25776
CCDC102A|92922
CCDC106|29903
CCDC109B|55013
CCDC3|83643
CCDC34|91057
CCDC86|79080
CCDC92|80212
CCL15|6359
CCL19|6363
CCL21|6366
CCL3|6348
CCL8|6355
CCNA2|890
CCNB1|891
CCNB2|9133
CCNC|892
CCND2|894
CCNDBP1|23582
CCNE1|898
CCNE2|9134
CCNG1|900
CCNG2|901
CCNYL1|151195
CCR1|1230
CCT6A|908
CCT7|10574
CD109|135228
CD14|929
CD163|9332
CD1A|909
CD248|57124
CD2AP|23607
CD300A|11314
CD34|947
CD44|960
CD46|4179
CD53|963
CD55|1604
CD58|965
CD63|967
CD68|968
CD7|924
CD74|972
CD82|3732
CD83|9308
CD84|8832
CD86|942
CD9|928
CD99L2|83692
CDA|978
CDC25B|994
CDC42|998
CDCA4|55038
CDCA5|113130
CDCA7L|55536
CDCA8|55143
CDCP1|64866
CDH11|1009
CDH13|1012
CDH23|64072
CDH26|60437
CDH3|1001
CDK1|983
CDK20|23552

CDK5R1|8851
CDK6|1021
CDKN3|1033
CDR2|1039
CDSN|1041
CEBPA|1050
CEBPB|1051
CECR7|100130418
CELSR2|1952
CENPA|1058
CENPE|1062
CENPK|64105
CENPL|91687
CENPN|55839
CENPV|201161
CENPW|387103
CERCAM|51148
CERK|64781
CES1|1066
CFB|629
CFD|1675
CFI|3426
CFLAR|8837
CGN|57530
CGNL1|84952
CHCHD7|79145
CHEK1|1111
CHN1|1123
CHN2|1124
CHP2|63928
CHPF|79586
CHPT1|56994
CHRDL2|25884
CHST15|51363
CHST3|9469
CHST7|56548
CHSY3|337876
CILP|8483
CIRBP|1153
CKAP2|26586
CKAP2L|150468
CKAP4|10970
CKS1B|1163
CKS2|1164
CLCA2|9635
CLCF1|23529
CLDN14|23562
CLDN23|137075
CLDN5|7122
CLDN7|1366
CLEC11A|6320
CLEC14A|161198
CLEC2D|29121
CLEC3B|7123
CLEC4A|50856
CLEC5A|23601
CLIC4|25932
CLIC6|54102
CLIP3|25999
CLSTN1|22883
CMTM4|146223
CMTM6|54918
CMTM8|152189
CMYA5|202333
CNGA1|1259
CNIH|10175
CNN1|1264
CNN3|1266
CNTN1|1272
CNTNAP1|8506
COL14A1|7373
COL15A1|1306
COL16A1|1307
COL18A1|80781
COL1A1|1277
COL1A2|1278
COL27A1|85301
COL3A1|1281
COL4A1|1282
COL4A2|1284
COL5A1|1289
COL5A2|1290

COL5A3|50509
COL6A1|1291
COL6A2|1292
COL6A3|1293
COL7A1|1294
COL8A2|1296
COLEC12|81035
COMMD2|51122
COMMD7|149951
COMMD8|54951
COMP|1311
COPZ2|51226
CORO1C|23603
COX19|90639
COX6B1|1340
COX7A1|1346
CPA4|51200
CPE|1363
CPNE8|144402
CPXM1|56265
CPXM2|119587
CRB3|92359
CRBN|51185
CREB3L2|64764
CREB5|9586
CRH|1392
CRISPLD2|83716
CRLF3|51379
CROT|54677
CRY2|1408
CRYAB|1410
CRYL1|51084
CRYZL1|9946
CSDC2|27254
CSF1R|1436
CSPG4|1464
CSRNP1|64651
CSRP1|1465
CSTA|1475
CTGF|1490
CTHRC1|115908
CTPS2|56474
CTSB|1508
CTSC|1075
CTSD|1509
CTSE|1510
CTSF|8722
CTSG|1511
CTSH|1512
CTSK|1513
CTSL1|1514
CTSL2|1515
CTTN|2017
CX3CL1|6376
CXADR|1525
CXCL1|2919
CXCL10|3627
CXCL12|6387
CXCL2|2920
CXCL5|6374
CXorf38|159013
CXorf57|55086
CYB5A|1528
CYBB|1536
CYBRD1|79901
CYCS|54205
CYFIP2|26999
CYGB|114757
CYP26B1|56603
CYP27A1|1593
CYP27B1|1594
CYP2J2|1573
CYP3A5|1577
CYP4B1|1580
CYP4F12|66002
CYP4F22|126410
CYP4F8|11283
CYR61|3491
CYTH1|9267
CYTH4|27128
CYYR1|116159
DACT1|51339

DACT3|147906
DAPK1|1612
DARC|2532
DBI|1622
DBN1|1627
DCAKD|79877
DCBLD1|285761
DCBLD2|131566
DCTPP1|79077
DCUN1D5|84259
DDAH1|23576
DDIT3|1649
DDIT4L|115265
DDR2|4921
DDX21|9188
DDX60|55601
DECR1|1666
DEGS1|8560
DENND1A|57706
DENND2A|27147
DENND2D|79961
DENND4C|55667
DENND5A|23258
DEPDC1|55635
DEPDC6|64798
DES|1674
DFNA5|1687
DGAT2|84649
DHRS11|79154
DHRS2|10202
DIAPH3|81624
DIRC2|84925
DIS3L|115752
DISP1|84976
DIXDC1|85458
DKK3|27122
DKK4|27121
DLC1|10395
DLG4|1742
DLGAP5|9787
DLL1|28514
DMAP1|55929
DNAJA4|55466
DNAJB5|25822
DNAL4|10126
DNALI1|7802
DOCK10|55619
DONSON|29980
DPH3|285381
DPM1|8813
DPM3|54344
DPP7|29952
DPT|1805
DPYD|1806
DPYSL2|1808
DPYSL3|1809
DRAP1|10589
DSC2|1824
DSC3|1825
DSCR6|53820
DSE|29940
DSG3|1830
DSP|1832
DTNA|1837
DUSP14|11072
DUSP3|1845
DYNC1I1|1780
DYNC2LI1|51626
DYSF|8291
E2F7|144455
EBF1|1879
EBF3|253738
ECE2|9718
ECHDC2|55268
ECHS1|1892
ECM1|1893
ECM2|1842
EDARADD|128178
EDNRA|1909
EDNRB|1910
EEF1E1|9521
EFCAB4A|283229

EFEMP1|2202
EGFR|1956
EGR1|1958
EGR2|1959
EHD2|30846
EHF|26298
EIF2AK3|9451
EIF4E3|317649
ELF3|1999
ELF5|2001
ELL2|22936
ELMO1|9844
ELN|2006
ELOVL6|79071
ELP3|55140
EMILIN1|11117
EMP3|2014
EMR2|30817
EMX2|2018
ENG|2022
ENO1|2023
ENPP4|22875
ENPP5|59084
ENSA|2029
ENTPD1|953
ENTPD3|956
EPB41L2|2037
EPCAM|4072
EPDR1|54749
EPHA1|2041
EPHA3|2042
EPN3|55040
EPS8|2059
EPSTI1|94240
ERBB2|2064
ERBB3|2065
ERMP1|79956
ERO1L|30001
ERP27|121506
ESPN|83715
ESRP1|54845
ETFA|2108
ETV5|2119
EVC|2121
EVL|51466
EVPL|2125
EXO1|9156
EXOG|9941
EXT1|2131
EZH2|2146
F12|2161
F3|2152
FAAH|2166
FABP5|2171
FABP6|2172
FADD|8772
FAM101B|359845
FAM107A|11170
FAM107B|83641
FAM108B1|51104
FAM129A|116496
FAM13A|10144
FAM13B|51306
FAM149A|25854
FAM150A|389658
FAM164A|51101
FAM171A1|221061
FAM174B|400451
FAM176A|84141
FAM181B|220382
FAM189A2|9413
FAM196A|642938
FAM198B|51313
FAM20C|56975
FAM38A|9780
FAM3B|54097
FAM43A|131583
FAM46B|115572
FAM57A|79850
FAM59A|64762
FAM63A|55793
FAM64A|54478

FAM65A|79567
FAM69B|138311
FAM83A|84985
FAM84B|157638
FAM91A1|157769
FANCD2|2177
FANCL|55120
FAP|2191
FARP1|10160
FAT2|2196
FBLN1|2192
FBLN5|10516
FBP1|2203
FBXO2|26232
FBXO5|26271
FCER1A|2205
FCER1G|2207
FCGR1A|2209
FCGR1B|2210
FCGR2A|2212
FCHO1|23149
FCN3|8547
FCRLB|127943
FDFT1|2222
FEN1|2237
FER1L4|80307
FERMT2|10979
FERMT3|83706
FFAR2|2867
FGD1|2245
FGD2|221472
FGD3|89846
FGD5|152273
FGF9|2254
FGFBP1|9982
FHL1|2273
FHOD1|29109
FILIP1L|11259
FJX1|24147
FKBP2|2286
FKBP5|2289
FLNC|2318
FLRT2|23768
FLRT3|23767
FMO5|2330
FMO9P|116123
FMOD|2331
FNBP1|23048
FNBP1L|54874
FNDC1|84624
FOSL1|8061
FOXA1|3169
FOXD1|2297
FOXF1|2294
FOXM1|2305
FOXO4|4303
FOXQ1|94234
FPR1|2357
FPR2|2358
FPR3|2359
FRAS1|80144
FRAT2|23401
FREM2|341640
FRMD3|257019
FRMD6|122786
FRZB|2487
FSCN1|6624
FSTL3|10272
FUCA1|2517
FUK|197258
FUT9|10690
FXYD3|5349
FXYD4|53828
FXYD6|53826
FYB|2533
FYCO1|79443
FYN|2534
FZD3|7976
FZD7|8324
G0S2|50486
GABARAPL1|23710
GABBR1|2550

GABBR2|9568
GALNT1|2589
GALNT11|63917
GALNT12|79695
GAMT|2593
GAPDH|2597
GARNL3|84253
GAS1|2619
GAS6|2621
GAS7|8522
GATA2|2624
GATA3|2625
GATA5|140628
GATM|2628
GATS|352954
GBP1|2633
GBP5|115362
GBP6|163351
GCLC|2729
GCLM|2730
GCOM1|145781
GDF15|9518
GDPD3|79153
GEM|2669
GFI1|2672
GFOD1|54438
GFPT2|9945
GGCT|79017
GGCX|2677
GGH|8836
GGT6|124975
GIMAP4|55303
GIMAP5|55340
GIMAP8|155038
GJB2|2706
GK|2710
GLB1L2|89944
GLIPR1|11010
GLIPR2|152007
GLT25D1|79709
GLT8D2|83468
GLTP|51228
GLTSCR2|29997
GMCL1|64395
GMFG|9535
GNA15|2769
GNB4|59345
GNG4|2786
GNL3|26354
GNLY|10578
GOLT1A|127845
GPBAR1|151306
GPC1|2817
GPD1L|23171
GPER|2852
GPIHBP1|338328
GPM6B|2824
GPR110|266977
GPR115|221393
GPR124|25960
GPR160|26996
GPR162|27239
GPR37|2861
GPR68|8111
GPR81|27198
GPR89B|51463
GPRC5C|55890
GPSM1|26086
GPSM2|29899
GPX3|2878
GRAMD1A|57655
GRAMD1C|54762
GRAP|10750
GRB10|2887
GRB2|2885
GRB7|2886
GREM1|26585
GRHL3|57822
GRK5|2869
GSDMB|55876
GSDMC|56169
GSN|2934

GSTM1|2944
GSTM3|2947
GSTM4|2948
GSTO1|9446
GTF2I|2969
GUCA2A|2980
GUCY1A3|2982
GYG2|8908
GYPC|2995
H2AFX|3014
H2AFY|9555
H3F3B|3021
HACL1|26061
HADH|3033
HAND1|9421
HAPLN3|145864
HAS3|3038
HAVCR2|84868
HBEGF|1839
HCFC1R1|54985
HCG4|54435
HCK|3055
HCP5|10866
HCST|10870
HDC|3067
HEG1|57493
HEPH|9843
HERC5|51191
HERC6|55008
HES1|3280
HES2|54626
HEYL|26508
HIBCH|26275
HIC2|23119
HIF1A|3091
HIGD1A|25994
HIPK2|28996
HIST1H2BK|85236
HIST3H2A|92815
HJURP|55355
HK3|3101
HLA-A|3105
HLA-B|3106
HLA-E|3133
HLA-F|3134
HLA-G|3135
HLA-H|3136
HLTF|6596
HM13|81502
HMGA1|3159
HMGB3|3149
HMGCS2|3158
HMMR|3161
HNF1B|6928
HNMT|3176
HNRNPA1|3178
HNRPDL|9987
HOMER2|9455
HOPX|84525
HOXA13|3209
HOXA5|3202
HOXB5|3215
HOXD10|3236
HPGD|3248
HPRT1|3251
HPS5|11234
HPSE|10855
HS3ST3A1|9955
HS6ST2|90161
HSCB|150274
HSD17B11|51170
HSD17B6|8630
HSPA12A|259217
HSPA12B|116835
HSPA4L|22824
HSPA6|3310
HSPA8|3312
HSPB2|3316
HSPB6|126393
HSPB7|27129
HSPB8|26353
HTRA1|5654

HYAL1|3373
ICA1|3382
IDH1|3417
IER3|8870
IER5|51278
IFFO1|25900
IFI16|3428
IFI27|3429
IFI27L2|83982
IFI30|10437
IFI35|3430
IFI44|10561
IFIH1|64135
IFIT1|3434
IFIT2|3433
IFIT3|3437
IFITM1|8519
IFITM2|10581
IFITM3|10410
IFT122|55764
IFT27|11020
IGDCC3|9543
IGF1|3479
IGF2BP2|10644
IGF2R|3482
IGFBP3|3486
IGFBP5|3488
IGFBP6|3489
IGFL1|374918
IGSF11|152404
IGSF6|10261
IL11RA|3590
IL15|3600
IL17D|53342
IL18BP|10068
IL1B|3553
IL1F9|56300
IL1R2|7850
IL1RAP|3556
IL1RN|3557
IL20RB|53833
IL24|11009
IL27RA|9466
IL28B|282617
IL32|9235
IL4I1|259307
IL4R|3566
IL6|3569
IL7R|3575
IL8|3576
ILDR1|286676
ILF2|3608
ILK|3611
ILVBL|10994
IMPDH1|3614
INA|9118
INMT|11185
INPPL1|3636
IP6K2|51447
IPPK|64768
IQCB1|9657
IRAK3|11213
IRF7|3665
ISG20|3669
ISLR|3671
ISYNA1|51477
ITGA3|3675
ITGA5|3678
ITGA6|3655
ITGA8|8516
ITGAM|3684
ITGAX|3687
ITGB2|3689
ITGB5|3693
ITM2C|81618
ITPKA|3706
ITPR3|3710
ITPRIP|85450
ITSN1|6453
JAG1|182
JAG2|3714
JAM2|58494

JAM3|83700
JARID2|3720
JAZF1|221895
JPH2|57158
JUN|3725
KANK1|23189
KANK2|25959
KATNAL1|84056
KBTBD11|9920
KCNAB1|7881
KCNG1|3755
KCNJ8|3764
KCNMA1|3778
KCNMB1|3779
KCTD12|115207
KCTD14|65987
KCTD15|79047
KDELR2|11014
KIAA0101|9768
KIAA0247|9766
KIAA0922|23240
KIAA1244|57221
KIAA1324L|222223
KIAA1370|56204
KIAA1467|57613
KIAA1522|57648
KIAA1598|57698
KIAA1644|85352
KIAA1949|170954
KIF11|3832
KIF14|9928
KIF1B|23095
KIF20A|10112
KIF23|9493
KIF4A|24137
KIF5C|3800
KIFC1|3833
KLC3|147700
KLF13|51621
KLF2|10365
KLF9|687
KLHDC9|126823
KLHL22|84861
KLHL24|54800
KLHL3|26249
KLHL5|51088
KLK10|5655
KLK11|11012
KLK12|43849
KLK13|26085
KLK5|25818
KLK6|5653
KLK7|5650
KLK8|11202
KPNA2|3838
KREMEN2|79412
KRT1|3848
KRT14|3861
KRT16|3868
KRT18|3875
KRT19|3880
KRT20|54474
KRT34|3885
KRT5|3852
KRT6A|3853
KRT6B|3854
KRT6C|286887
KRT7|3855
KRT8|3856
KRT81|3887
LAG3|3902
LAMA1|284217
LAMA3|3909
LAMA4|3910
LAMA5|3911
LAMB1|3912
LAMB2|3913
LAMC2|3918
LAMP2|3920
LAP3|51056
LAPTM5|7805
LARGE|9215

LARP1B|55132
LARP6|55323
LAT2|7462
LAYN|143903
LCOR|84458
LDB2|9079
LDHA|3939
LEPR|3953
LEPRE1|64175
LEPREL2|10536
LFNG|3955
LGALS1|3956
LGALS7|3963
LHFP|10186
LILRA3|11026
LILRA5|353514
LILRB2|10288
LILRB3|11025
LILRB4|11006
LIMCH1|22998
LIMS2|55679
LMBRD1|55788
LMCD1|29995
LMF2|91289
LMNA|4000
LMNB2|84823
LMO3|55885
LMOD1|25802
LOC100133331|100133331
LONP2|83752
LOX|4015
LPAR1|1902
LPAR2|9170
LPAR5|57121
LPP|4026
LRFN2|57497
LRFN4|78999
LRFN5|145581
LRIG1|26018
LRMP|4033
LRP11|84918
LRP5|4041
LRP8|7804
LRRC1|55227
LRRC17|10234
LRRC32|2615
LRRC8A|56262
LRRC8C|84230
LSM12|124801
LSM3|27258
LTB4R|1241
LTB4R2|56413
LTBP4|8425
LY6D|8581
LY86|9450
LY96|23643
LYAR|55646
LYL1|4066
LYN|4067
LYNX1|66004
LYPD6|130574
LYPLA1|10434
LYPLA2P1|653639
LYRM1|57149
LZTFL1|54585
LZTS1|11178
MAD2L1|4085
MAD2L2|10459
MAF|4094
MAL|4118
MAL2|114569
MAMDC2|256691
MAN1C1|57134
MAN2B2|23324
MANSC1|54682
MAOA|4128
MAOB|4129
MAP1A|4130
MAP1B|4131
MAP2K1|5604
MAP3K6|9064
MAP4K3|8491

MAP7D1|55700
MAPK10|5602
MAPK13|5603
MAPKAP1|79109
MAPRE2|10982
MATN2|4147
MBNL2|10150
MCCC1|56922
MCCC2|64087
MCL1|4170
MCM4|4173
MCM7|4176
MCTP1|79772
ME1|4199
ME3|10873
MECOM|2122
MED27|9442
MEF2C|4208
MEG3|55384
MEIS1|4211
MEIS2|4212
MEIS3P1|4213
MELK|9833
MEST|4232
METRNL|284207
METTL7A|25840
MFAP2|4237
MFAP4|4239
MFAP5|8076
MFGE8|4240
MFI2|4241
MFNG|4242
MGAT3|4248
MGAT4A|11320
MGAT4B|11282
MGLL|11343
MGMT|4255
MGP|4256
MGST1|4257
MGST2|4258
MICAL1|64780
MICB|4277
MLKL|197259
MMEL1|79258
MMP1|4312
MMP11|4320
MMP12|4321
MMP23A|8511
MMP23B|8510
MMP25|64386
MMP7|4316
MMP9|4318
MN1|4330
MNDA|4332
MOBKL2A|126308
MOBKL2B|79817
MOCS1|4337
MORN2|729967
MOSC1|64757
MOV10|4343
MOXD1|26002
MPP1|4354
MPP6|51678
MPRIP|23164
MPZL2|10205
MRGPRF|116535
MRPL3|11222
MRPL36|64979
MRPS21|54460
MRPS25|64432
MRPS30|10884
MRS2|57380
MSC|9242
MSI2|124540
MSN|4478
MSRB2|22921
MSRB3|253827
MST1|4485
MST4|51765
MSX2|4488
MT1A|4489
MT1E|4493

MT1F|4494
MT1G|4495
MT1IP|644314
MT1M|4499
MT1X|4501
MT2A|4502
MTFR1|9650
MTHFD2|10797
MTMR11|10903
MTUS1|57509
MUC20|200958
MUSK|4593
MUSTN1|389125
MXD4|10608
MXRA5|25878
MXRA7|439921
MXRA8|54587
MYADM|91663
MYC|4609
MYCL1|4610
MYCN|4613
MYEOV|26579
MYH11|4629
MYL9|10398
MYLIP|29116
MYLK|4638
MYO10|4651
MYO1B|4430
MYO1G|64005
MYO5A|4644
MYO5B|4645
MYO5C|55930
MYOM1|8736
MYOM2|9172
N4BP2L2|10443
NACAD|23148
NADSYN1|55191
NAV1|89796
NBL1|4681
NCALD|83988
NCAM1|4684
NCAPG|64151
NCAPG2|54892
NCF1|653361
NCK1|4690
NCOA1|8648
NCOA7|135112
NCOR2|9612
NCRNA00152|112597
NCS1|23413
NDC80|10403
NDFIP2|54602
NDN|4692
NDRG2|57447
NDST1|3340
NEDD4L|23327
NEDD9|4739
NEIL3|55247
NEK6|10783
NELF|26012
NES|10763
NET1|10276
NETO2|81831
NEXN|91624
NFE2L1|4779
NFIA|4774
NFIB|4781
NFIC|4782
NFIX|4784
NFXL1|152518
NGLY1|55768
NHP2L1|4809
NHS|4810
NINJ1|4814
NINJ2|4815
NIP7|51388
NIPAL1|152519
NIPSNAP1|8508
NISCH|11188
NLRC5|84166
NLRP3|114548
NMB|4828

NMD3|51068
NME1|4830
NME2|4831
NME3|4832
NMT2|9397
NMU|10874
NNMT|4837
NOD2|64127
NOS3|4846
NOSTRIN|115677
NOTCH1|4851
NOTCH4|4855
NOV|4856
NOX4|50507
NPAS2|4862
NPC1|4864
NPRL2|10641
NPTX1|4884
NR1H3|10062
NR2F2|7026
NR2F6|2063
NR3C2|4306
NRARP|441478
NRG1|3084
NRXN2|9379
NT5C2|22978
NT5E|4907
NTAN1|123803
NTN4|59277
NUAK1|9891
NUAK2|81788
NUDT11|55190
NUMA1|4926
NUP155|9631
NUP188|23511
NUP50|10762
NUPR1|26471
NUSAP1|51203
NXN|64359
OAF|220323
OAS2|4939
OASL|8638
OBFC2A|64859
ODZ3|55714
OGN|4969
OIP5|11339
OLFM1|10439
OLFML1|283298
OLFML2B|25903
OLFML3|56944
OPN3|23596
OR13A1|79290
OR7E91P|79315
ORC6L|23594
ORMDL3|94103
OSBP2|23762
OSBPL5|114879
OSCAR|126014
OSM|5008
OSMR|9180
OSR1|130497
OSTC|58505
OSTF1|26578
OVGP1|5016
OVOL2|58495
OXCT1|5019
OXR1|55074
P2RX1|5023
P2RY6|5031
P4HA2|8974
P4HTM|54681
PABPC1|26986
PABPC3|5042
PABPC4|8761
PACSIN1|29993
PADI3|51702
PAFAH1B3|5050
PAICS|10606
PAK2|5062
PALLD|23022
PALM|5064
PAM|5066

PAMR1|25891
PANX1|24145
PAPLN|89932
PAPSS2|9060
PAQR4|124222
PAQR7|164091
PAQR8|85315
PARD6G|84552
PARM1|25849
PARP10|84875
PARP12|64761
PARP9|83666
PARVB|29780
PARVG|64098
PBX1|5087
PC|5091
PCBD1|5092
PCDH17|27253
PCDH18|54510
PCDH7|5099
PCMTD1|115294
PCNA|5111
PCNX|22990
PCOLCE|5118
PCOLCE2|26577
PCP4|5121
PCP4L1|654790
PCSK5|5125
PDCD10|11235
PDE1A|5136
PDE4B|5142
PDE5A|8654
PDE7B|27115
PDE9A|5152
PDGFC|56034
PDGFD|80310
PDGFRA|5156
PDGFRB|5159
PDIK1L|149420
PDK4|5166
PDLIM3|27295
PDLIM7|9260
PDPN|10630
PDSS1|23590
PDZK1IP1|10158
PEBP1|5037
PECAM1|5175
PEG3|5178
PEPD|5184
PEX13|5194
PFKFB3|5209
PFKP|5214
PGAM1|5223
PGAM4|441531
PGAM5|192111
PGAP1|80055
PGAP3|93210
PGCP|10404
PGF|5228
PGK1|5230
PGM1|5236
PGM2|55276
PGM2L1|283209
PGM5|5239
PGRMC2|10424
PHLDA1|22822
PHLDA2|7262
PHLDB1|23187
PHOSPHO2|493911
PHYH|5264
PI16|221476
PI3|5266
PI4KA|5297
PI4KAP1|728233
PI4KAP2|375133
PICK1|9463
PIGF|5281
PIGM|93183
PIGN|23556
PIGQ|9091
PIK3C2B|5287
PIK3CD|5293

PIK3IP1|113791
PIK3R1|5295
PIM1|5292
PIR|8544
PITX1|5307
PJA2|9867
PKD1|5310
PKHD1|5314
PKIA|5569
PKM2|5315
PKMYT1|9088
PKP1|5317
PLA2G2F|64600
PLA2G4C|8605
PLA2G4E|123745
PLA2G7|7941
PLAC9|219348
PLAU|5328
PLAUR|5329
PLCB1|23236
PLCD1|5333
PLCE1|51196
PLCL2|23228
PLEC|5339
PLEK|5341
PLEK2|26499
PLEKHA4|57664
PLEKHA6|22874
PLEKHA7|144100
PLEKHB2|55041
PLEKHG6|55200
PLEKHH1|57475
PLEKHO1|51177
PLEKHO2|80301
PLIN3|10226
PLIN4|729359
PLK4|10733
PLLP|51090
PLN|5350
PLOD1|5351
PLOD2|5352
PLS1|5357
PLSCR4|57088
PLXDC2|84898
PLXNB1|5364
PM20D1|148811
PMAIP1|5366
PMEPA1|56937
PNCK|139728
PNP|4860
PNPLA7|375775
PNRC2|55629
PODN|127435
PODXL|5420
POGK|57645
POLE2|5427
POLR2J4|84820
POSTN|10631
PPAP2A|8611
PPARG|5468
PPFIA1|8500
PPFIBP2|8495
PPIL1|51645
PPIL5|122769
PPM1H|57460
PPOX|5498
PPP1CA|5499
PPP1R13B|23368
PPP1R14A|94274
PPP1R14C|81706
PPP1R3C|5507
PPP1R9A|55607
PPP2R2B|5521
PPP2R2C|5522
PPP4R1|9989
PPPDE2|27351
PRC1|9055
PRDM1|639
PRDX2|7001
PRF1|5551
PRIC285|85441
PRICKLE1|144165

PRIM1|5557
PRIM2|5558
PRKCD|5580
PRKCDBP|112464
PRKCQ|5588
PRKD1|5587
PRND|23627
PRNP|5621
PROK2|60675
PRR11|55771
PRR15L|79170
PRR5L|79899
PRRG2|5639
PRRT2|112476
PRRX1|5396
PRSS16|10279
PRUNE2|158471
PSCA|8000
PSMA1|5682
PSMA6|5687
PSMB8|5696
PSMB9|5698
PSMC4|5704
PSMD1|5707
PSMD12|5718
PSMD7|5713
PSMG2|56984
PTCHD1|139411
PTGIS|5740
PTGS1|5742
PTH1R|5745
PTHLH|5744
PTK6|5753
PTMS|5763
PTN|5764
PTP4A1|7803
PTP4A2|8073
PTP4A3|11156
PTPLA|9200
PTPLAD1|51495
PTPN12|5782
PTPRD|5789
PTPRE|5791
PTPRM|5797
PTPRR|5801
PTPRU|10076
PTPRZ1|5803
PTRF|284119
PTTG1|9232
PTTG2|10744
PTTG3P|26255
PVRL4|81607
PWWP2B|170394
PXDN|7837
PYGB|5834
QARS|5859
RAB11A|8766
RAB11FIP1|80223
RAB11FIP4|84440
RAB15|376267
RAB17|64284
RAB23|51715
RAB25|57111
RAB31|11031
RAB32|10981
RAB34|83871
RAB38|23682
RAB3IL1|5866
RAB3IP|117177
RAB5A|5868
RAB9B|51209
RAC2|5880
RACGAP1|29127
RAD51AP1|10635
RAET1G|353091
RAET1L|154064
RALBP1|10928
RALGAPA2|57186
RALGPS1|9649
RAMP1|10267
RANBP1|5902
RAP1GAP|5909

RARRES1|5918
RARRES2|5919
RASAL3|64926
RASD2|23551
RASL10A|10633
RASL11B|65997
RASL12|51285
RBBP9|10741
RBM18|92400
RBM47|54502
RBMS1|5937
RBP1|5947
RBP7|116362
RBPMS|11030
RBPMS2|348093
RCAN2|10231
RCE1|9986
RCN2|5955
RCN3|57333
REC8|9985
RECK|8434
REEP1|65055
REEP5|7905
REEP6|92840
REL|5966
RENBP|5973
REPIN1|29803
RERE|473
RERG|85004
RFC2|5982
RFC3|5983
RFC4|5984
RFTN1|23180
RFTN2|130132
RGMA|56963
RGS16|6004
RGS2|5997
RGS20|8601
RGS4|5999
RGS5|8490
RHBDF2|79651
RHCG|51458
RHOC|389
RHOD|29984
RHOG|391
RHOJ|57381
RHPN2|85415
RNASE7|84659
RNASET2|8635
RND3|390
RNF128|79589
RNF145|153830
RNF150|57484
RNF19A|25897
RNF19B|127544
RNF217|154214
RNFT1|51136
ROR2|4920
RPA3|6119
RPIA|22934
RPL13A|23521
RPL14|9045
RPL29|6159
RPL36A|6173
RPL6|6128
RPLP0|6175
RPRM|56475
RPS15|6209
RPS21|6227
RPS7|6201
RRAD|6236
RRAGB|10325
RRAS|6237
RRAS2|22800
RRBP1|6238
RRM2|6241
RSAD2|91543
RSRC1|51319
RTN4|57142
RTTN|25914
RUNX1T1|862
RUNX3|864

RXRA|6256
S100A10|6281
S100A13|6284
S100A16|140576
S100A2|6273
S100A3|6274
S100A4|6275
S100A6|6277
S100A7|6278
S100A7A|338324
S100A8|6279
S100A9|6280
S100P|6286
SAA1|6288
SAMD9|54809
SAMD9L|219285
SAMSN1|64092
SAP18|10284
SARM1|23098
SASH1|23328
SAT1|6303
SBK1|388228
SBNO2|22904
SBSN|374897
SC4MOL|6307
SC65|10609
SCAP|22937
SCARA3|51435
SCARA5|286133
SCCPDH|51097
SCD5|79966
SCIN|85477
SCMH1|22955
SCNN1B|6338
SCNN1D|6339
SCNN1G|6340
SCO2|9997
SCRG1|11341
SCUBE2|57758
SDC1|6382
SDPR|8436
SEC14L2|23541
SEH1L|81929
SELENBP1|8991
SEMA5A|9037
SEMA6A|57556
SERINC2|347735
SERP2|387923
SERPINA1|5265
SERPINA3|12
SERPINB1|1992
SERPINB13|5275
SERPINB2|5055
SERPINB3|6317
SERPINB4|6318
SERPINB7|8710
SERPINB8|5271
SERPINE1|5054
SERPINE2|5270
SERPINF1|5176
SERPING1|710
SERPINH1|871
SETBP1|26040
SETMAR|6419
SFN|2810
SFRP2|6423
SFT2D1|113402
SFXN1|94081
SGCA|6442
SGK1|6446
SGOL1|151648
SGOL2|151246
SGPL1|8879
SGSM1|129049
SH2B3|10019
SH2D3C|10044
SH2D4A|63898
SH2D5|400745
SH3BGRL2|83699
SH3BGRL3|83442
SH3BP4|23677
SH3GL3|6457

SH3GLB2|56904
SH3PXD2B|285590
SH3RF2|153769
SH3TC1|54436
SH3YL1|26751
SHANK3|85358
SHB|6461
SHC1|6464
SHROOM1|134549
SIDT1|54847
SIDT2|51092
SIGLEC10|89790
SIL1|64374
SILV|6490
SIRPA|140885
SKP2|6502
SLA|6503
SLC11A1|6556
SLC14A1|6563
SLC15A3|51296
SLC16A3|9123
SLC16A5|9121
SLC20A1|6574
SLC22A17|51310
SLC22A5|6584
SLC23A2|9962
SLC24A3|57419
SLC25A23|79085
SLC25A24|29957
SLC25A28|81894
SLC25A37|51312
SLC25A38|54977
SLC25A42|284439
SLC25A43|203427
SLC27A2|11001
SLC27A3|11000
SLC28A3|64078
SLC29A3|55315
SLC2A10|81031
SLC2A3|6515
SLC2A8|29988
SLC30A2|7780
SLC31A2|1318
SLC37A1|54020
SLC37A4|2542
SLC38A1|81539
SLC39A14|23516
SLC39A6|25800
SLC39A8|64116
SLC3A2|6520
SLC41A3|54946
SLC43A3|29015
SLC44A2|57153
SLC44A3|126969
SLC44A4|80736
SLC5A1|6523
SLC6A9|6536
SLC7A1|6541
SLC7A5|8140
SLC7A7|9056
SLC9A2|6549
SLC9A4|389015
SLC9A9|285195
SLCO2B1|11309
SLCO3A1|28232
SLFN11|91607
SLIT2|9353
SLIT3|6586
SLITRK6|84189
SLMAP|7871
SLMO2|51012
SLPI|6590
SLTM|79811
SMAD6|4091
SMARCC1|6599
SMARCD3|6604
SMC2|10592
SMOC2|64094
SMOX|54498
SMTN|6525
SNAI2|6591
SNAP91|9892

SNCG|6623
SNRPN|6638
SNX31|169166
SNX4|8723
SNX5|27131
SOBP|55084
SOCS1|8651
SOCS3|9021
SOD2|6648
SORBS1|10580
SORBS3|10174
SORD|6652
SORL1|6653
SOX13|9580
SOX7|83595
SOX9|6662
SP110|3431
SPAG4|6676
SPARC|6678
SPARCL1|8404
SPCS1|28972
SPHK1|8877
SPHK2|56848
SPI1|6688
SPINK1|6690
SPINT1|6692
SPINT2|10653
SPIRE1|56907
SPIRE2|84501
SPON1|10418
SPON2|10417
SPOP|8405
SPRED1|161742
SPRR1B|6699
SPRR2A|6700
SPRR2C|6702
SPRR2D|6703
SPRR2E|6704
SPRR2F|6705
SPRR2G|6706
SPRY1|10252
SPRY2|10253
SPSB1|80176
SPTLC3|55304
SQLE|6713
SRC|6714
SRD5A1|6715
SRI|6717
SRP9|6726
SRPK1|6732
SRPX|8406
SSBP2|23635
SSC5D|284297
SSH1|54434
SSH3|54961
SSPN|8082
ST3GAL4|6484
ST3GAL5|8869
ST5|6764
ST6GALNAC6|30815
STAB1|23166
STAP2|55620
STARD13|90627
STAT1|6772
STAT3|6774
STAT4|6775
STIL|6491
STK36|27148
STK39|27347
STMN1|3925
STOM|2040
STON1|11037
STOX2|56977
STX11|8676
STX2|2054
STXBP1|6812
SULF1|23213
SULF2|55959
SULT2A1|6822
SUMF1|285362
SUN2|25777
SUOX|6821

SUV39H1|6839
SYBU|55638
SYNC|81493
SYNGR1|9145
SYNJ2BP|55333
SYNM|23336
SYNPO2|171024
SYPL1|6856
SYT11|23208
SYT15|83849
SYT17|51760
SYTL2|54843
SYTL4|94121
TAC3|6866
TACC1|6867
TACC2|10579
TADA1|117143
TAGLN|6876
TAGLN3|29114
TAP1|6890
TAP2|6891
TARS|6897
TAX1BP1|8887
TBC1D2|55357
TBC1D23|55773
TBC1D3G|654341
TBC1D9|23158
TBL1X|6907
TBX1|6899
TBX2|6909
TBX3|6926
TBXAS1|6916
TC2N|123036
TCEA2|6919
TCEA3|6920
TCEAL1|9338
TCEAL2|140597
TCEAL3|85012
TCEAL4|79921
TCF21|6943
TCF4|6925
TCIRG1|10312
TDH|157739
TDO2|6999
TEAD4|7004
TEPP|374739
TESC|54997
TFB2M|64216
TFCP2L1|29842
TFDP1|7027
TFF2|7032
TGFA|7039
TGFB1I1|7041
TGFB3|7043
TGFBI|7045
TGFBR3|7049
TGIF1|7050
TGM1|7051
TGM2|7052
TH|7054
THBS2|7058
THBS3|7059
THBS4|7060
THNSL2|55258
THOC3|84321
THOC4|10189
THRA|7067
THY1|7070
THYN1|29087
TIA1|7072
TIAF1|9220
TICAM2|353376
TIGD2|166815
TIMP1|7076
TIMP2|7077
TIMP3|7078
TINAGL1|64129
TJAP1|93643
TJP2|9414
TJP3|27134
TK1|7083
TLE2|7089

TLN1|7094
TLR6|10333
TM4SF1|4071
TM4SF19|116211
TM7SF2|7108
TM9SF2|9375
TMC4|147798
TMC7|79905
TMEM119|338773
TMEM125|128218
TMEM132A|54972
TMEM135|65084
TMEM139|135932
TMEM147|10430
TMEM154|201799
TMEM158|25907
TMEM163|81615
TMEM173|340061
TMEM175|84286
TMEM184A|202915
TMEM185B|79134
TMEM189-UBE2V1|387522
TMEM2|23670
TMEM204|79652
TMEM205|374882
TMEM216|51259
TMEM22|80723
TMEM38B|55151
TMEM45A|55076
TMEM47|83604
TMEM48|55706
TMEM50B|757
TMEM51|55092
TMEM62|80021
TMEM64|169200
TMEM88|92162
TMEM91|641649
TMEM97|27346
TMEM99|147184
TMOD1|7111
TMPO|7112
TMPRSS11D|9407
TMPRSS2|7113
TMTC4|84899
TNC|3371
TNFAIP2|7127
TNFAIP3|7128
TNFAIP6|7130
TNFAIP8L3|388121
TNFRSF10A|8797
TNFRSF12A|51330
TNFRSF14|8764
TNFRSF18|8784
TNFRSF19|55504
TNFRSF1B|7133
TNFRSF21|27242
TNFRSF6B|8771
TNFSF10|8743
TNFSF13B|10673
TNNC1|7134
TNS1|7145
TOB1|10140
TOP2B|7155
TOX2|84969
TOX3|27324
TP53INP1|94241
TP53TG1|11257
TPD52|7163
TPD52L1|7164
TPI1|7167
TPK1|27010
TPM2|7169
TPM4|7171
TPP1|1200
TPST1|8460
TPX2|22974
TRAK1|22906
TRAM2|9697
TRAPPC6A|79090
TRIM2|23321
TRIM21|6737
TRIM24|8805

TRIM47|91107
TRIP13|9319
TRIT1|54802
TRO|7216
TROAP|10024
TRPM4|54795
TRPV2|51393
TRPV6|55503
TSC22D2|9819
TSC22D3|1831
TSEN2|80746
TSHZ2|128553
TSHZ3|57616
TSPAN12|23554
TSPAN13|27075
TSPAN15|23555
TSPAN18|90139
TSPAN4|7106
TSPAN5|10098
TSPAN6|7105
TSPAN8|7103
TSPAN9|10867
TSPO|706
TSPYL2|64061
TSPYL5|85453
TST|7263
TTC39B|158219
TTK|7272
TUBA1C|84790
TUBA4A|7277
TUBB|203068
TUBB2A|7280
TUBB3|10381
TUBB6|84617
TUBG2|27175
TUFT1|7286
TXK|7294
TXNIP|10628
TXNRD2|10587
TYMP|1890
TYROBP|7305
UBA3|9039
UBA7|7318
UBAP1|51271
UBE2C|11065
UBE2E2|7325
UBE2G1|7326
UBE2L6|9246
UBE2T|29089
UBL3|5412
UBQLN1|29979
UBTD1|80019
UCHL1|7345
UCHL5|51377
UCK2|7371
UFC1|51506
UGT1A1|54658
UGT1A6|54578
UGT2B7|7364
UHRF1|29128
UPK1A|11045
UPK1B|7348
UPK2|7379
UPK3A|7380
UPK3B|80761
UQCR10|29796
USP21|27005
UST|10090
VAMP8|8673
WARS|7453
VASH1|22846
WASH3P|374666
VASN|114990
VAT1|10493
WBP1|23559
WBP5|51186
WBSCR22|114049
VCAM1|7412
VCAN|1462
VDAC1|7416
WDR43|23160
WDR6|11180

WDR66|144406
WDR72|256764
WDR86|349136
VEGFC|7424
WFDC1|58189
WFDC5|149708
WFS1|7466
VGLL1|51442
VHL|7428
VIM|7431
VIPR1|7433
WISP1|8840
WISP2|8839
WNT5B|81029
WNT7B|7477
VPS35|55737
VPS37D|155382
VPS41|27072
VRK1|7443
WRNIP1|56897
VSIG2|23584
VSIG4|11326
VSNL1|7447
VTCN1|79679
VWA1|64856
WWC1|23286
VWF|7450
XAF1|54739
XBP1|7494
XPC|7508
XPOT|11260
XRCC3|7517
YEATS4|8089
YME1L1|10730
ZBED2|79413
ZBTB16|7704
ZBTB20|26137
ZBTB46|140685
ZCCHC24|219654
ZCWPW1|55063
ZDHHC1|29800
ZDHHC13|54503
ZEB2|9839
ZFHX3|463
ZHX2|22882
ZIC2|7546
ZMYND11|10771
ZMYND15|84225
ZNF124|7678
ZNF20|7568
ZNF217|7764
ZNF219|51222
ZNF248|57209
ZNF254|9534
ZNF285|26974
ZNF302|55900
ZNF320|162967
ZNF321|399669
ZNF33B|7582
ZNF358|140467
ZNF365|22891
ZNF415|55786
ZNF428|126299
ZNF433|163059
ZNF436|80818
ZNF439|90594
ZNF443|10224
ZNF462|58499
ZNF467|168544
ZNF493|284443
ZNF512B|57473
ZNF521|25925
ZNF532|55205
ZNF573|126231
ZNF585A|199704
ZNF626|199777
ZNF627|199692
ZNF679|168417
ZNF680|340252
ZNF681|148213
ZNF682|91120
ZNF702P|79986

ZNF750|79755
ZNF763|284390
ZNF786|136051
ZNF792|126375
ZNF816A|125893
ZNF823|55552
ZNF827|152485
ZNF91|7644
ZNF93|81931
ZRSR2|8233
ZSCAN16|80345
ZSCAN2|54993
ZWILCH|55055
ZWINT|11130
ZYX|7791

>UNC_classifier
AHNAK2|113146
ALOX5AP|241
BHMT|635
C10orf116|10974
CAPN5|726
CD14|929
CDK6|1021
CHST15|51363
CYP2J2|1573
CYP4B1|1580
EMP3|2014
FAM174B|400451
FAM59A|64762
FAP|2191
FBP1|2203
GATA3|2625
GDPD3|79153
GLIPR1|11010
GPD1L|23171
HMGCS2|3158
MSN|4478
MT1X|4501
MT2A|4502
PALLD|23022
PDGFC|56034
PLEKHG6|55200
PPARG|5468
PPFIBP2|8495
PRKCDBP|112464
PRRX1|5396
RAB15|376267
RNF128|79589
SCNN1B|6338
SCNN1G|6340
SEMA5A|9037
SLC27A2|11001
SLC9A2|6549
SPINK1|6690
TBX2|6909
TMEM97|27346
TMPRSS2|7113
TOX3|27324
TRAK1|22906
TUBB6|84617
UPK1A|11045
UPK2|7379
VGLL1|51442
